# DNA elements tether canonical Polycomb Repressive Complex 1 to human genes

**DOI:** 10.1093/nar/gkad889

**Published:** 2023-10-19

**Authors:** Juan I Barrasa, Tatyana G Kahn, Moa J Lundkvist, Yuri B Schwartz

**Affiliations:** Department of Molecular Biology, Umeå University, 901 87 Umeå, Sweden; Department of Molecular Biology, Umeå University, 901 87 Umeå, Sweden; Department of Molecular Biology, Umeå University, 901 87 Umeå, Sweden; Department of Molecular Biology, Umeå University, 901 87 Umeå, Sweden

## Abstract

Development of multicellular animals requires epigenetic repression by Polycomb group proteins. The latter assemble in multi-subunit complexes, of which two kinds, Polycomb Repressive Complex 1 (PRC1) and Polycomb Repressive Complex 2 (PRC2), act together to repress key developmental genes. How PRC1 and PRC2 recognize specific genes remains an open question. Here we report the identification of several hundreds of DNA elements that tether canonical PRC1 to human developmental genes. We use the term tether to describe a process leading to a prominent presence of canonical PRC1 at certain genomic sites, although the complex is unlikely to interact with DNA directly. Detailed analysis indicates that sequence features associated with PRC1 tethering differ from those that favour PRC2 binding. Throughout the genome, the two kinds of sequence features mix in different proportions to yield a gamut of DNA elements that range from those tethering predominantly PRC1 or PRC2 to ones capable of tethering both complexes. The emerging picture is similar to the paradigmatic targeting of Polycomb complexes by Polycomb Response Elements (PREs) of *Drosophila* but providing for greater plasticity.

## Graphical Abstract

**Graphical Abstract:**
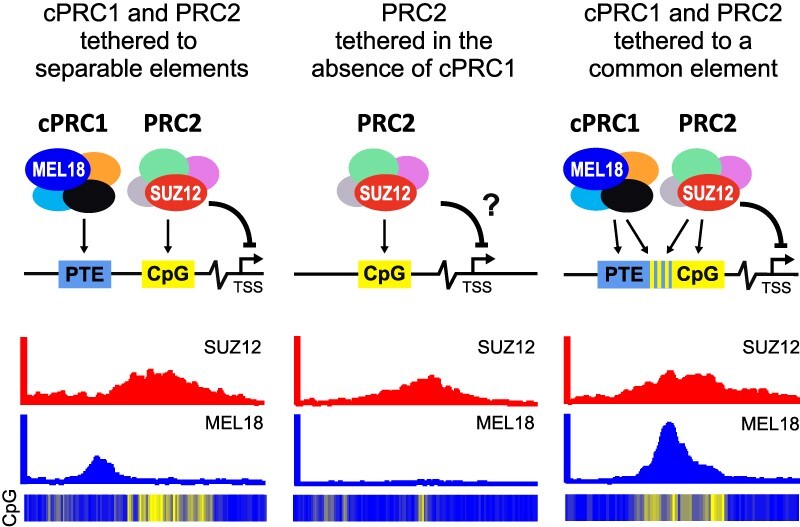

## Introduction

Multicellular animals rely on epigenetic mechanisms to repress alternative gene expression programs when their cells acquire specialized functions. This is common during embryonic development and critical in later life to replenish specific cell pools from multipotent stem cells. Polycomb group proteins make up the epigenetic system most widely used to repress alternative gene expression programs in differentiated cells (1,2).

First discovered in fruit flies *Drosophila melanogaster* as a critical regulator of homeotic selector genes, the Polycomb system was later shown to repress hundreds of genes encoding transcriptional regulators, morphogens, and signalling molecules that are involved in all main developmental pathways (3–5). Many of the same genes are targeted by the system in *Drosophila* and mammalian cells, which suggests that Polycomb group proteins were co-opted to regulate these genes before the insect and vertebrate lineages split.

Early biochemical studies have shown that Polycomb group proteins assemble in two kinds of evolutionarily conserved Polycomb Repressive Complexes, PRC1 and PRC2 (6–10). A mammalian version of PRC1 consists of a heterodimer between RING2 (or its paralogue RING1) and MEL18 or closely related BMI1; a chromodomain-containing subunit represented by either CBX2, CBX4, CBX6, CBX7 or CBX8; one of the Polyhomeotic-like proteins (PHC1, PHC2 or PHC3) and SCMH1 (or its paralogues SCML1, SCML2).

PRC2 complexes contain a core of four subunits: EZH2 (or the closely related EZH1), SUZ12, EED and RBBP7 (or the related protein RBBP4). The PRC2 core may further associate with alternative sets of auxiliary subunits: PHF1 (or its paralogues PHF19 and MTF) (11–14) or JARID2 and AEBP2 (15–18). All PRC2 variants can methylate histone H3 at Lysine 27 (H3K27) and tri-methylation of H3K27 is essential for PRC2 contribution to epigenetic repression by the Polycomb system (19,20).

In addition to the ‘canonical’ complexes described above, the RING1 or RING2 subunits of PRC1 are incorporated in several other complexes sometimes referred to as ‘non-canonical’ or ‘variant’ PRC1 (21–24). Along with RING1 or RING2, these complexes contain RYBP (or the closely related YAF2) protein but lack chromodomain-containing (CBX) and Polyhomeotic-like (PHC) subunits. These complexes may include MEL18 or BMI1 but, predominantly, incorporate one of the closely related PCGF1, PCGF3, PCGF5 or PCGF6 proteins instead. Several constellations of additional subunits distinguish RING–RYBP complexes from each other. Some of these subunits can bind DNA. Once thought to be a vertebrate-specific novelty, the orthologous RING–RYBP complexes were recently identified in *Drosophila* (25) suggesting that both canonical PRC1 and RING–RYBP complexes are evolutionarily old (26). PRC1 and RING–RYBP complexes can monoubiquitylate histone H2A at Lysine 119 although the former appears less active in reactions reconstituted *in vitro* (21,27,28). Multiple lines of evidence argue that canonical PRC1 is critical for the repression of developmental genes (29–34). To what extent the monoubiquitylation of H2A or various RING–RYBP complexes contribute to the repression is a subject of debate (35–42).

Developmental genes repressed by Polycomb mechanisms are bound by canonical PRC1 and PRC2 and enriched in tri-methylated H3K27 (H3K27me3). What molecular mechanisms enable the specific binding of the complexes to these genes is an important open question. Neither canonical PRC1 nor PRC2 have subunits that contain sequence-specific DNA binding domains. In *Drosophila*, genes regulated by the Polycomb system contain specialized Polycomb Response Elements (PREs). These are short (∼1kb) DNA elements, which can be pinpointed from genomic Chromatin Immunoprecipitation (ChIP) profiles as distinct peaks co-occupied by PRC1 and PRC2 (5,43). PREs are sufficient to generate new binding sites for PRC1 and PRC2 when integrated elsewhere in the fly genome. Conversely, their deletions cause stochastic re-activation of the associated genes in cells where those are normally inactive or transcribed at low levels (44,45). Multiple lines of evidence indicate that PRC1 and PRC2 transiently interact with the entire genome (39). PREs may retain the complexes at corresponding chromatin sites (decrease *k*_OFF_) or facilitate their association (increase *k*_ON_).

Which DNA elements direct PRC1 and PRC2 to specific genes in mammals is less clear. Most of what we know about the genomic distribution of mammalian PRC1 and PRC2 comes from studies of mouse embryonic stem cells. In these cells, PRC1 and PRC2 seem to bind repressed genes in a broad and virtually identical fashion with no features to distinguish potential binding DNA elements (37,42,46). Multiple observations indicate that PRC2 prefers to bind at genomic sites enriched in stretches of unmethylated CpG di-nucleotides (47–49). The PCL, JARID2 and AEBP2 subunits of PRC2 have a micromolar affinity to DNA, with a possible preference for CpG (15,50–52), which may contribute to this binding bias (46,53). However, only a fraction of genomic unmethylated CpG-rich sites is bound by PRC2, which argues that other determinants must contribute to the binding specificity.

A handful of DNA elements sufficient to generate binding sites for canonical PRC1 were serendipitously identified within mouse and human genes (54–56). Of those, the 1kb-long DNA element upstream of the human *Cyclin D2* (*CCND2*) gene is particularly interesting (54). It was pinpointed in NT2-D1 embryonic teratocarcinoma cells as a strong localized peak of ChIP enrichment with antibodies against PRC1 subunits but disproportionally weak ChIP signals for PRC2 and H3K27me3. Processes linked to the high transcriptional activity of *CCND2* in the NT2-D1 cells interfere with PRC2 binding to the locus but do not affect the PRC1 binding to the DNA element, which was dubbed a PRC1 Tethering Element (PTE). Here, the term tether describes a process that leads to a prominent presence of canonical PRC1 at genomic sites that harbour such elements. The term does not imply the direct interaction of PRC1 with DNA. We and others speculate that sequence-specific DNA binding adapter proteins may mediate the PRC1 tethering (54–56).

Consistently, in TIG-3 human embryonic fibroblast cells where *CCND2* is repressed by Polycomb mechanisms, the CpG-island adjacent to the *CCND2* PTE shows abundant ChIP-signals for PRC2 and H3K27me3 (54). In these cells, PRC1 is also present at the CpG-island. However, the corresponding ChIP signals are much weaker than those at the PTE. Transgenic experiments showed that the DNA underneath the *CCND2* PTE was sufficient to re-create strong PRC1 binding when integrated elsewhere in the genome. PRC2 was also detectable at the transgenic PTE. However, in contrast to PRC1, ChIP signals for PRC2 and the associated H3K27me3 were low and just above the genomic background. Taken together, these observations argue that the efficient binding of PRC1 and PRC2 to *CCND2* relies on distinct DNA features and that, at this locus, separable DNA elements combine their inputs to achieve the coordinated presence of the two complexes.

How many other developmental genes have PTEs? Are these elements important for the epigenetic repression of the cognate genes? What sequence features promote PRC1 binding? To address these questions, we performed an unbiased genomic screen, which uncovered PTEs at hundreds of genes encoding regulators of organ development, cell fate commitment, and pattern specification. We found that PTEs repress transcription of associated genes and that DNA sequence features required to tether PRC1 differ from those that drive PRC2 binding.

## Materials and methods

### Cell culture

NTERA-2 (NT2-D1 ATCC® CRL-1973™), TIG-3 (see (54) for more details), 293T (ATCC® CRL-3216™) and HeLa (ATCC® CCL-2™) were cultured in high-glucose DMEM (Gibco) supplemented with 10% of heat inactivated foetal bovine serum (Sigma), penicillin/streptomycin (Gibco) and 1.5 g/l sodium bicarbonate (Sigma) at 37°C in an atmosphere of 5% CO_2_. KBM7-1-55-S2-24 cell line (57) was a gift from Dr Brent Cochran (Tufts University). These cells were cultured in Iscove's Modified Dulbecco's Medium (1×) (Gibco) supplemented with 15% of heat-inactivated foetal bovine serum (Sigma), 1× penicillin/streptomycin (Gibco) and 3.024 g/l sodium bicarbonate at 37°C in an atmosphere of 5% CO_2_. Several subclones were isolated from the original cell line by limited dilution and their karyotypes evaluated as follows. Cells were incubated with colchicine (0.1 μg/ml) for 75 min harvested, incubated in hypotonic solution (KCl 75 mM) for 20 min at 37°C and fixed in methanol/glacial acetic acid (3:1). Metaphase spreads were analysed manually. Two sub-clones (F10 and D4) with the most similar karyotypes [25,XY,+8,t(9;22),add(19q)] were selected and the F10 sub-clone was used for most experiments.

### Lentiviral transgenesis

Two transgenic strategies were used to test whether selected DNA fragments can autonomously recruit PRC1 and/or PRC2 when integrated elsewhere in the genome. Both employed the same approach to discriminate between the endogenous and the transgenic DNA copies. For all tested fragments, we identified small stretches of nucleotide sequences that were well enriched by ChIPs with antibodies against MEL18/BMI1 (for PTEs) or SUZ12 (for MEL18-free PRC2-bound regions) but showed little conservation within mammalian species. We then substituted four of those nucleotides in the transgenic copies to create an annealing site for transgene-specific PCR primers (see Supplementary Figure S1).

**Figure 1.**
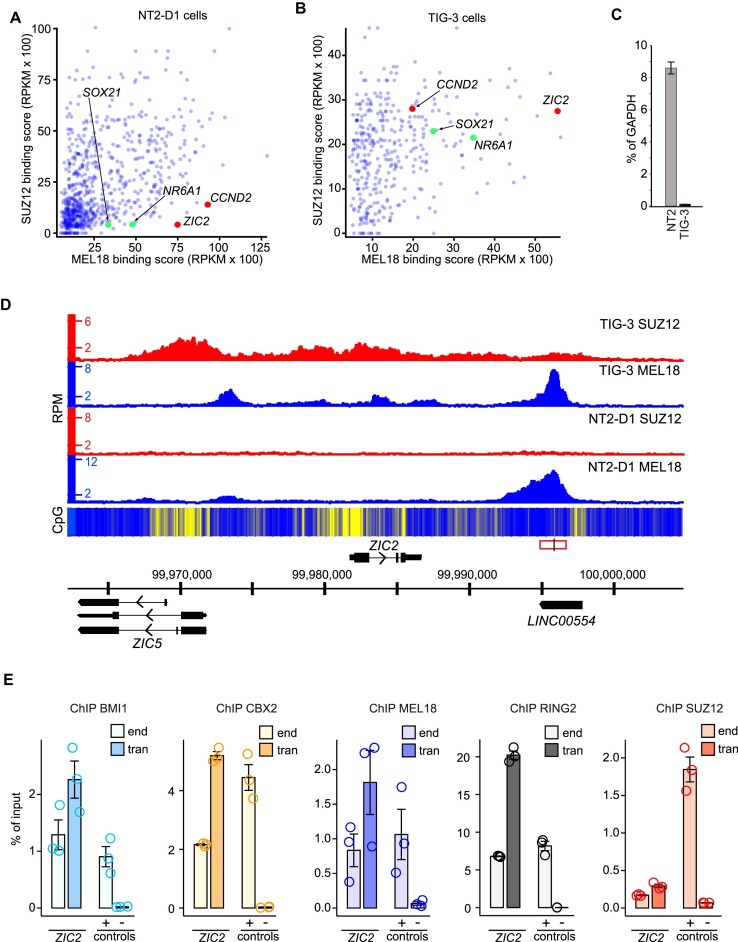
*ZIC2* locus contains a PRC1 tethering element. MEL18 and SUZ12 binding scores for NT2-D1 (**A**) and TIG-3 (**B**) cells at regions significantly enriched by immunoprecipitation of NT2-D1 chromatin with anti-MEL18 antibodies. Each point of the scatter-plot represents a unique region. Regions encompassing *CCND2* (54) and *ZIC2* PTEs are marked in red. *SOX21* and *NR6A1* regions, which also matched our screening criteria, are marked in green. (**C**) RT-qPCR measurements show that the *ZIC2* gene is transcriptionally active in NT2-D1 (NT2) cells but inactive in TIG-3 cells. The bar-plot displays average cDNA counts normalized to the transcription of the housekeeping *GAPDH* gene. Whiskers indicate the scatter between two independent experiments. (**D**) Screen-shot of the MEL18 and SUZ12 ChIP-seq profiles around the *ZIC2* gene in NT2-D1 and TIG-3 cells. The heat-map underneath ChIP-seq profiles shows the number of CpG nucleotides within the 100 bp sliding window (ranging from dark blue = 0 to bright yellow = 15). Here and in other figures, genes above the coordinate scale (in GRCh38/hg38 genomic release) are transcribed from left to right, genes below the coordinate scale are transcribed from right to left. The fragment used for the transgenic experiment in (E) is indicated by a red rectangle, with the genomic position of the ChIP-qPCR amplicon marked by a vertical black line. (**E**) ChIP-qPCR experiments indicate that the DNA underneath the MEL18 peak downstream of the *ZIC2* gene generates a new binding site for CBX2, MEL18, BMI1, and RING2 when integrated elsewhere in the genome. The transgenic DNA (*ZIC2* tran amplicon) immunoprecipitates as efficiently or better as corresponding DNA at the native location (*ZIC2* end amplicon) or a positive control (*ALX4* locus, + control amplicon). Here and on all the following figures, the bar-plots show the average of three independent ChIP experiments performed with three independently prepared batches of chromatin. Circles show individual experimental results and whiskers show standard error of the mean. The CBX2, BMI1, MEL18 and RING2 ChIP signals for transgenic *ZIC2* amplicons are significantly higher (*P* < 0.05, unpaired, one-sided t-test) than that for the negative control ‘spacer’ region (54). For all amplicons, the yields of the mock (no antibody) control ChIPs ranged from 0.008 to 0.07% of input.

The first transgenic strategy, used to test the *ZIC2* PTE (experiments shown on Figure 1D, E), is essentially the same as described by (54). Briefly, the pLenti-ZIC2 PTE 1.8-kb construct was generated by digesting the pLenti-ICR-Puro vector (54) with AfeI and PmlI and recombining it with two overlapping DNA fragments encompassing 1.8 kb of the putative *ZIC2* PTE and with a four base pairs mutation for specific PCR primer. These two PCR fragments were amplified using ZIC2_1.8_PTE_F and ZIC2_trans_R primers, and ZIC2_trans_F and ZIC2_1.8_PTE_R primers, and human genomic DNA as a template. pLenti-ZIC2_mutTCG transgenic construct was generated the same way except that the corresponding 1.8kb DNA fragment was PCR amplified using ZIC2_1.8_PTE_F and ZIC2_mutTCG_2.2 primers, and ZIC2_mutTCG_1.2 and ZIC2_1.8_PTE_R primers and using DNA of the pLenti-ZIC2 PTE 1.8-kb construct as a template. Similarly, for the generation of the pLenti_CCND2_PTE_2xCGmut two PCR fragments were amplified using CCND2_mutTCG_1.1 and CCND2_mutTCG_1.2 primers, and CCND2_mutTCG_2.1 and CCND2_mutTCG_2.2 primers and using DNA of the pLenti CMVTre3G eGFP Puro + ICR -CMV eGFP + 1Kb PTE mut CGA construct (54) as a template.

The second strategy was used to test all other DNA fragments and was higher throughput. To this end, we generated the pLenti-Gateway vector by digesting the pLenti-ICR-Puro vector with AfeI and PmlI and recombining it with the DNA fragment containing Gateway attR cassette. This fragment was PCR amplified using primers Gateway_lenti_F and Gateway_lenti_R and DNA of the pLenti X1 Puro DEST (694–6) vector (gift from Eric Campeau & Paul Kaufman, (58); Addgene plasmid #17297) as a template. The transgenic test constructs were produced in two steps. In the first step, DNA fragments of interest were recombined *in vitro* with pENTR1A no ccdB (w48-1) vector (gift from Eric Campeau & Paul Kaufman, (58); Addgene plasmid #17398) digested with EcoRI using In-Fusion HD system (Clontech). The fragments of interest were PCR amplified using high-fidelity Pfu DNA polymerase (ThermoFisher Scientific), human genomic DNA as a template, and sets of oligonucleotide primers indicated in Supplementary Table S1. In the second step, transgenic fragments were further shuffled into the pLenti-Gateway vector via Gateway LR recombinant reaction using Gateway LR Clonase II Enzyme Mix (Invitrogen). In the case of *KCNA2* and *OVOL1*, the DNA fragments were synthesized by GeneArt (Thermo Fisher Scientific) and cloned into the pMA-RQ vector. The fragments were excised from pMA-RQ constructs using EcoRI digestion, gel purified, and sub-cloned into the pENTR1A no ccdB (w48-1) vector for further shuffling into the pLenti-Gateway vector.

To produce lentiviral viral particles, 5 × 10^6^ 293T cells were plated in a 75-cm2 flask 24 h prior to transfection. Previously described packaging plasmids pCMV-dR8.2dvpr (6 μg) and pCMV-VSV-G (3 μg) (54) were combined with a transgenic construct (9 μg) and co-transfected using X-tremeGene HP (Roche Applied Science) at a 1:3 ratio of DNA to the transfection reagent. After 24 h of incubation, the medium was changed. Lentiviral supernatant was collected after a further 24h incubation, filtered through 0.45 μm filter, and directly used for infection.

For the infection, cells were plated at a confluence of 40–60% 24 h in advance. Viral supernatants were added in serial dilutions to cells in combination with 8 μg/ml Polybrene (Millipore). Cells were subjected to single or multiple infections by adding either single lentiviral supernatant or supernatant pools (up to six different viral supernatants). After overnight incubation, the medium was changed to remove Polybrene. Transduced cells were selected for 14 days by growth in a culture medium supplemented with 1μg/ml puromycin (Invitrogen).

### CRISPR/Cas9-mediated deletion of PTEs

To generate cultured cell lines lacking specific PTEs, the cells of the F10 subclone of the KBM7-1-55-S2-24 line were transfected with the Cas9/gRNA complex using electroporation by Neon Transfection system (Thermo Fisher Scientific) according to manufacture recommendations. Briefly, 2 × 10^5^ cells were mixed with 50pmol of Cas9 protein (IDT) and two gRNAs flanking a PTE sequence (Synthego, 25 pmol each), subjected to an electric pulse (2500V for 10 ms) and plated in IMDM medium without antibiotics supplemented with 15% FBS. The medium was replaced with a complete medium (15% FBS, penicillin, streptomycin) after 24 h, the cells were cultured for 2 more days and re-plated to a 96-well plate after limited dilution to obtain single cell clones. DNA of the recovered clones was subjected to PCR using primers flanking the expected deletion and the nucleotide sequences of PCR products from cells homozygous for deletion alleles were determined by Sanger sequencing. Another PCR analysis was performed using primers annealing to the deleted sequence to confirm the absence of the deleted fragment anywhere in the genome. Nucleotide sequences of the gRNAs and the primers for genotyping are listed in Supplementary Table S2. Genotyping results are shown in Supplementary Figure S2.

### RT-qPCR, ChIP-qPCR and ChIP-seq

RT-qPCR analysis was performed as previously described in (54). The primers used for RT-qPCR analyses are listed in Supplementary Table S3.

ChIP reactions were performed as described in (5,59). The antibodies used for ChIP are listed in Supplementary Table S4 and primers used for qPCR analyses are described in Supplementary Table S5. MEL18 and BMI1 have nearly identical genomic binding profiles (59,60). Highly specific mouse monoclonal antibodies against BMI1 protein became available from the Developmental Studies Hybridoma Bank. We, therefore, used this renewable and cheap reagent, instead of rabbit polyclonal antibody against MEL18, along with antibodies against CBX2 to track PRC1 binding to the transgenes.

Libraries for massively parallel sequencing (ChIP-seq) were prepared with NEBNext Ultra II FS DNA Library Prep Kit for Illumina (E7805) and NEBNext Multiplex Oligos for Illumina Index Primers Set 1 (E7335) according to the manufacturer's instructions. Briefly, 2 ng of ChIP DNAs were treated with NEBNext Ultra II FS Enzyme Mix for 20 min to the average length of 180 bp, followed by adaptor ligation and 8 cycles of PCR amplification. Pooled libraries from MEL18 and SUZ12 immunoprecipitations with chromatins from NT2-D1 and TIG-3 cells were sequenced at NGI Sweden, SciLifeLab, Stockholm. They were sequenced from single end using two lanes (one flow cell) of the Illumina HiSeq2500 instrument operated in rapid 50 bp read mode. Pooled libraries from all immunoprecipitations with chromatin from F10 cells and for immunoprecipitations with antibodies against RING and H3K27me3 and chromatins from NT2-D1 and TIG-3 cells were sequenced by NGI Sweden, SNP&SEQ Technology Platform in Uppsala. They were sequenced from single end using the 100 bp read mode of the Illumina NovaSeq 6000 instrument, v1 sequencing chemistry (Illumina) and two lanes of the SP flow cell.

### Genomic data analysis

#### Definition of bound regions

The sequencing reads were aligned to the human GRCh38/hg38 reference genome using *bowtie2* (v2.2.5) (61) set to *–phred33 -p 8* after which reads with ambiguous genomic positions filtered using *samtools* (v1.3.1) (62) *view* command and the following parameters: *-h -b -@ 8 -q 10*. For each replicate ChIP-seq experiment, significantly enriched regions were identified with *MACS2* (v2.1.2) (63) *callpeak* command using the following parameters: *-f BAM -g hs –broad –min-length 1000 -B –nomodel –extsize 180 –SPMR*. Each significantly enriched region was assigned a binding score, which corresponded to the largest sum of reads within a 1000 bp window included in the region (summit window). Only regions for which the summit window was identified as significantly enriched in both replicate experiments were considered for further analyses. The ‘standalone’ SUZ12 bound regions were defined as those at a distance of >100 kb from any significantly enriched MEL18 region.

#### Definition of MEL18, BMI1 and RING1 binding peaks

For each ChIP-seq data set, bound regions from above were grouped together if separated no further than 100 kb. ChIP-seq signals within each bound region were smoothed using the *geom_smooth* function (method = ‘loess’, span=0.1) of the *ggplot2* package (https://ggplot2.tidyverse.org/). Local maxima (peaks) and local minima (valleys) in the smoothed ChIP-seq signals were identified with *stat_peaks* and *stat_valleys* functions of the same package. Each peak and valley were assigned a score representing the mean ChIP-seq signal over nine sequence positions centered on the corresponding peak or valley. The peaks with scores no lower than 50–60% of the highest peak in the same group and separated by valleys deeper than 30–35% of the scores for the flanking peaks were kept. This peak definition procedure returns similar number of peaks within the above range of parameters. To be conservative, only peaks called at all tested combinations of parameters and identified as significantly enriched in both replicate experiments were used for further analyses. Each of these peaks was assigned a ChIP-seq signal score calculated as the sum of sequencing reads within a 9bp window centered on the peak position.

#### Sequence motif analyses

*STREME* version: 5.3.3 (64) and the following parameters *(–verbosity 1 –oc. –dna –p sequences.fasta –n controls.fasta –minw 4 –maxw 10 –pvt 0.05 –totallength 4000000 –time 14400*) were used to discover 4–10 nucleotide sequence motifs in DNA underneath Q4 peaks defined from the NT2 MEL18 ChIP-seq profile of the replicate experiment with the largest dynamic range. DNA sequences of 110 randomly selected 5kb genomic fragments were used as the control data set. *CentriMo* version 5.4.1 (65) executed with the following parameters (*–oc. –verbosity 1 –score 5.0 –ethresh 10.0 –bfile sequence.fasta.bg sequence.fasta motifs.meme*) was used to calculate the number of motif matches per sequence position within 5kb DNA fragments centered on NT2-MEL18 peaks. The resulting profiles of motif matches were normalized to the total number of matches, divided into quartiles according to the corresponding peak scores, aggregated, and smoothed by a rolling mean of 500 positions.

#### Calculation and plotting of the di-nucleotide frequencies

The di-nucleotide frequencies within 100 bp sliding windows were calculated for 10 kb DNA sequences centered on Q4 MEL18 peaks. To compare those with the genomic average, the same was done for 247 control sequences, and the latter subtracted from the frequencies calculated for DNA around Q4 MEL18 peaks. The control sequences were randomly selected from *GCF_000001405.38_GRCh38.p12_genomic.fna* as long as they did not overlap with the 10kb DNA fragments centered on the NT2-D1 MEL18 peaks (any quartile). The difference between the di-nucleotide frequencies within two independently selected sets of control sequences served as a negative control reference (right panel, Figure 7A).

#### poly(dA)/poly(dT) analysis

The frequencies of poly(dA)_5_ and poly(dT)_5_ stretches within 100 bp sliding windows were calculated for 10kb DNA sequences centered on Q4-Q2 MEL18 peaks. For all instances when the putative target gene was located 5′ (upstream) of the MEL18 peak, the reverse complement sequence was used. Therefore, all target genes were oriented in the same direction, to the right of the peak. 685 control nucleotide sequences of 10kb DNA fragments not overlapping with 10kb DNA fragments centered on the NT2-D1 MEL18 peaks (any quartile) were randomly selected from *GCF_000001405.38_GRCh38.p12_genomic.fna*.

#### MNase-seq data analysis

MNase-seq data from the titration assay of K562 cells (GSE78984, (66)) were downloaded from the Gene Expression Omnibus (GEO) database. The sequencing reads were aligned using *bowtie2* (61) to the *GRCh38_noalt* as index, with default arguments (*bowtie2 -x bowtie_index/GRCh38_noalt_as -1 sequences_1.fastq.gz -2 sequences_2.fastq.gz –phred33 -q -p 8*). The alignments were first converted to bam format using *Samtools* (62) and then normalized for sequencing depth and converted to bed graph format using *MACS2* (v2.1.2) (63) *callpeak* command and the following parameters: *macs2 callpeak -t bam/<file_name>.bam -g hs –outdir macs2_output –bdg –SPMR -n<file_name>_extsize147 –extsize 147 –nomodel -f BAM*. The resulting bed graph files were used to generate average profiles.

#### RNA-seq and Gene Ontology analyses

RNA sequencing data for NT2-D1 (accession # GSM3572746, (67)) and TIG-3 (accession # GSM5137878, (68)) cells were downloaded from the Gene Expression Omnibus (GEO) database. The sequencing reads were aligned using *bowtie2* (61) to the *GRCh38_noalt* as index, with default arguments (*bowtie2 -x bowtie_index/GRCh38_noalt_as -1 sequences_1.fastq.gz -2 sequences_2.fastq.gz –phred33 -q -p 10*). *Samtools* (62) was used to convert the alignments to bam format and the reads were assigned to annotated transcripts using the *featureCounts* function from the *R Bioconductor* package *Rsubread* (69), the inbuilt annotation ‘*hg38*’ and the following parameters: *featureCounts(outfile, annot.inbuilt = ‘hg38’, isPairedEnd=TRUE, nthreads=5, allowMultiOverlap=TRUE, fraction=TRUE, minMQS=20)*. The resulting dataset was further reduced to putative PTE target genes. Those were identified as described in the Results section using gene annotation *GCF_000001405.39_GRCh38.p13_genomic.gff* table available from the National Center for Biotechnology Information (NCBI). Only annotations for which the feature column entry was either ‘mRNA’, ‘gene’ or ‘transcript’, the ‘gbkey’ attribute was ‘mRNA’ or ‘Gene’ and the ‘gene_biotype’ attribute was ‘protein_coding’ or missing were considered.

The Gene Ontology term enrichment was analyzed with *enrichGO* function, from *R Bioconductor* package *clusterProfiler* (70) using the following parameters: *ont = ‘BP’, pvalueCutoff=0.01, qvalueCutoff=0.05, pAdjustMethod = ‘BH’, readable=TRUE*. The gene entries from the NCBI annotation, filtered as described above, were used as a control data set. The *simplify* function was used to reduce the redundancy of the results, which were then plotted with the *cnetplot* function of the *R Bioconductor* package *enrichplot* and the following parameters: *showCategory=5, node_label=‘all’*

## Results

We identified the *CCND2* PTE by genomic mapping of Polycomb group proteins on chromosomes 8, 11 and 12 of human NT2-D1 cells as a site with disproportionally strong ChIP signal for PRC1 but nearly background signals for PRC2 and H3K27me3 (54). The PTE stood out because, in these cells, some processes, linked to the *CCND2* transcription, interfered with PRC2 binding to the locus but did not affect PRC1 binding by the PTE (54). We reasoned that the same approach might reveal additional PTEs if expanded genome-wide. To this end, we mapped MEL18 (a core PRC1 subunit) and SUZ12 (a core PRC2 subunit) binding along the genomes of NT2-D1 and TIG-3 cells using Chromatin Immunoprecipitation coupled to massively parallel sequencing of precipitated DNA (ChIP-seq). We performed two independent ChIP-seq experiments for each cell line and antibody and sequenced the DNA from corresponding chromatin input materials to control for potential sample processing biases.

In preliminary experiments, we noticed that size-selection of immunoprecipitated DNA fragments, commonly used during ChIP-sequencing library preparation, distorts immunoprecipitation profiles compared to those derived from the same ChIP reaction by qPCR (see Supplementary Text for details). We, therefore, omitted the size-selection step and, instead, fragmented immunoprecipitated DNA enzymatically to 180 bp before ligation of adapters for Illumina sequencing.

We then used the ChIP-seq profiles to search the genome of NT2-D1 cells for sites that resemble *CCND2*. That is the sites that: (i) displayed strong MEL18 ChIP-seq signal (>3000 RPKM) comparable to that at the *CCND2* locus, (ii) had the ratio between MEL18 and SUZ12 ChIP-seq signals higher than that at *CCND2*, (iii) showed strong binding of both MEL18 and SUZ12 (both >2000 RPKM) in TIG-3 cells. Three genomic sites, located in the vicinity of the *ZIC2*, *NR6A1* and *SOX21* genes, matched these criteria (Figure 1A, B). Of those, the highest MEL18/SUZ12 ChIP-seq signal ratio corresponded to the site downstream of the *ZIC2* gene. *ZIC2* encodes a human homolog of the *Drosophila* Odd-paired (Opa) transcription factor (71) and is disrupted in ∼5% of holoprosencephaly patients (72).

Like the *CCND2* PTE, the DNA underneath the *ZIC2* MEL18-bound peak is CpG-poor but surrounded by CpG-islands (Figure 1D). RT-qPCR analysis showed that similar to *CCND2*, the *ZIC2* gene is highly transcribed in NT2-D1 cells but transcriptionally inactive in TIG-3 cells (Figure 1C). Further resembling the *CCND2* case, in TIG-3 cells, the binding profiles of MEL18 and SUZ12 profiles differ. The MEL18 signal displays a major peak over the narrow region bound in NT2-D1 cells, with three smaller peaks further upstream. In contrast, the SUZ12 ChIP-seq signals are offset towards the CpG islands (Figure 1D, Supplementary Figure S3). Taken together, our observations fit the hypothesis that the DNA underneath the *ZIC2* MEL18-bound region contains a PTE. If so, we expect the corresponding DNA fragment to generate new PRC1 binding sites when integrated elsewhere in the genome. To test this, we cloned the corresponding 1.8 kb DNA fragment into a lentiviral vector and integrated it back into the genome of NT2-D1 cells. To distinguish the transgenic and endogenous copies, we replaced a small stretch of nucleotides within the cloned DNA fragment to create an annealing site for the transgene-specific PCR primer (Supplementary Figures S1, S4). ChIP-qPCR analysis indicates that the hypothetical PTE does generate new binding sites for MEL18, BMI1, CBX2, and RING2 (Figure 1E) when integrated elsewhere in the genome. Similar to the *CCND2* PTE, the transgenic *ZIC2* PTE is immunoprecipitated with antibodies against SUZ12 just above the background level.

Taken together, our observations argue that screening ChIP-seq profiles for strong PRC1 signals coupled to low signals for PRC2 and high transcriptional activity of the nearby gene is a viable, although inefficient, strategy to find new PTEs. They also confirm our earlier observation that PRC1 and PRC2 may preferentially bind nucleotide sequences in different parts of the gene (54).

### Discrete high-amplitude ChIP-seq peaks mark PRC1 tethering elements

Most of what we know about the genomic binding of mammalian PRC1 and PRC2 comes from studies of mouse embryonic stem cells. There, the two complexes bind similarly and no sites stand out as potential PRC1 tethering elements (37,42,46). Our ChIP-seq profiles from NT2-D1 and TIG-3 cell lines look different. At many genes repressed by Polycomb mechanisms (e.g. *ZIC2* in TIG-3 cells, Figure 1D), PRC1 ChIP-seq profiles have distinct sharp peaks. Could these ChIP-seq peaks mark high-occupancy PRC1 binding elements?

**Figure 2.**
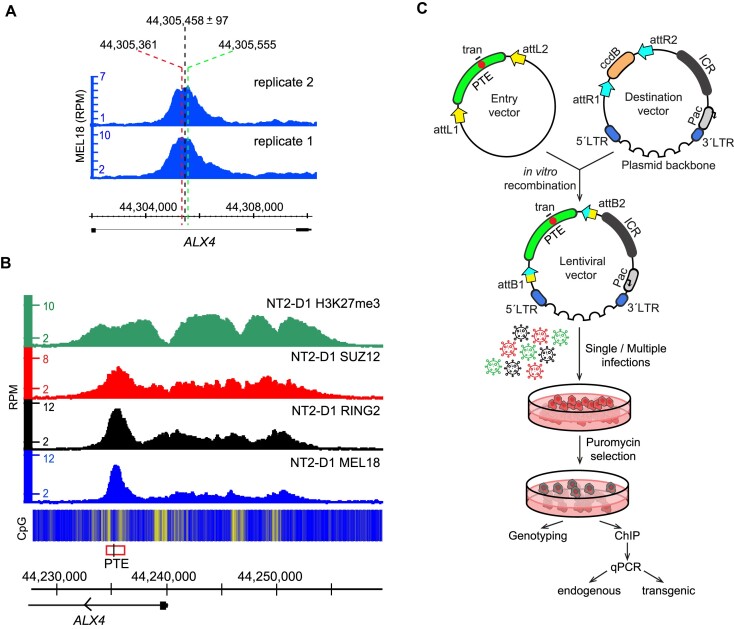
Distinct peaks mark PRC1 tethering elements. (**A**) Assigning genomic positions of candidate PTEs. A screen-shot of replicate MEL18 ChIP-seq profiles around *ALX4* PTE from (B). Positions of corresponding MEL18 peak summits (marked with red and green dashed lines) deviate by 194 bp due to experimental variance. The median genomic position between paired peaks is assigned as the center of candidate *ALX4* PTE. The half distance between the peaks estimates the accuracy of the candidate PTE location. (**B**) H3K27me3, SUZ12, RING2 and MEL18 ChIP-seq profiles over *ALX4* locus, which harbors a candidate PTE (red rectangle, ChIP-qPCR amplicon indicated with black line) marked by obvious MEL18 peak. The heat-map underneath ChIP-seq profiles shows the number of CpG nucleotides within the 100 bp sliding window (ranging from dark blue = 0 to bright yellow = 15). (**C**) The outline of the transgenic assay. To increase the throughput of the assay, the cloning procedure was modified to include Gateway attL/attR recombination (yellow and blue arrows). Cells were transduced with pools of up to six different lentiviral constructs. As in earlier experiments, a small stretch of nucleotides (red circle) within the cloned fragment (green arch) was replaced to distinguish the transgenic and endogenous copies.

To evaluate this possibility, we searched the NT2-D1 MEL18 ChIP-seq profiles for discrete peaks. Briefly, we smoothed the ChIP-seq profiles and identified local maxima with amplitudes exceeding selected cutoffs (see Materials and Methods for a detailed description of the peak-calling algorithm). Using this procedure, we identified 1014 peaks in the profile from the first MEL18 ChIP-seq experiment and 1152 peaks in the profile from the replicate experiment. We expect a genuine tethering element to be marked by a discrete MEL18 peak in each of the replicate profiles. In theory, corresponding peaks from replicate experiments should have the same genomic positions and ChIP-seq signal strength. In practice, both will differ, at least slightly, due to experimental variance. We, therefore, inspected peak sets from the two replicate MEL18 ChIP-seq experiments for pairs with the closest genomic positions. The survey of such pairs indicates that 84% of them have peaks located <500 bp apart, 61%—<250 bp, and in 33% of the cases, they reside as close as 100 bp (Supplementary Figure S5A). As expected for peaks that represent the same MEL18 binding site, their ChIP-seq signal scores correlate (Supplementary Figure S5B). Based on this, we assigned median genomic positions between paired peaks as centers of discrete MEL18 binding sites (potential PTEs) and used the distances between the corresponding paired peaks to reflect our confidence in each location (Figure 2A).

**Figure 3.**
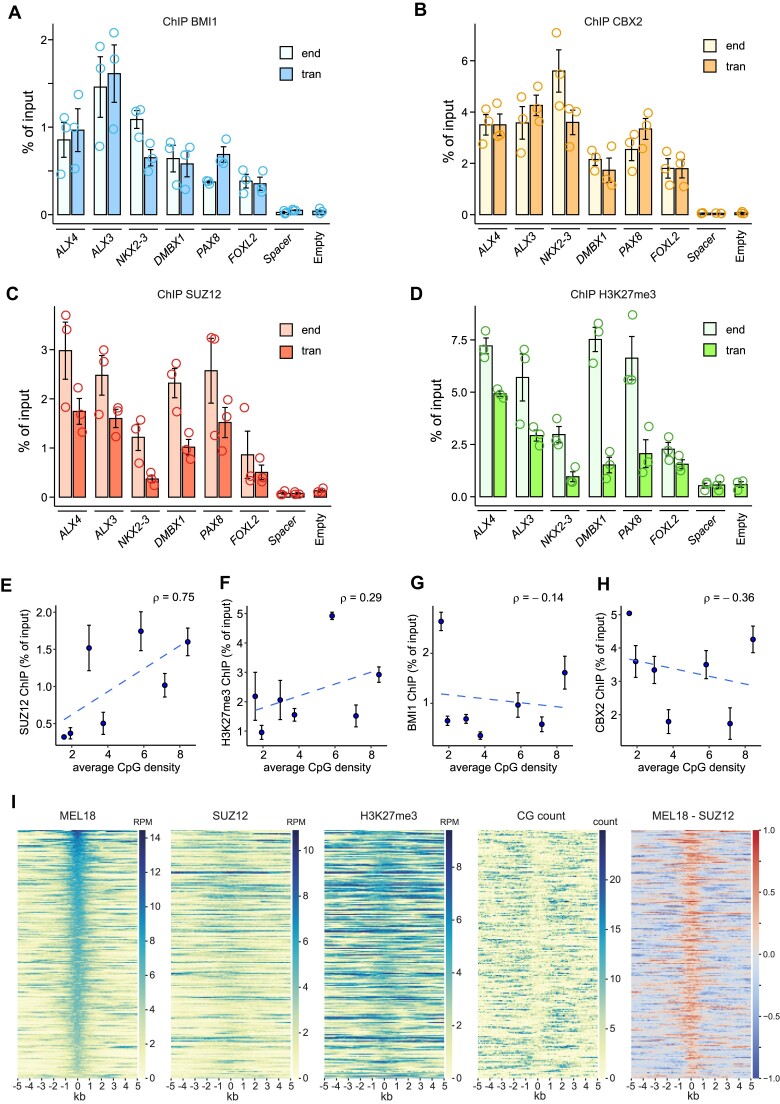
MEL18 Q4 peaks generate new PRC1-binding sites. ChIP-qPCR experiments indicate that DNAs of the PTEs from *ALX4*, *ALX3*, *NKX2.3*, *DMBX1, PAX8* and *FOXL2* loci generate new binding sites for BMI1 (**A**) and CBX2 (**B**) when integrated elsewhere in the genome. The BMI1 and CBX2 ChIP signals for all transgenic fragments (tran) are similar to those at endogenous locations (end). Both are significantly higher (*P* < 0.05, unpaired, one-sided *t*-test) than those for negative controls: the empty vector (empty) and the 2.4 kb ‘spacer’ region. Note that the PCR primers to amplify the ‘spacer’ region do not discriminate between the transgenic and endogenous copies. Because the endogenous copy does not bind PRC1 or PRC2, any elevated ChIP signal in the transgenic cells (tran) would come from the transgene. For clarity, here and in Figures 4 and 6 immunoprecipitation yields for the endogenous *ALX4* amplicon (positive control) and endogenous Spacer amplicon are shown only for one of the transgenic cell lines. The values for other cell lines are comparable. Immunoprecipitation with the antibodies against SUZ12 (**C**) enriches for all transgenic PTEs (*P* < 0.05, unpaired, one-sided t-test). *ALX4*, *ALX3* and *FOXL2* are also significantly enriched by ChIP with the antibodies against H3K27me3 (**D**). For all amplicons, the yields of the mock (no antibody) control ChIP reactions ranged from 0.0002 to 0.036% of input. Scatter plots comparing the average CpG density to the average ChIP-qPCR signals for SUZ12 (**E**), H3K27me3 (**F**), BMI1 (**G**) and CBX2 (**H**) at transgenic PTEs from (A–D) and *ZIC2* (Figure 1E). Whiskers show the standard error of the mean. (**I**) Heat-map representations of ChIP-seq signals, CpG counts and MEL18-SUZ12 ChIP-seq signal differences (rightmost panel) around discrete MEL18 peaks indicate that PRC1 and PRC2 binding profiles differ.

We then split discrete MEL18 binding sites into quartiles based on the associated MEL18 ChIP-signal scores and selected six regions from the upper quartile (Q4). The selected regions had location accuracy of 168bp or better (obvious high and sharp peaks, Figure 2B, Supplementary Figure S6). After cloning the corresponding 1.4–2 kb DNA fragments into a lentiviral vector, we integrated them back into the genome of NT2-D1 cells (Figure 2C, Supplementary Figure S7). ChIP-qPCR indicates that all selected regions generate new PRC1 binding sites when integrated elsewhere in the genome of NT2-D1 cells (Figure 3A, B). The corresponding transgenic and endogenous regions display ChIP signals of similar strength. Taken together with results of transgenic analyses of *CCND2* (54) and *ZIC2* elements, both of which correspond to well defined (± 35 bp) upper quartile (Q4) MEL18 peaks, our observations argue that DNA fragments centered on sharp (±300 bp location accuracy) Q4 MEL18 peaks represent the high-occupancy PTEs.

### The binding of PRC1 and PRC2 is not strictly linked

Unlike PTEs from the *CCND2* and *ZIC2* genes, some of the transgenic PTEs above are also strongly immunoprecipitated with antibodies against SUZ12 and H3K27me3 even when integrated elsewhere in the genome (Figure 3C, D). Consistent with previous observations (48,49,73), the strength of SUZ12 ChIP signal at transgenic PTEs shows a clear correlation with their CpG content. This is not the case for BMI1 and CBX2 (Figure 3E–H). To explore the relation between PRC1 and PRC2 binding further, we compared the ChIP-seq profiles for MEL18, SUZ12, H3K27me3 and CpG di-nucleotide distributions around all putative high-occupancy PTEs (Q4 MEL18 peaks). As illustrated by Figure 3I, in most instances, SUZ12 ChIP signals show no discrete peaks corresponding to those of MEL18. SUZ12 binding is broader, often shifted to the sides, and correlated to higher CpG occurrence around PTEs.

**Figure 4.**
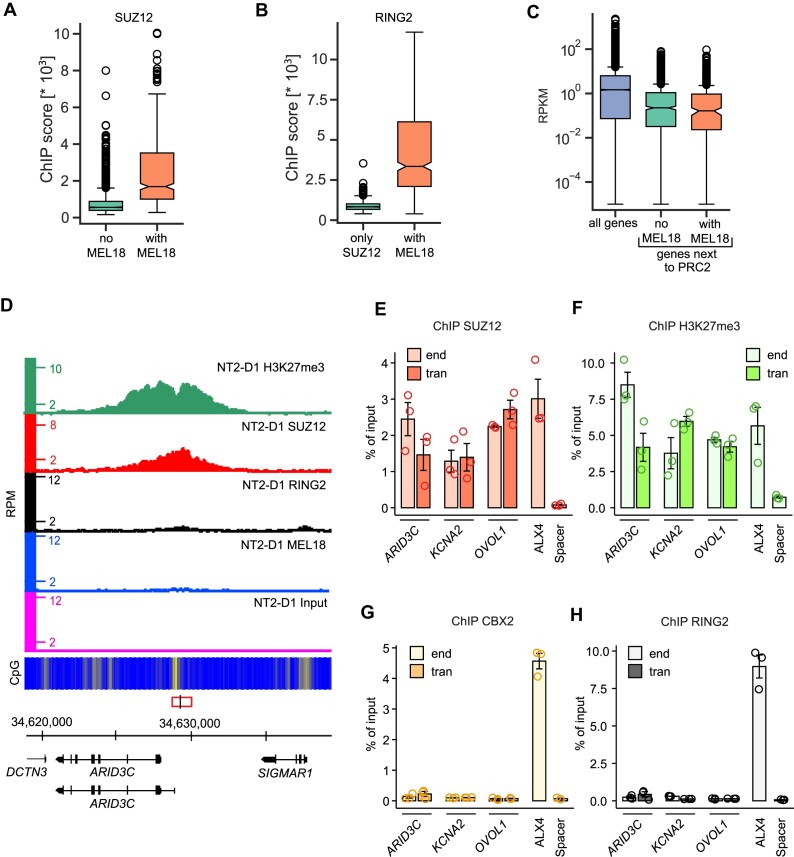
Uncoupled binding of PRC2 and PRC1. (**A**) Box-plots of SUZ12 ChIP-seq signal scores at PRC1-free SUZ12 binding sites and sites with a discrete MEL18 peak nearby. (**B**) Box-plots of RING2 ChIP-seq signal scores at PRC1-free SUZ12 binding sites and within discrete MEL18 peaks. (**C**) Using data from (67), the transcriptional activity of TSSs throughout the genome is compared to that of the TSSs closest to MEL18-free SUZ12 binding sites or SUZ12 binding sites with a discrete MEL18 peak nearby. (**D**) Screen-shot of the MEL18, RING2, SUZ12, H3K27me3 and Input ChIP-seq profiles around the *ARID3C* gene in NT2-D1 cells. The fragment used for transgenic experiments is indicated by the red rectangle, with the genomic position of the ChIP-qPCR amplicon marked by a vertical black line. Profiles show disproportionate signals for SUZ12 and H3K27me3 compared to MEL18 and RING2. ChIP-qPCR experiments indicate that the CpG-rich DNA fragments underneath SUZ12 peaks at *ARID3C*, *KCNA2* and *OVOL1* retain the ability to bind SUZ12 (**E**) and become tri-methylated at H3K27 (**F**) when integrated elsewhere in the genome. However, they do not generate new binding sites for CBX2 (**G**) and RING2 (**H**). For all amplicons, the yields of the mock (no antibody) control ChIPs ranged from 0.0001 to 0.05% of input.

Prompted by this observation, we asked whether the binding of PRC2 and the associated H3K27me3 implies the co-binding of PRC1 at these sites or nearby. Comparison of genomic regions significantly enriched by ChIP with antibodies against SUZ12 and H3K27me3 to those enriched by immunoprecipitation with antibodies against MEL18 and RING2 indicates that this is not always the case. Thus, we detected 1628 regions bound by catalytically active PRC2 but lacking significant ChIP-seq signals for MEL18 within a 10kb distance. At MEL18-free regions, the SUZ12 ChIP-seq signal is generally weaker compared to cases when SUZ12 binds next to discrete MEL18 peaks (Figure 4A). Nevertheless, some of them display SUZ12 ChIP-seq signal comparable to that next to high-occupancy PTEs. (Figure 4A, D). In both kinds of regions, the strength of the SUZ12 signal moderately (ρ ≈ 0.5) correlates with their CpG content. The correlation is weaker than that at the PTE transgenes suggesting that endogenous chromatin context influences PRC2 binding. Regardless of whether MEL18 is bound in the vicinity, the genes closest to SUZ12-bound regions tend to have low transcriptional output (Figure 4C).

Is the information included in the nucleotide sequences of the MEL18-free PRC2-bound regions sufficient to generate new PRC2 binding sites? To address this question, we generated three lentiviral transgenic constructs containing such regions from the *ARID3C*, *KCNA2*, and *OVOL1* loci (Figure 4D, Supplementary Figure S8A-B) and integrated them elsewhere in the genome of the NT2-D1 cells. Strikingly, all three constructs are immunoprecipitated with antibodies against SUZ12 and H3K27me3 but not with antibodies against CBX2, BMI1 or RING2 (Figure 4E–H, Supplementary Figures S8C–F, S9). This suggests that MEL18-free PRC2-bound regions are sufficient to tether PRC2.

Although not confidently identified by qPCR at the three selected sites, ChIP-seq experiments detect RING2 at some of the MEL18-free PRC2-bound regions. The RING2 ChIP-seq signal at these regions is much weaker than that at discrete MEL18 binding peaks (Figure 4B). We speculate that these weak signals represent the binding of RING–RYBP complexes. Alternatively, they may represent exceedingly weak or transient binding of canonical PRC1 detectable only by ChIP with antibodies against RING2.

Regardless, our observations argue that, in the NT2-D1 cells, PRC2 binding and associated tri-methylation of H3K27 is not sufficient to tether canonical PRC1 and, inversely, that the catalytically active PRC2 can be bound to a locus without appreciable co-binding of PRC1. Overall, it appears that PRC1 and PRC2 tethering is driven by distinct DNA sequence determinants. These determinants may be uncoupled or intermixed to a variable degree within a single DNA stretch.

### High-occupancy PTEs repress transcription of the nearby genes

**Figure 5.**
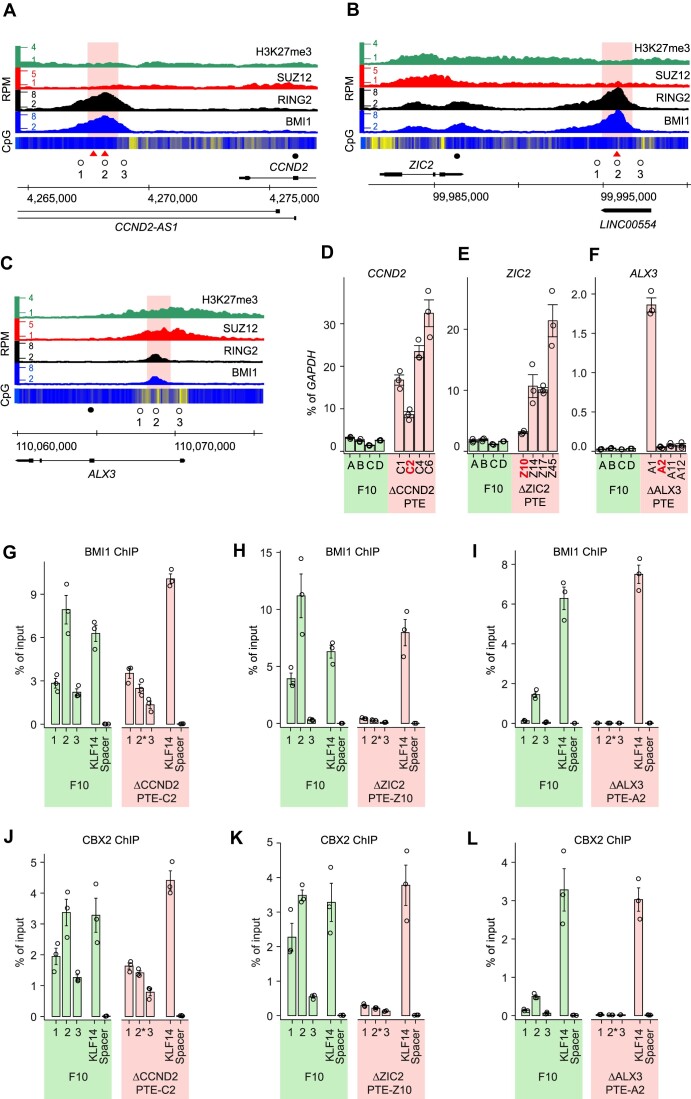
High-occupancy PTEs repress transcription. Screen-shots of BMI1, RING2 SUZ12 and H3K27me3 profiles throughout *CCND2* (**A**), *ZIC2* (**B**) and *ALX3* (**C**) loci in F10 cells. Open circles mark the positions of the PCR amplicons used for ChIP-qPCR, filled circles mark the positions of the RT-qPCR amplicons. Red triangles indicate the positions of the AAACGAAA motifs. The pink shading marks DNA stretches deleted by CRISPR/Cas9-mediated editing. RT-qPCR measurements of *CCND2* (**D**), *ZIC2* (**E**) and *ALX3* (**F**) transcription in four clones of unedited F10 cells (green bars) and four clones of F10 cells with corresponding deletions (pink bars). The clones used for ChIP are marked in red. ChIP-qPCR with antibodies against BMI1 (**G–I**) and CBX2 (**J–L**). Immunoprecipitation of *KLF14* and an intergenic region (Spacer) were used as positive and negative controls, respectively. Note that 2* amplicons span the deletion breakpoints.

Distinct high-amplitude PRC1 ChIP-seq peaks mark high-occupancy PTEs sufficient to generate new PRC1 binding sites when integrated elsewhere in the genome of the NT2-D1 cells. Are these elements necessary for PRC1 binding at their endogenous locations and, more importantly, do they repress transcription? To answer these questions, we sought to delete PTEs from genes bound by both PRC1 and PRC2 using CRISPR/Cas9 – mediated genome editing. Our initial attempts to delete PTEs in NT2-D1 and TIG-3 cells were marred by technical difficulties. NT2-D1 cells are hypotriploid (74) and TIG-3 cells are extremely difficult to clone. To facilitate the genome editing, we turned to the nearly haploid human myeloid leukaemia cell line KBM7-1-55-S2-24 (clonal derivative F10) (57), hereafter referred to as F10. These cells have only a single copy of a chromosomal site to edit and are easy to clone. First, we mapped the distribution of BMI1, RING2, SUZ12 and H3K27me3 throughout the genome of these cells using ChIP-seq. It showed that, in F10 cells, *CCND2*, *ZIC2*, and *ALX3* genes are bound by PRC1, PRC2 and H3K27me3 (Figure 5A-C) and likely repressed by the Polycomb system. We therefore transfected F10 cells with mixtures of the Cas9 protein and gRNAs targeting the *CCND2*, *ZIC2* or *ALX3* PTEs respectively, and derived four clonal cell lines with a precise deletion of the corresponding PTE from each treated cell pool (Supplementary Figure S2).

When comparing a parental cell line to its genetically manipulated clonal derivative a difference in transcriptional output of a gene may stem from genetic manipulation (i.e. PTE deletion) or from individual cell-to-cell variation already present in the population of parental cells and fixed by cloning. To control for the latter, we derived four cell lines by expansion of single cells from the original F10 population (lines F10A, F10B, F10C, F10D). The RT-qPCR measurements show little variation in transcript levels of *CCND2*, *ZIC2* and *ALX3* between the control cell lines (Figure 5D–F). In contrast, all four clonal derivatives lacking the *CCND2* or *ZIC2* PTEs display a variable but significant increase in the RNA levels of the corresponding genes (Figure 5D, E). Only one line with deleted *ALX3* PTE shows higher *ALX3* transcript abundance (Figure 5F). We speculate that F10 cells express the subthreshold quantities of transcription factors required to activate *CCND2* and *ZIC2*. These quantities are not sufficient to drive transcription effectively in the presence of PTEs but become so when PTEs are deleted. In the case of *ALX3*, whose transcription in the unedited F10 cells is essentially undetectable, the levels of corresponding transcriptional activators must be very low. Taken together, our observations argue that deletion of high-occupancy PTEs leads to a stochastic increase in transcription of the closest genes and, therefore, the high-occupancy PTEs are repressive elements.

When PTEs are removed, the high ChIP-signals for BMI1 and CBX2 across corresponding deletion breakpoints disappear (Figure 5G–L). This indicates that the *CCND2*, *ZIC2*, and *ALX3* PTEs are required for efficient tethering of PRC1 at these loci. Interestingly, the deletion of PTEs from *ALX3* and *ZIC2* loci causes a nearly complete loss of PRC1 ChIP signals even in the flanking regions (Figure 5H, I, K, L). This suggests that, in some loci, PTEs are the master determinants of PRC1 binding. In contrast, at *CCND2*, the weaker PRC1 ChIP signals within the flanking regions are unaffected by the PTE deletion (Figure 5G, J). It appears that, in addition to the PTE required for high occupancy binding, *CCND2* contains additional DNA features capable to maintain some degree of PRC1 tethering.

### PRC1 tethering is not limited to high-occupancy PTEs

DNA sequences around the *CCND2* PTE provide some degree of PRC1 tethering. Therefore, we wondered whether weaker (Q3-Q2) MEL18 ChIP-seq peaks from our data set could do the same. More specifically, we wished to ask whether DNA fragments underneath such MEL18 peaks could generate new PRC1 binding sites when integrated elsewhere in the genome.

**Figure 6.**
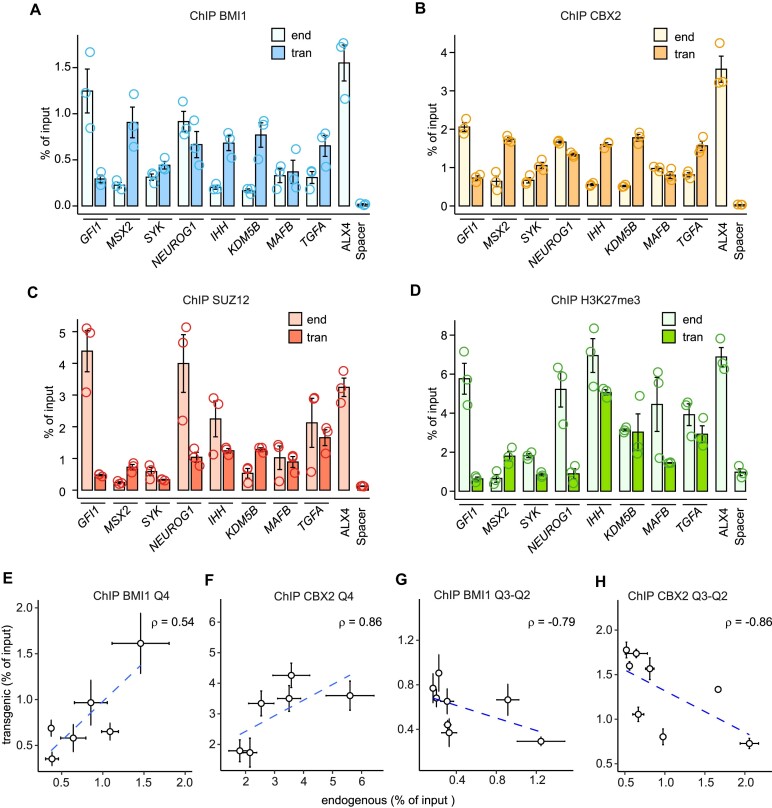
PRC1 tethering by low-occupancy sites. ChIP-qPCR experiments indicate that DNA fragments underneath selected Q3 and Q2 MEL18 peaks always generate new binding sites for BMI1 (**A**) and CBX2 (**B**) when integrated elsewhere in the genome. Sometimes they also generate new binding sites for SUZ12 (**C**) and H3K27me3 (**D**). Immunoprecipitation of *ALX4* and an intergenic region (Spacer) were used as positive and negative controls, respectively. Comparison of BMI1 (**E, G**) and CBX2 (**F, H**) ChIP-qPCR signals at transgenic and endogenous locations indicate that those, corresponding to the high-occupancy PTEs, correlate but those, corresponding to the low occupancy MEL18 peaks, do not. The data for high-occupancy PTEs is from Figure 3A, B. The whiskers show the standard error of the mean for three independent experiments.

To address this question, we selected eight discrete (position accuracy better than ±167 bp) MEL18 peaks from the Q3-Q2 group (Supplementary Figures S10, S11) and re-introduced them in the genome of the NT2-D1 cells (Supplementary Figure S12). ChIP analysis of the resulting transgenic cell lines revealed two trends. First, all transgenic fragments displayed significantly stronger precipitation with anti-BMI1 and anti-CBX2 antibodies (*P*-value < 0.05, unpaired, one-sided *t*-test) compared to the negative control (Figure 6A, B). ChIPs with antibodies against SUZ12 and H3K27me3 were more variable, and some amplicons were not significantly enriched compared to the negative control (Figure 6C, D). Second, in contrast to the high-occupancy PTEs (Figures 3A, B, 6E, F), the signals for the Q3-Q2 fragments at endogenous locations and within the transgene were not positively correlated (Figure 6A–B, G–H).

We take it to indicate that the majority of the DNA fragments underneath Q2 and Q3 MEL18 peaks can tether PRC1 when integrated elsewhere in the genome. However, compared to that of the high occupancy PTEs, the tethering ability is more dependent on the chromatin context. The information contained within the DNA underneath the Q2 and Q3 MEL18 peaks is essential for PRC1 tethering as neither the ‘empty’ backbone nor the constructs containing MEL18-free PRC2-bound CpG-islands or Spacer fragments generate PRC1 binding sites. Yet, our lentiviral transgenic system appears to favor tethering as some of the Q3-Q2 PTEs display stronger PRC1 ChIP-signals inside the transgenes compared to their endogenous locations.

### DNA sequence determinants of PRC1 tethering

**Figure 7.**
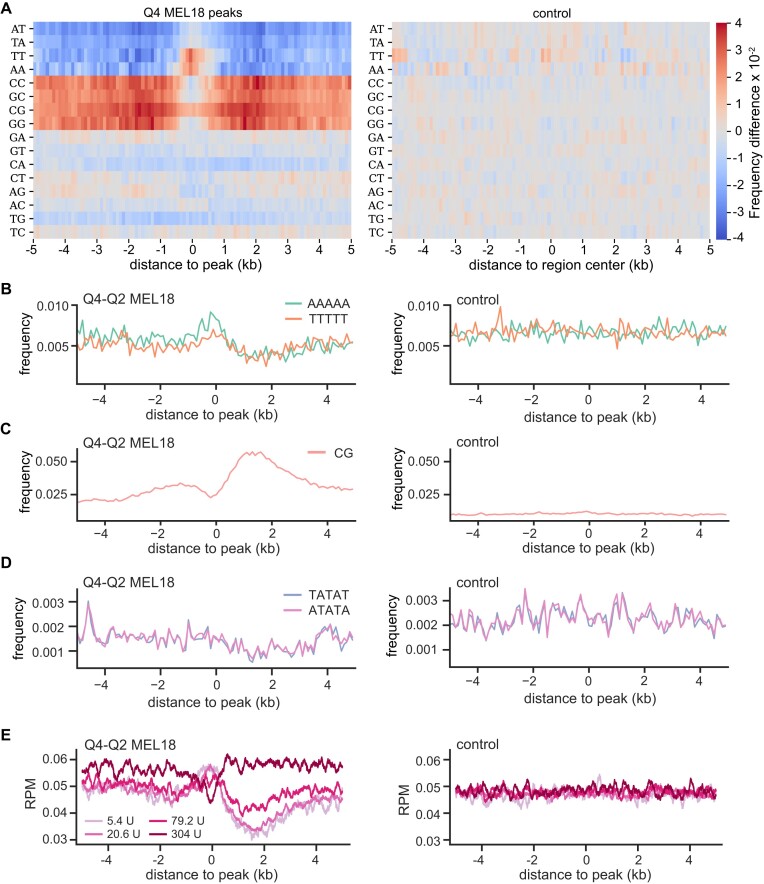
DNA sequence features of PTEs. (**A**) Heat-map representation of the di-nucleotide frequency differences around high-occupancy (Q4) MEL18 peaks and randomly selected control sequences that do not bind PRC1 or PRC2. The control map is produced by calculating the frequency difference between randomly divided sequences of the control group. (**B**) The frequency of poly(dA)_5_ and poly(dT)_5_ within 100 bp windows around MEL18 peaks and within control DNA sequences. CpG frequency (**C**) and the frequencies of TATAT and ATATA oligonucleotides (**D**) for the same DNA sequences as in (B). (**E**) Mean MNase-seq profiles from MNase titration assay by (66) were plotted over the same regions as in (B). The MNase-seq signals are normalized for the sequencing depth. Note that regions underneath MEL18 peaks display increased MNase-seq signal in light-digested samples (treated with 5.4U and 20.6U of MNase) and a substantial reduction of the signal upon deep digestion (samples treated with 304U of MNase).

The information contained within PTEs is in some way encoded in their DNA sequence. What sequence features define this code? To address this issue, we first examined the overall nucleotide composition of the DNA underneath and around discrete MEL18 peaks. As illustrated by Figure 7A, this DNA is enriched in AA and TT di-nucleotides and is flanked by the CpG-rich nucleotide sequences. These nucleotide sequence features are most prominent within MEL18 peaks that display the highest ChIP-signal scores and become progressively less distinct in peaks with lower MEL18 ChIP-signals (Supplementary Figure S13).

Although the DNA underneath the MEL18 peaks is enriched in AA and TT di-nucleotides, its AT or TA content remains close to the genomic average (Figure 7A, Supplementary Figure S13). This suggests that the presence of poly(dA:dT) nucleotides, rather than general AT-richness, in some way helps to tether PRC1. Certain budding yeast promoters have the strand-biased poly(dA) distribution (75), apparently, to take advantage of the directional nucleosome sliding activity of the RSC chromatin remodeling complexes, which is stimulated by the homopolymeric poly(dA:dT) tracts (76,77). To explore this resemblance, we oriented the nucleotide sequences underneath the Q4-Q2 MEL18 peaks such that the TSS of the closest gene was always to the right of the peak. Plotting the frequency of poly(dA)_5_ and poly(dT)_5_ tracts across the oriented DNA sequences revealed two noticeable biases (Figure 7B). First, the DNA underneath MEL18 peaks is enriched in poly(dA)_5_ tracts. Second, the distributions of poly(dA)_5_ and poly(dT)_5_ tracts around the MEL18 peaks differ. Both kinds of tracts become less frequent, compared to the genomic average, in the DNA to the right of the peaks, presumably reflecting its elevated CG content (Figure 7C). In contrast, while the frequency of the poly(dT)_5_ tracts to the left of the MEL18 peaks follows the same trend, the frequency of the poly(dA)_5_ tracts remains high. It appears that selection pressure prevents the poly(dA)_5_ but not the poly(dT)_5_ tracts from being depleted within this CG-rich DNA. The biases are also evident for the DNA under the high-occupancy MEL18/BMI1 peaks identified by ChIP-seq with TIG-3 and F10 cells but not in the control frequency plots for ATATA and TATAT tracts (Figure 7D). The RSC complex (known as PBAF in mammals) is evolutionarily conserved (78). Conceivably, PRC1 tethering by PTEs benefits from the directional sliding of nucleosomes.

Increased nucleosome dynamics can be detected when chromatin is treated by increasing amounts of micrococcal nuclease (MNase) and the release of short DNA fragments (∼150 bp, the size of DNA wrapped around the nucleosome histone core) compared by sequencing (66). In such an assay, regions with dynamic nucleosome exchange display the highest number of short fragments under the light-digestion conditions and progressively lower numbers in deeper-digested samples. In contrast, regions with reduced nucleosome dynamics show the highest number of mono-nucleosome size DNA fragments under the deep-digestion conditions. To evaluate the nucleosome dynamics within the chromatin of MEL18 peaks, we re-analyzed the high-quality data from the MNase titration assay of human K562 cells (66). Consistent with increased nucleosome sliding, regions underneath MEL18 peaks, but not in the control set, display higher MNase-seq signal in light-digested samples and a substantial reduction of the signal upon deep digestion (Figure 7E). The trend is similar to that over dynamic ‘-1’ nucleosome immediately upstream of Transcription Start Sites (TSS) of highly active genes (79), albeit with a notably lower dynamic range (Supplementary Figure S14). Interestingly, the PTE-adjacent CpG-rich chromatin appears considerably more resistant to MNase treatment compared to either PTEs or random control regions.

**Figure 8.**
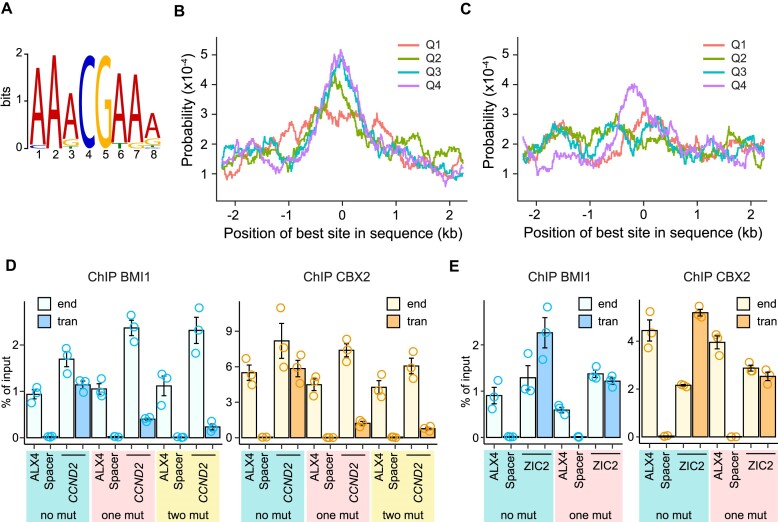
DNA sequence motif contributes to PRC1 tethering by PTEs. (**A**) Web logo representation of the ‘AAACGAAA’ motif. Probabilities to find the best match to the ‘AAACGAAA’ (**B**) or truncated, control ‘AAAGCAAA’ (**C**) motifs around MEL18 peaks of different occupancy (ChIP-seq score quartiles). The results of ChIP-qPCR with chromatin from NT2-D1 cells transduced with various *CCND2* PTE (**D**) and *ZIC2* PTE transgenes (**E**).

Nucleosome displacement may expose binding sites recognized by the sequence-specific DNA binding proteins that act as adaptors to tether of PRC1. To investigate this possibility, we combined the STREAM (64) and CentriMo (65) algorithms implemented in the MEME software suite (80) to identify sequence motifs enriched in the DNA underneath MEL18 peaks and biased towards their summits. Analysis of the 5kb DNA fragments centered on high-occupancy (Q4) MEL18 peaks revealed one such motif that we dubbed ‘AAACGAAA’ (Figure 8A). The motif shows significant preference towards the summits of Q4, Q3 and Q2 MEL18 peaks (Figure 8B). This is not the case for the scrambled ‘AAAGCAAA’ motif, where positions of the C and G nucleotides were swapped but the rest of the position weight matrix describing the motif was left intact (Figure 8C).

Comparison with experimentally defined transcription factor binding sites from JASPAR (81) and HOCOMOCO (82) databases revealed no candidate proteins to bind the ‘AAACGAAA’ motif. Its sequence resembles the previously reported ‘CGA’ motif, which we found enriched in the MEL18/BMI1 binding sites on human chromosomes 8, 11 and 12 (54). We posit that the ‘AAACGAAA’ motif is a better-defined version of ‘CGA’ due to the more numerous and accurately mapped PRC1 binding sites employed in the present analysis.

The *CCND2* PTE contains one AAACGAAA nucleotide sequence, the highest-scoring match to the motif's position weight matrix, located directly underneath of the MEL18 peak summit (Figure 5A). In addition, it harbours the second-best match, AATCGAAA, just at the edge of the 1kb fragment that generates new PRC1 binding sites when integrated elsewhere. Converting the best matching AAACGAAA sequence to AAAAAAAA leads to a four-fold reduced immunoprecipitation of the transgenic *CCND2* PTE with antibodies against BMI1 and CBX2 (Figure 8D, Supplementary Figure S15). Simultaneous conversion of the second best-matching motif has no additional impact on the PRC1 tethering (Figure 8D). The *ZIC2* PTE has no best-matching instances of the motif but has the AATCGAAA sequence located just underneath the MEL18 peak summit (Figure 5B). Conversion of this sequence to AAAAAAAA reduces the immunoprecipitation of the transgenic PTE two-fold (Figure 8E, Supplementary Figure S15). Taken together, our observations argue that the ‘AAACGAAA’ motif plays a significant role in tethering PRC1 to PTEs. Additional studies will be needed to uncover the molecule that recognizes it.

### PRC1 tethering is influenced by processes other than transcription

In the NT2-D1 cells, our algorithm identifies 913 MEL18 peaks of which 396 we consider as putative high-confidence PTEs because they have high (Q4 or Q3) MEL18 ChIP-seq signal score and the position defined with accuracy better than ±301 bp (Supplementary Table S6). What kind of genes are regulated by these PTEs? To address this question, we searched for the nearest Transcription Start Sites (TSS) to the left and to the right of each PTE and asked if these TSS are significantly enriched by immunoprecipitation with antibodies against H3K27me3. In 75% of cases, the chromatin of the closer of the two TSS was enriched in H3K27me3. We took this to indicate that the corresponding gene is the most likely target of the PTE. This is a conservative approach. At some loci, a PTE may affect several genes, which we did not consider for further analyses. In a few cases (4%), the closer of the two TSS was not enriched in H3K27me3, but the other one was. In those instances, the gene corresponding to the latter was designated as the likely PTE target. In 21% of the cases, which included *CCND2* and *ZIC2* PTEs, neither of the nearby TSSs was enriched by H3K27me3 ChIP. In those cases, the gene with the TSS closest to the PTE was designated as the likely target.

**Figure 9.**
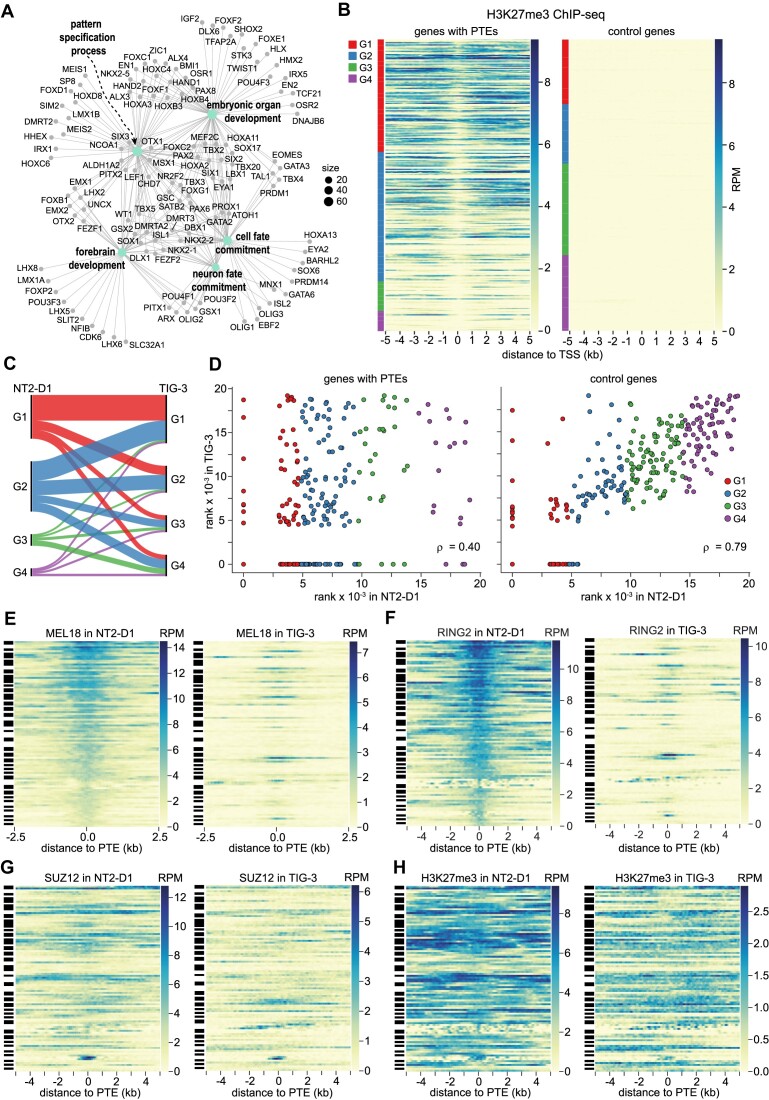
PTE-regulated genes and PRC1 tethering dynamics. (**A**) Genes regulated by the PTEs in the NT2-D1 cells grouped in a network by overrepresented GO terms. (**B**) ChIP-seq signals for H3K27me3 around TSSs of PTE-equipped and control genes ranked by their transcriptional activity (the least transcribed at the top, the most transcribed at the bottom). The color code at the left indicates the corresponding transcriptional quartiles. (**C**) Sankey diagram of transcriptional changes of the putative PTE-regulated genes between NT2-D1 and TIG-3 cells. (**D**) Scatter plots of transcriptional activity ranks for putative PTE-regulated genes and control genes that do not bind any Polycomb complexes in NT2-D1 and TIG-3 cells. Heat-map representations of MEL18 (**E**), RING2 (**F**), SUZ12 (**G**) and H3K27me3 (**H**) ChIP-seq signal around PTEs of genes that remain transcriptionally inactive (belong to G1) in both NT2-D1 and TIG-3 cells. Streak-codes to the left of heat maps indicate PTEs belonging to the same locus. Note loci bound by Polycomb complexes and H3K27me3 in NT2-D1 and devoid of either in TIG-3 cells.

The putative high-confidence PTEs are shared between 277 genes. Conversely, 29% of these genes are associated with more than one (from 2 to 5) of them. The majority of the genes have the closest PTE at a distance from 0.5 to 7 kb (Supplementary Figure S16). Analysis of the gene ontology terms associated with the PTE-equipped genes indicates that many of them are regulators of cell fate commitment, pattern specification, embryonic organ development, neuron fate commitment, and forebrain development (Figure 9A). Many of these genes encode for transcription factors and 39% are linked to heritable human syndromes (Supplementary Table S7). Using the same algorithm, we identified 125 genes regulated by putative high-confidence PTEs in TIG-3 cells. Our ChIP-seq analyses identified fewer regions enriched by MEL18 ChIP in these cells, which explains the smaller number of putative high-confidence PTEs and target genes. 62.4% of these genes are common between the two cell lines. Despite only partial overlap, the PTE-associated genes from TIG-3 cells are also enriched for regulators of cell fate commitment, pattern specification, and embryonic organ development (Supplementary Figure S17). In addition, they include regulators of skeletal and muscle tissue development, not overrepresented in the NT2-D1 gene set, which may reflect the fibroblast origin of TIG-3 cells (83). When compared to published RNA-seq data (67), 82% of the PTE-equipped genes from NT2-D1 cells have no or little transcriptional activity (ranked below median transcription level, hereafter referred to as genes of transcription groups G1-G2). 10% of the genes display moderate transcription (genes with RNA-seq signal within the third quartile, transcription group G3) and only 7%, exemplified by the *CCND2* and *ZIC2* genes, are highly transcriptionally active (genes with RNA-seq signal from the upper quartile, transcription group G4). As expected, most of these genes have chromatin around TSS decorated with H3K27me3. The extent of H3K27 methylation inversely correlates with transcriptional activity and it appears to be progressively lost with increased transcription starting from regions immediately around TSSs (Figure 9B). The latter may reflect the increased nucleosome exchange that accompanies transcription initiation or the necessity to remove H3K27me3 at the TSS in order to commence transcription.

Judging from published RNA-seq data (67,68), PTE-equipped genes with low transcriptional activity in NT2-D1 tend to stay in that state also in TIG-3 cells. However, some genes change to moderate or highly transcribed (Figure 9C). Conversely, some of the genes highly transcribed in NT2-D1 cells (e.g. *CCND2* and *ZIC2*) become transcriptionally inactive in TIG-3 (Figure 9C). We note that the levels of transcriptional activity in NT2-D1 and TIG-3 cells for genes not regulated by the Polycomb system are well correlated (ρ = 0.79; Figure 9D). In contrast, the transcriptional output from PTE-equipped genes is much less consistent (ρ = 0.4; Figure 9D) suggesting that these genes are predisposed to change their transcription more discretely. Epigenetic regulation by the Polycomb system likely contributes to this property.

Transcriptional activity is known to antagonize PRC2 binding and H3K27 methylation by means that are not fully understood (43,84,85) and even PRC1 is sometimes lost from transcriptionally active *Drosophila* genes (5). However, factors other than those linked to transcription modulate the binding of PRC1 in human cells. Side-by-side comparison of PRC1 binding to genes that are transcribed at very low levels (belong to group G1) in both NT2-D1 and TIG-3 cells indicates that, in the latter, many PTEs are no longer immunoprecipitated with either anti-MEL18 or anti-RING2 antibodies (Figure 9E, F). For some genes with several PTEs, MEL18 and RING2 signals are lost from one PTE but remain above genomic average at other PTEs. Yet, in 42% of the cases, all PTEs of a gene that were occupied by MEL18 in NT2-D1 cells, display no immunoprecipitation with antibodies against MEL18 in TIG-3 cells. In one third of these genes, all PTEs in TIG-3 cells also lack detectable RING2 ChIP-seq signal. The ChIP-seq signals for MEL18 and RING2 in TIG-3 cells are generally lower compared to those in NT2-D1 cells. This is consistent with lower transcription of *MEL18* and *RING2* genes (54) and may indicate a generally lower abundance of PRC1 in TIG-3 cells. However, this does not fully account for the PRC1 loss because some of the PTEs that display very high MEL18 and RING2 ChIP-seq scores in NT2-D1 cells are no longer immunoprecipitated in TIG-3 cells, while some of those with weaker scores remain occupied in both cell lines. Consistent with the gene sets being transcriptionally inactive in both cell lines, most of the genes retain a significant presence of SUZ12 and H3K27me3 in TIG-3 cells (Figure 9G, H).

Overall, our observations suggest that canonical PRC1 complexes do not bind PTEs of transcriptionally inactive target genes by default and additional processes ‘license’ PTEs for binding. The extensive catalogue of human PTEs presented here provides a major new resource to discover these processes.

## Discussion

Three central conclusions follow from the study presented here. First, many human developmental genes contain DNA elements necessary and sufficient for tethering canonical PRC1. Second, the binding of PRC1 and PRC2 to a regulated locus is not strictly linked and the presence of PRC2-catalyzed H3K27me3 is not enough for efficient PRC1 tethering. Third, the DNA features associated with PRC1 tethering differ from those that favour the tethering of PRC2. Throughout the genome, the two kinds of sequence features combine in different proportions to yield a range of DNA elements that vary from those tethering predominantly either PRC1 or PRC2 to ones that can tether both complexes.

The discovery of hundreds of PRC1 Tethering Elements puts at rest the question of whether DNA elements play a role in directing canonical PRC1 to human genes (86,87). The emerging picture is similar to the paradigmatic targeting of Polycomb complexes by Polycomb Response Elements (PREs) of *Drosophila* but with instructive differences. Both organisms appear capable of tethering PRC2 to specific sites independently of PRC1 (88). Yet, in *Drosophila*, the high-occupancy sites for the two complexes always coincide at PREs while, in human cells, PRC1 and PRC2 often prefer to bind different parts of the gene.

The distinction may stem from dissimilar primary structures of human and *Drosophila* genomes. Shaped by abundant CpG DNA methylation, the human genome is generally CpG-poor save for complex regulatory regions of tissue and cell-type specific genes (89,90). These genes appear ‘automatically’ marked for preferential binding by PRC2, which has a propensity to bind CpG-rich DNA (51,73). When equipped with one or more PRC1 tethering elements (PTEs), such a gene is set for regulation by the Polycomb system. *Drosophila melanogaster* has lost the CpG methylation (91) and, with it, the ability to distinguish the tissue and cell-type specific genes from the rest of the genome by their CpG-content. We speculate that flies had to evolve tighter coordination in the tethering of PRC2 and PRC1 (88,92) to compensate for this loss.

Most of what we know about the genomic binding of mammalian PRC1 and PRC2 is derived from studies of mouse embryonic stem cells. There PRC1 and PRC2 bind repressed genes broadly with no sites standing out as being highly occupied by PRC1 (37,42,46). The findings presented here paint a different picture. While we cannot exclude that distinct ChIP-seq profiles of human PRCs are a feature of this species, we think it is unlikely. For example, ChIP-qPCR mapping of PRC1 and PRC2 across the murine *Ccnd2* gene in NIH-3T3 mouse embryonic fibroblasts revealed distinct PRC1 peak orthologous to human *CCND2* PTE (54). Moreover, the DNA underneath this peak generates a new PRC1 binding site when introduced into the human genome (54).

Some of the apparent differences may stem from the adjustment of the ChIP-seq procedure where we omitted the size-selection step and, instead, fragmented immunoprecipitated DNA enzymatically before the ligation of adapters for Illumina sequencing. In our hands, this makes immunoprecipitation profiles obtained by the next-generation sequencing more comparable to those derived from the same ChIP reaction by qPCR. Yet, we suspect that the major source of the discrepancy is inherent to mouse embryonic stem cells and comes from their unusually high levels of PRC2 (23,93) and PRC1. A systematic comparison of PRC1 and PRC2 abundance to their binding patterns will help to clarify this issue. Regardless, our observations suggest that the behaviour of the Polycomb system in mouse embryonic stem cells is not representative of all mammalian cell types.

Most contemporary models of the mammalian Polycomb system assume that canonical PRC1 binds to repressed genes via the interaction of its CBX subunit with H3K27me3. This assumption does not easily fit the observations presented here. In the NT2-D1, TIG-3 and F10 cells, the ChIP-seq profiles of canonical PRC1 and PRC2 differ and the presence of PRC2-catalyzed H3K27me3 is not sufficient to achieve PRC1 occupancy comparable to that at PTEs. It is worth to note that, in *Drosophila*, for which the H3K27me3 - CBX hierarchy was first proposed (94), PRC1 remains bound to PREs in cells depleted of PRC2 and H3K27 methylation (88). To summarize, even though H3K27 methylation is essential for repression (19,20), its mechanistic contribution remains to be fully understood.

The overrepresentation of oriented poly(dA) tracts within PTEs suggests that PRC1 tethering is somehow linked to chromatin remodeling by SWI/SNF (i.e. BAF or PBAF) complexes. In line with this notion, Weber and co-authors have found that rapid degradation of BRG1, the core ATPase subunit of BAF and PBAF complexes, leads to substantial loss of PRC1 from the most highly occupied binding sites in mouse embryonic stem cells (95). It would be interesting to investigate whether the same holds true for human cells and, if so, which of the two complexes, BAF or PBAF, is implicated. Consistent with nucleosome remodeling, regions underneath MEL18 peaks are sensitive to MNase digestion. Our analyses likely underestimate this sensitivity because only a fraction of PTEs, defined from PRC1 binding in NT2-D1 cells, are occupied by the complex in K562 cells, used for MNase titration experiments (66). The necessity of chromatin remodeling would explain why canonical PRC1 complexes do not always bind PTEs of transcriptionally inactive genes.

The work presented here advances our understanding of how human PRC1 binds to specific genes. It also raises several new questions. Do PTEs vary in their ability to tether PRC1 complexes composed of different CBX or PHC paralogues? How do deletions of individual PTEs affect the expression of their cognate genes in the context of the whole organism? What fraction of PTE-equipped genes linked to heritable human syndromes has alleles that carry PTE deletions or duplications? Our catalogue of human PTEs provides a valuable new resource to address these questions. Equally significant, it presents an opportunity to design transgenic assays to disentangle individual contributions of PRC1 and PRC2 to the repression by the Polycomb system and to screen for factors that enable PRC1 tethering.

## Supplementary Material

gkad889_Supplemental_File

## Data Availability

The data underlying this article are available in the Gene Expression Omnibus at https://www.ncbi.nlm.nih.gov/geo/, and can be accessed under GSE207401.
