# Supplementary material for: DNA elements tether canonical Polycomb Repressive Complex 1 to human genes
Source: Nucleic Acids Res. 2023 Oct 19;51(21):11613–33. doi: 10.1093/nar/gkad889 (PMC10681801; doi:10.1093/nar/gkad889)
Supplement: gkad889_Supplemental_File [file gkad889_supplemental_file.pdf]

DNA elements tether canonical Polycomb Repressive Complex 1 to human genes

Juan I. Barrasa*, Tatyana G. Kahn*, Moa J. Lundkvist* and Yuri B. Schwartz

Department of Molecular Biology, Umeå University,

901 87, Umeå, Sweden

* equal contribution

correspondence should be addressed to YBS: yuri.schwartz@umu.se

Supplementary materials

Supplementary text

Supplementary figures S1-S17

Supplementary tables S1-S7

Supplementary text

In ChIP-seq library preparation protocols with the size-selection step, all immunoprecipitated DNA fragments larger than 200 bp are discarded. This may lead to the underrepresentation of certain genomic regions in the sequencing libraries and distortions of enrichment profiles from the ChIP-sequencing assay compared to those before the library preparations. In the figure below, we illustrate these effects for MEL18 ChIP and two representative loci: *CCND2* (panel **A**) and *SP8* (panel **B**). Two MEL18 ChIP samples were analysed by qPCR, which gave very similar results. Subsequently, one MEL18 ChIP sample was used to prepare the ChIP-seq library with the size selection step; in that case, all fragments larger than 200 bp were discarded. For the second MEL18 ChIP sample, immunoprecipitated DNA was enzymatically fragmented before library preparation, resulting in the average fragment size of 180 bp (ChIP DNA was treated with NEBNext Ultra II FS Enzyme Mix for 20 minutes at 37°C, as described in NEBNext® Ultra™ II FS DNA Library Prep Kit for Illumina, #E7805). In this case, most ChIP DNA fragments were represented in the resulting ChIP-seq library. Relative signal intensities for different qPCR amplicons match those at corresponding genomic positions of ChIP-seq profiles from libraries made without size-selection, while there is a notable deviation between the two in the case of standard "size-selection" protocol.

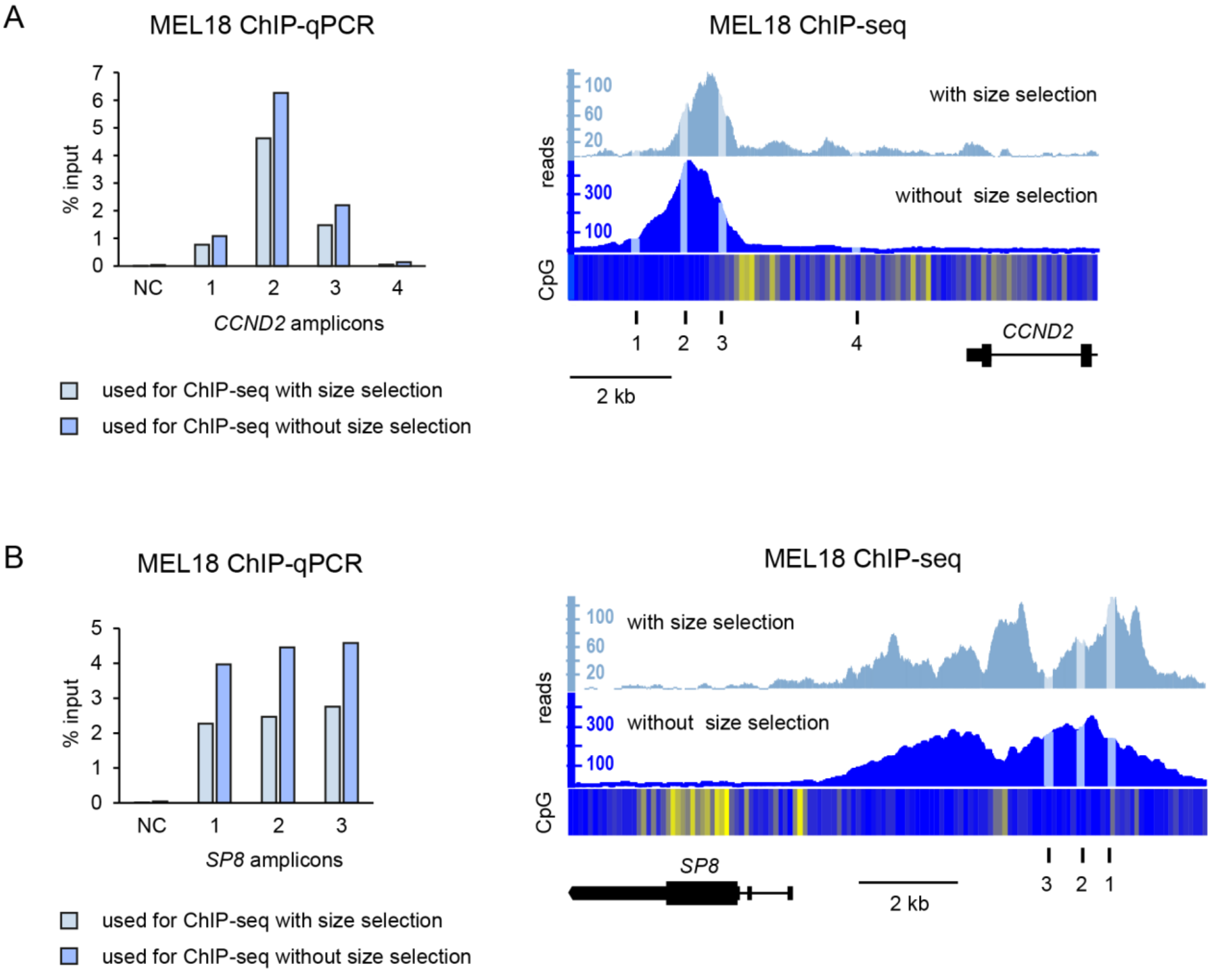

Comparison of MEL18 ChIP-seq profiles obtained with or without size-selection during ChIP-seq library preparation. The MEL18 signal from the ChIP-seq library prepared with size selection is depicted in light blue, while the MEL18 signal from the ChIP-seq library prepared without size selection is shown in dark blue. The heat-map underneath ChIP-seq profiles shows the number of CpG nucleotides within the 100bp sliding window (ranging from dark blue=0 to bright yellow=15). The numbers below the heat-map indicate the position of qPCR amplicons used for ChIP-qPCR.

Supplementary figures

Figure S1

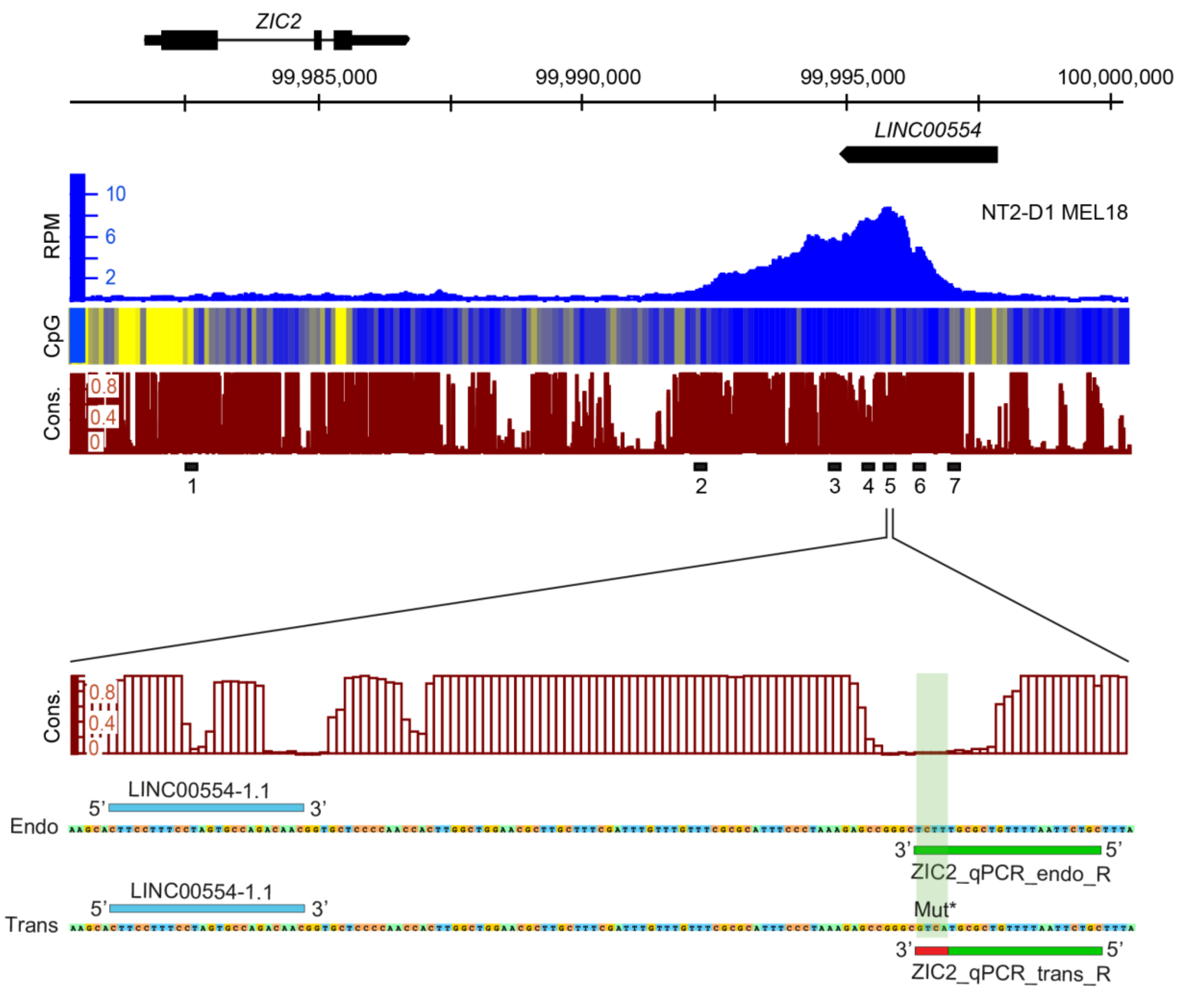

Figure S1. Design of the *ZIC2* transgene-specific PCR probe. To discriminate between the endogenous and the transgenic copy of the putative PRC1 tethering element, we used the *phastCons 100-way* conservation score to identify a small stretch of nucleotides right under the summit of the MEL18 binding peak in the *ZIC2* locus that showed little conservation within mammalian species. We posited that these non-evolutionary conserved nucleotides do not contain any important regulatory sequences and substituted them in the transgenic copy to create an annealing site for a transgene-specific PCR primer. Both the endogenous and transgenic amplicons share the same forward primer (LINC00554-1.1), but use different reverse primers (ZIC2_qPCR_endo_R and ZIC2_qPCR_trans_R respectively).

Figure S2

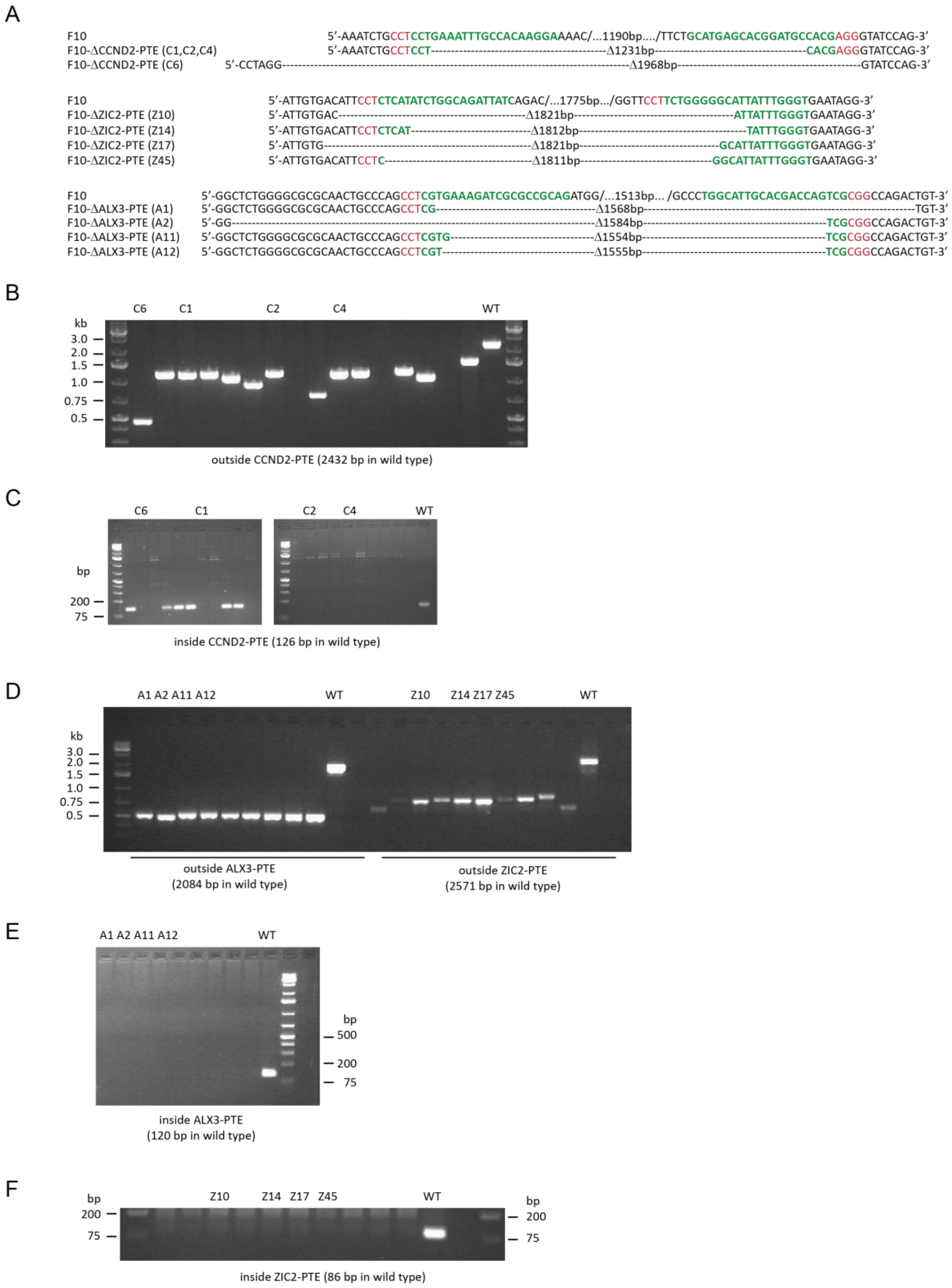

Figure S2. Genotyping of F10 cell lines with *CCND2*, *ZIC2,* and *ALX3* PTE deletions. A. Nucleotide sequences of new deletion alleles used in the study. Sequences of sgRNA targets

are marked in green, PAM sequences are marked in red. **B.** PCR genotyping of F10 clonal derivatives after CRISPR/Cas9-mediated genome editing around *CCND2* PTE using primers flanking the expected PTE deletion. **C.** The same analysis using primers inside the expected PTE deletion. **D.** PCR genotyping of F10 clonal derivatives after CRISPR/Cas9-mediated genome editing around *ALX3* and *ZIC2* PTEs using primers flanking the expected PTE deletions. The same analysis using primers inside the expected *ALX3* (**E**) and *ZIC2* (**F**) PTE deletions. Agarose gel lanes with products from PCRs with genomic DNA of cell lines used for further analyses are marked with corresponding clone names. Names and nucleotide sequences of the primers and sgRNAs are listed in Table S2.

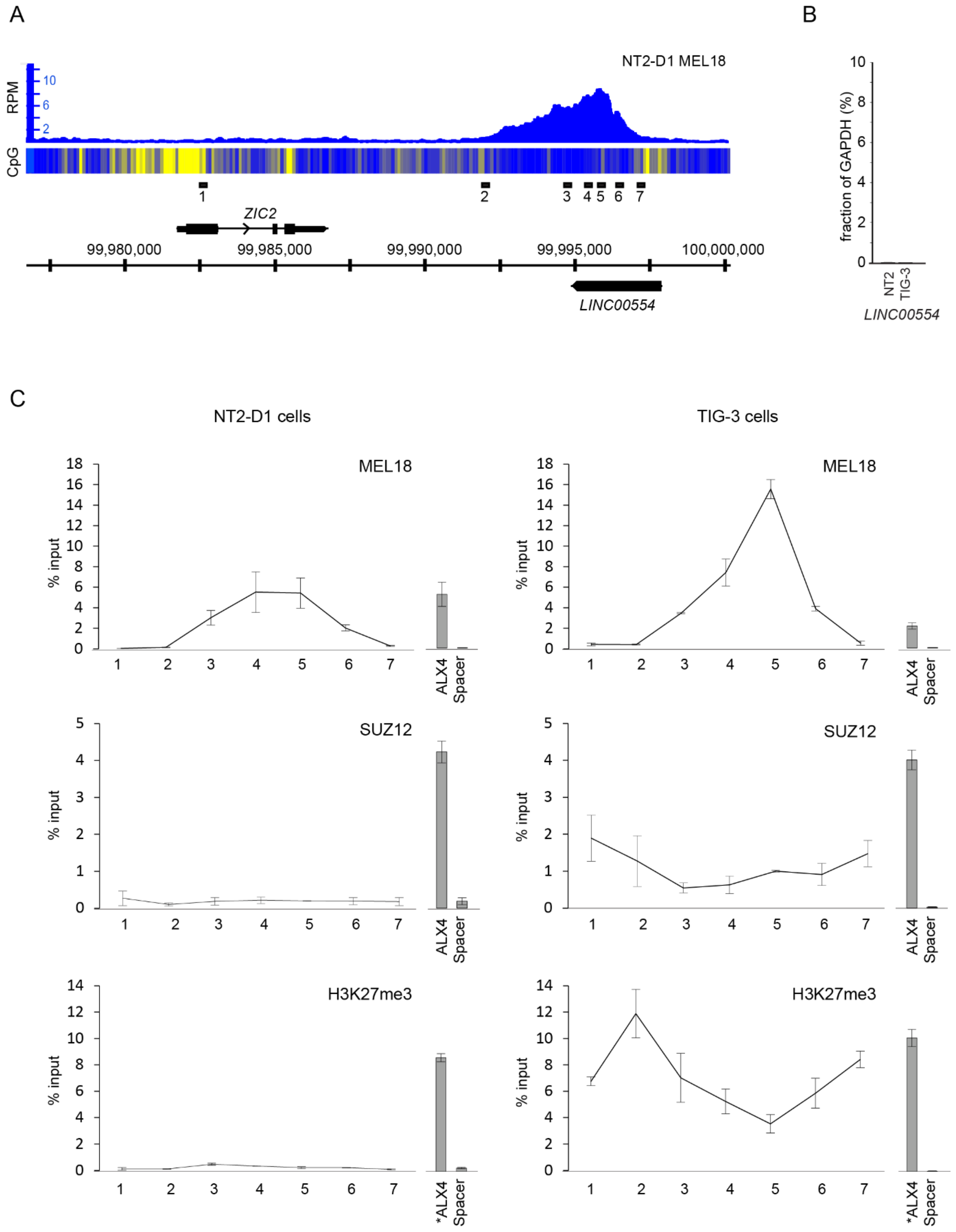

Figure S3. Detailed characterization of human *ZIC2* locus. A. ChIP-seq with chromatin from NT2-D1 cells suggests strong binding of MEL18 downstream of the *ZIC2* gene. The heat-map underneath ChIP-seq profiles shows the number of CpG nucleotides within the 100bp sliding window (ranging from dark blue=0 to bright yellow=15). The *ZIC2* gene is transcribed from left to right and the long non-coding RNA *LINC00554* of unknown function

is transcribed from right to left. Positions of PCR amplicons (black rectangles) analyzed in **C** are shown above the scale in GRCh38/hg38 genomic coordinates. **B.** RT-qPCR measurements indicate that the long non-coding RNA *LINC00554* is not produced in either NT2-D1 (NT2) or TIG-3 cells. Values are normalized to the expression of the housekeeping *GAPDH* gene. **C.** ChIP-qPCR profiles of MEL18, SUZ12, and H3K27me3 in NT2-D1 (left column) and TIG-3 (right column) cells. The immunoprecipitation of *ALX4* gene repressed by Polycomb mechanisms in NT2-D1 and TIG-3 cells was used as positive control and Chromosome 12 "spacer" region was assayed as negative control. All histograms and graphs show the average and the scatter (whiskers) between two independent experiments. Note that in TIG-3 cells H3K27me3 and SUZ12 profiles are offset from the MEL18 peak into neighboring CpG-rich regions.

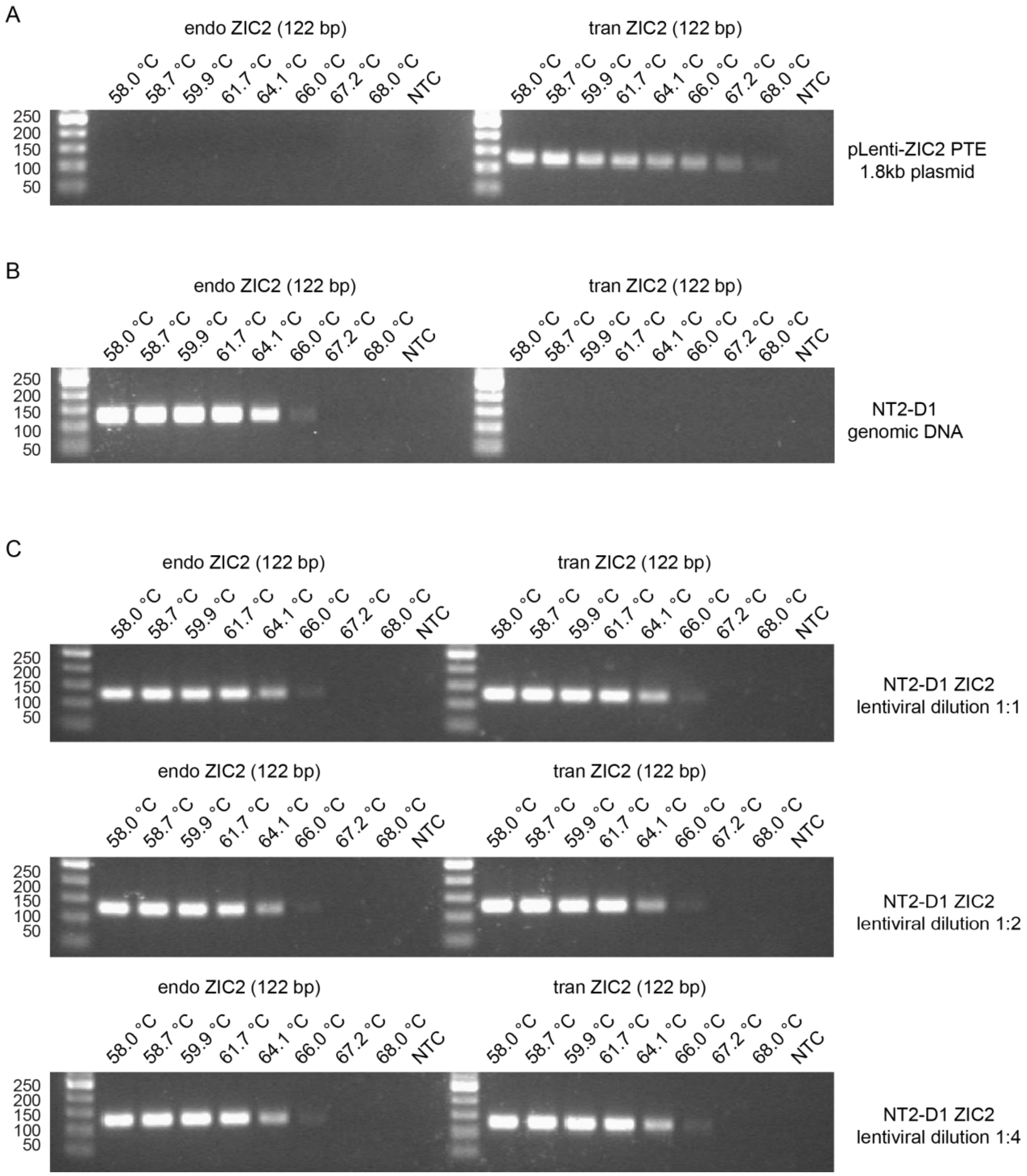

Figure S4. PCR genotyping of NT2-D1 cells transduced with the *ZIC2* PRC1 tethering element construct. The specificity of selected PCR primers for amplification of either endogenous or transgenic copies of the PRC1 tethering element was tested by gradient PCR using pLenti-ZIC2 PTE 1.8-kb plasmid DNA **(A)** or NT2-D1 genomic DNA **(B)** as a template. The results indicate that the PCR with transgene-specific primers amplifies the product of expected size from pLenti-ZIC2 PTE 1.8-kb plasmid but not from genomic DNA. Conversely, the PCR with primer pair specific for endogenous region amplifies the expected product from NT2-D1 genomic DNA but not from pLenti-ZIC2 PTE 1.8-kb plasmid. **C.** The two sets of primers were used to genotype NT2-D1 cells transduced with the lentiviral *ZIC2*

PTE construct. The genotyping confirms that cells contain the expected transgene. Sequences of corresponding amplicons and primers are indicated in Table S5.

Figure S5

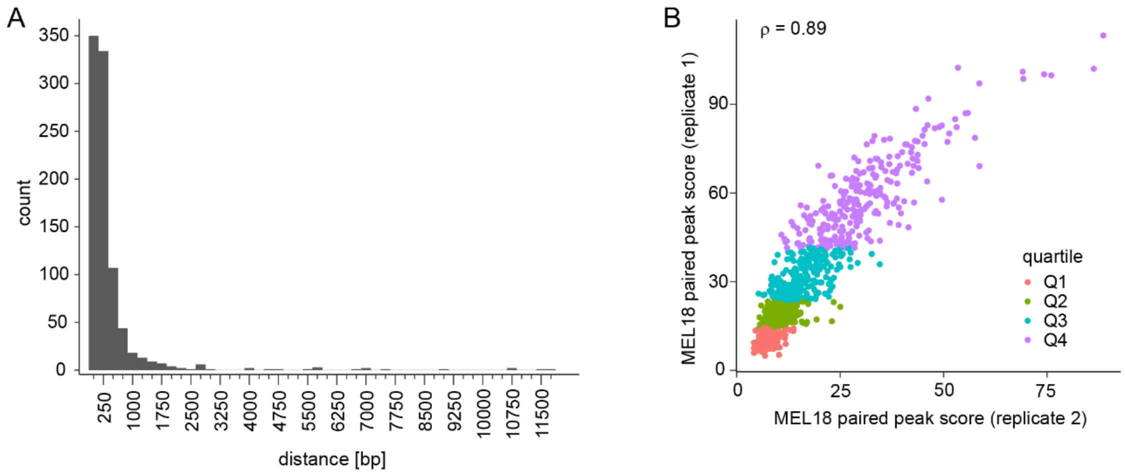

Figure S5. Identification of distinct MEL18 ChIP-seq peaks. A. Histogram of distances between the closest MEL18 ChIP-seq signal peaks from replicate experiments. **B.** The scatter plot of ChIP-seq signal scores for the paired MEL18 peaks indicates that these are highly correlated and, most often, represent the same binding site.

Figure S6

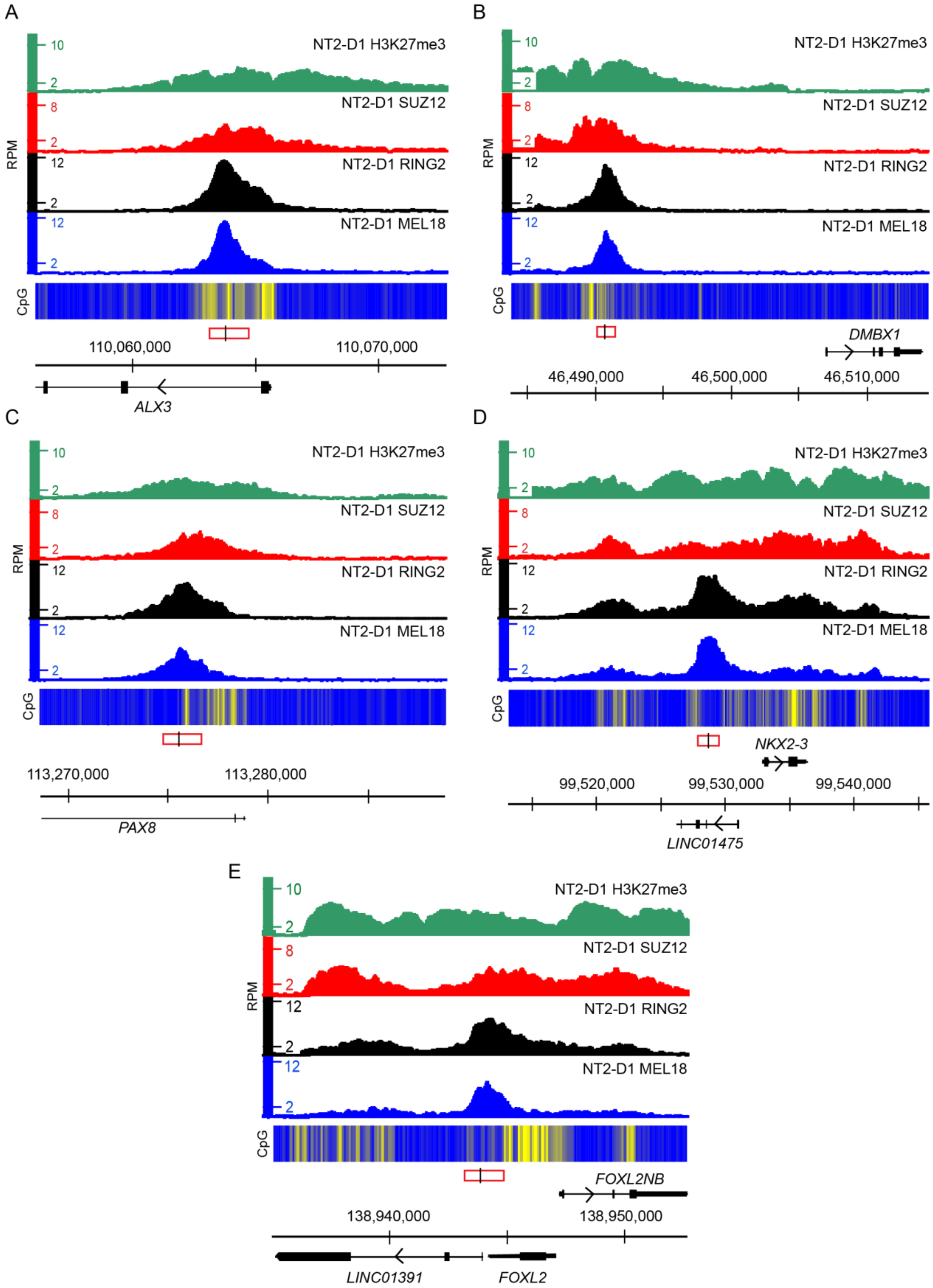

Figure S6. Binding of Polycomb group proteins around selected discrete Q4 MEL18 peaks. H3K27me3, SUZ12, RING2 and MEL18 ChIP-seq profiles over *ALX3* (**A**), *DMBX1* (**B**), *PAX8* (**C**), *NKX2*-3 (**D**) and *FOXL2* (**E**) loci. The heat-map underneath ChIP-seq profiles

shows the number of CpG nucleotides within the 100bp sliding window (ranging from dark blue=0 to bright yellow=15). The regions used for transgenic assays are marked by red rectangles, with a black line indicating the position of the transgene-specific amplicon. Transcripts present in these loci are shown along the scale in GRCh38/hg38 genomic coordinates.

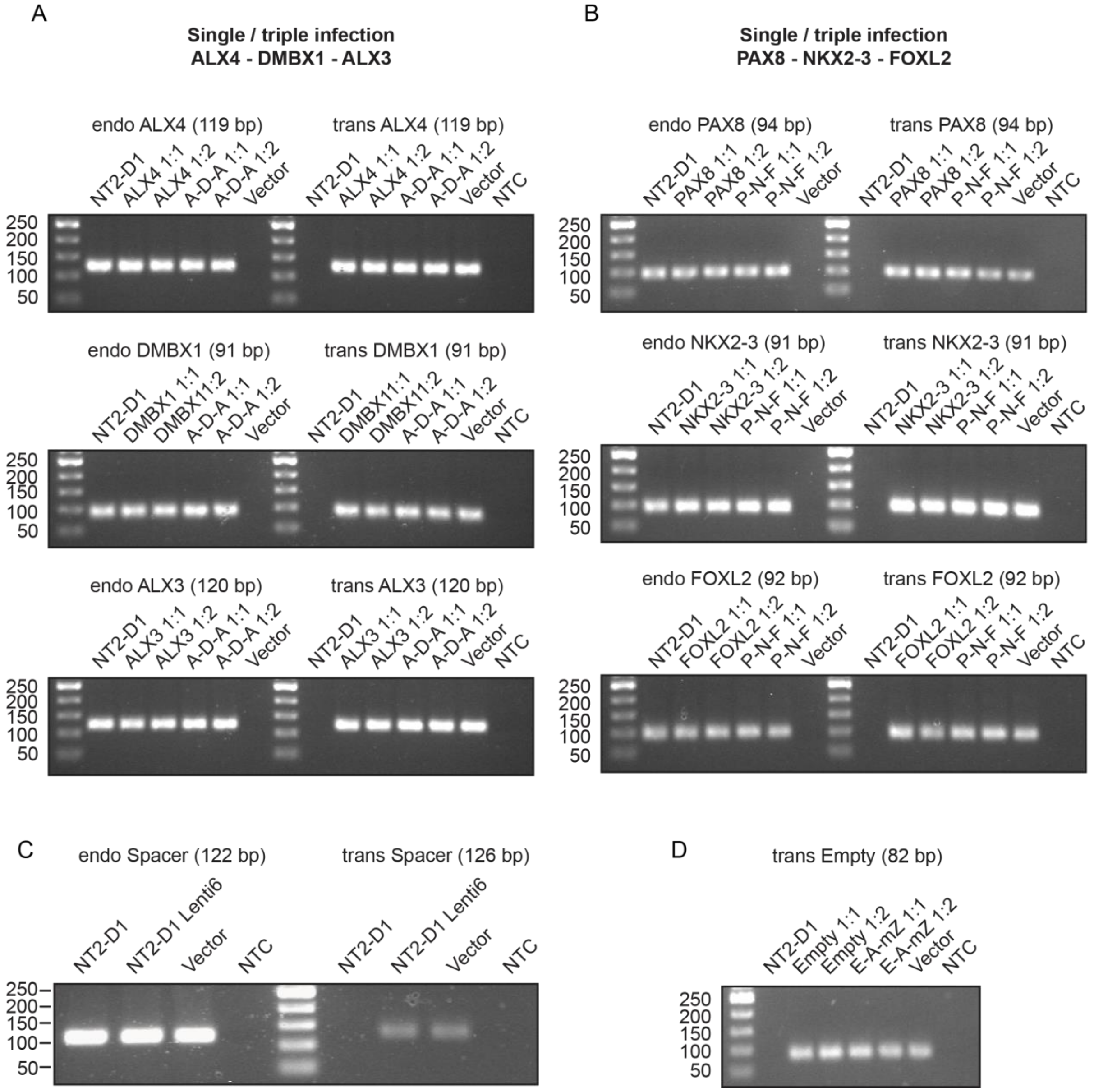

Figure S7. PCR genotyping of NT2-D1 cell lines transduced with Q4 PTEs. NT2-D1 cells were transduced with either a single or a pool of three different lentiviral constructs, using undiluted (1:1) or 1:2 diluted lentiviral supernatants. For all tested amplicons, NT2-D1 genomic DNA from uninfected cells was used as a positive control for the endogenous amplicon (endo) and as a negative control for the transgenic amplicon (trans). The correspondent lentiviral vectors were used as positive controls for the transgenic amplicons and as negative controls for the endogenous amplicons. The size of each amplicon is indicated in brackets. **A.** PCR genotyping of NT2-D1 cells transduced individually with *ALX4*, *DMBX1,* or *ALX3* PTEs transgenic copies, or with the three of them together (A-D-A). **B.** PCR genotyping of NT2-D1 cells transduced individually with *PAX8*, *NKX2-3,* or *FOXL2* PTEs transgenic copies, or with the three of them together (P-N-F). In both (**A**) and (**B**) the genotyping confirms the specificity of the endogenous and transgenic amplicons and that cells transduced with individual and multiple lentiviral constructs contain the expected

transgenes. **C.** PCR genotyping of NT2-D1 cells transduced with a lentiviral construct containing spacer DNA (NT2-D1 Lenti6). **D.** PCR genotyping of NT2-D1 cells transduced with either a single lentiviral construct containing empty vector (Empty) or a pool of three different constructs containing empty vector, *ARID3C* PTE and *ZIC2_mutTCG* PTE (E-A-mZ). Sequences of corresponding amplicons and primers are indicated in Table S5.

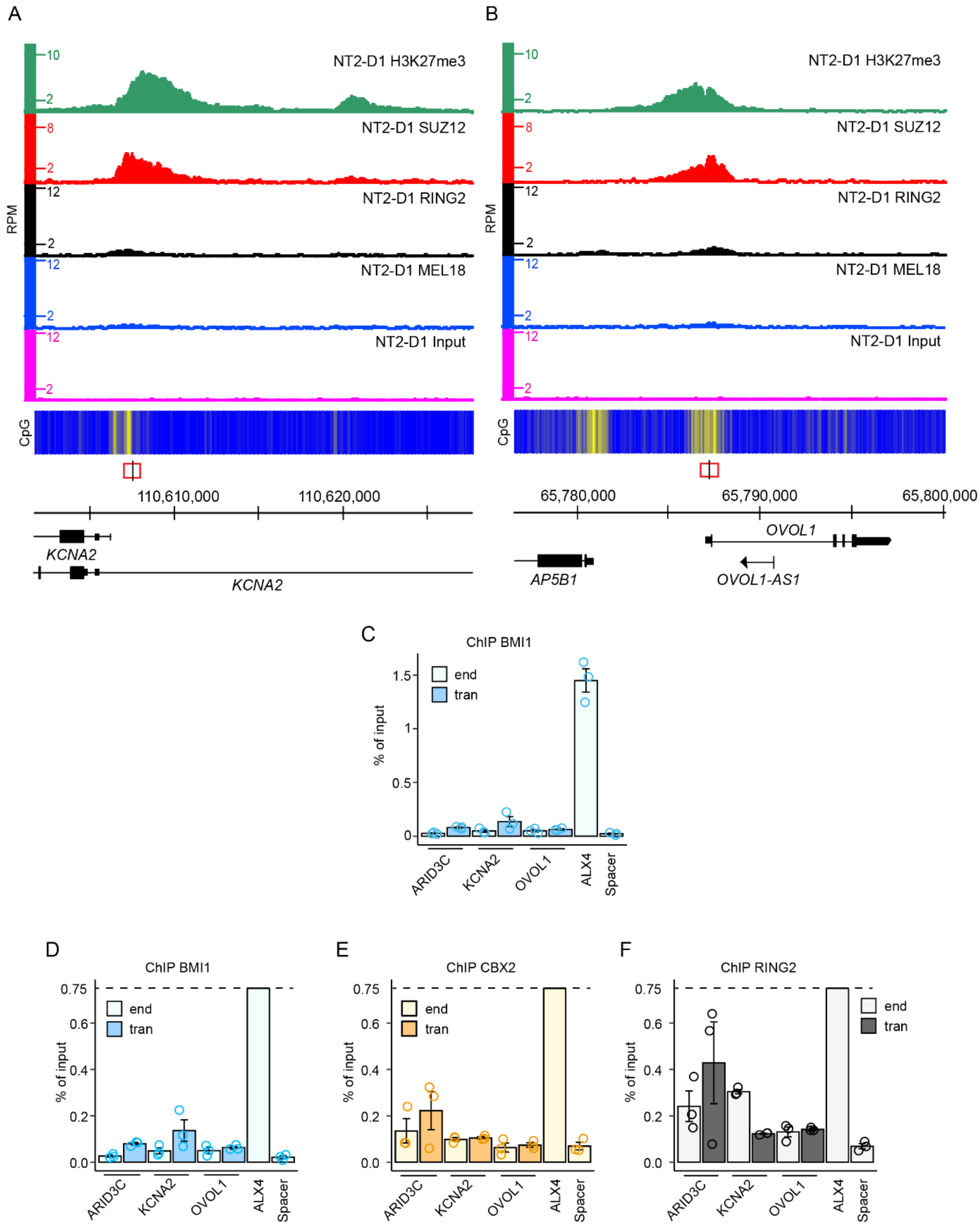

Figure S8. The binding of PRC2 and PRC1 to transgenic MEL18-free PRC2-bound regions. Screenshots of the MEL18, RING2, SUZ12, H3K27me3, and Input ChIP-seq profiles around *KCNA2* (**A**) and *OVOL1* (**B**) genes in NT2-D1 cells. The fragments used for transgenic experiments are indicated by red rectangles, with the genomic position of ChIP-qPCR amplicons marked by a vertical black line. **C.** ChIP-qPCR experiments using BMI1 antibody as an additional canonical PRC1 component and confirming the results with CBX2

and RING2 antibodies are shown in Figure 4F-G. Scaled bar-plots of yields from ChIPs with antibodies against BMI1 (**D**), CBX2 (**E**), and RING2 (**F**). Bar-plots are trimmed at the y-axis value of 0.75% (indicated with a dashed line).

Figure S9

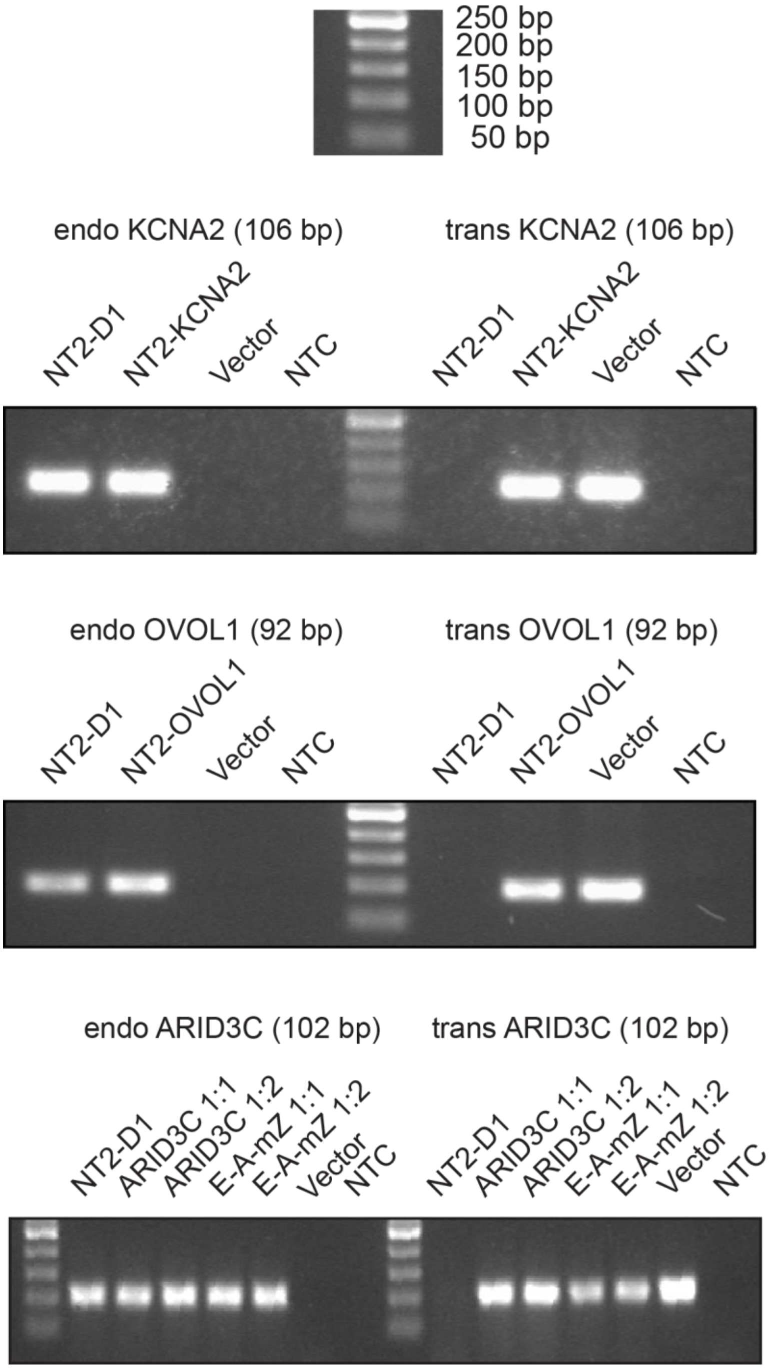

Figure S9. PCR genotyping of cells transduced with MEL18-free PRC2-bound regions. NT2-D1 cells were transduced with the single lentiviral construct containing *KCNA2* PTE or the single lentiviral construct containing *OVOL1* PTE or the single lentiviral construct containing *ARID3C* PTE, or the pool of three different lentiviral constructs containing empty vector, *ARID3C* PTE and *ZIC2_mutTCG* PTE (E-A-mZ). Undiluted (1:1) or two-fold diluted (1:2) lentiviral supernatants we used for some of the infections. For all tested amplicons, NT2-D1 genomic DNA from uninfected cells was used as a positive control for the endogenous amplicon (endo) and as a negative control for the transgenic amplicon (trans). The correspondent lentiviral vectors were used as positive controls for the transgenic

amplicons and as negative controls for the endogenous amplicons. The size of each amplicon is indicated in brackets.

Figure S10

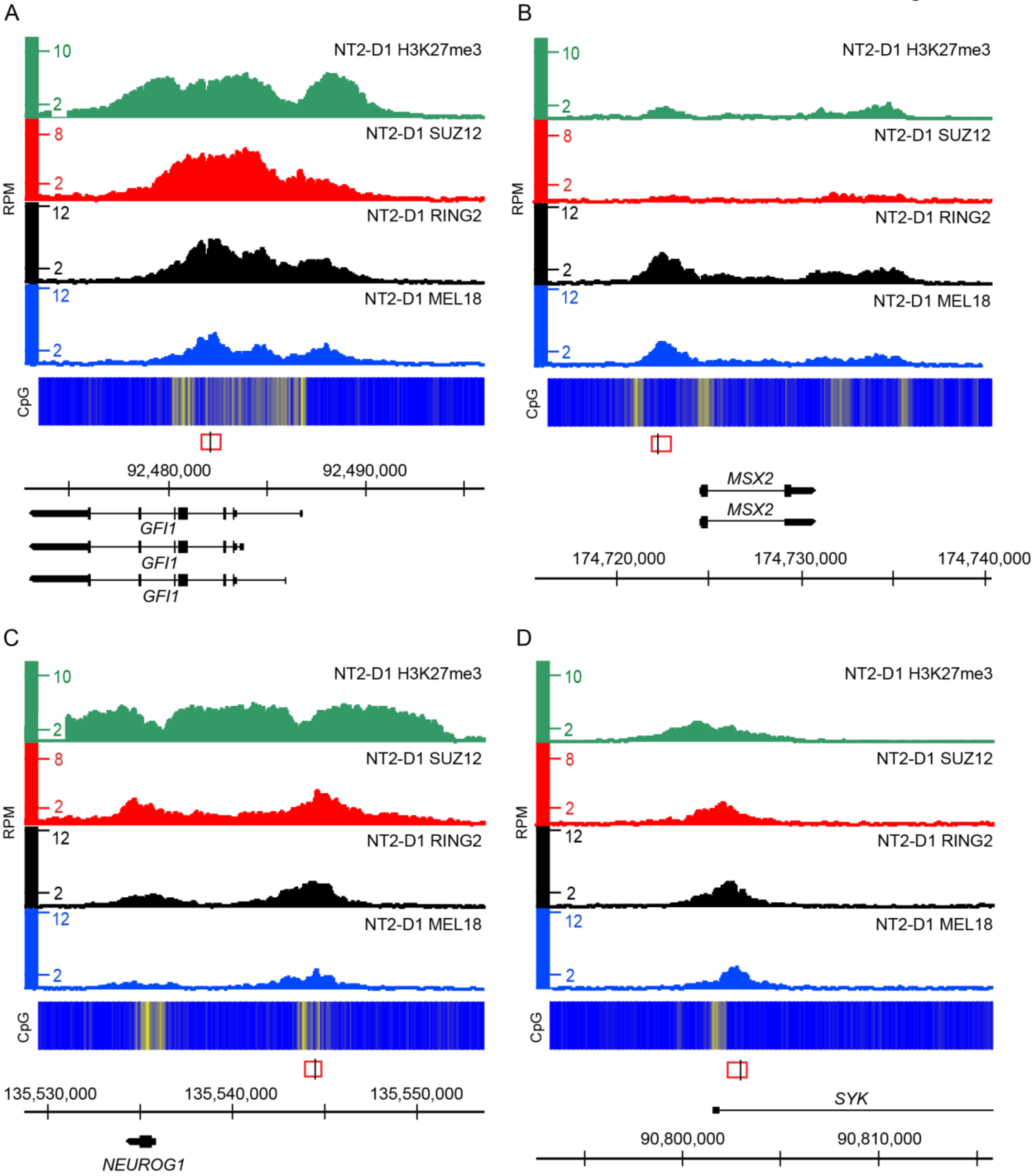

Figure S10. Binding of Polycomb group proteins around selected discrete Q3-Q2 MEL18 peaks. H3K27me3, SUZ12, RING2, and MEL18 ChIP-seq profiles over *GFI1* (**A**), *MSX2* (**B**), *NEUROG1* (**C**), and *SYK* (**D**) loci. The heat-map underneath ChIP-seq profiles shows the number of CpG nucleotides within the 100bp sliding window (ranging from dark blue=0 to bright yellow=15). The regions used for transgenic assays are marked by red rectangles, with a black line indicating the position of the transgene-specific amplicon. Transcripts present in these loci are shown along the scale in GRCh38/hg38 genomic coordinates.

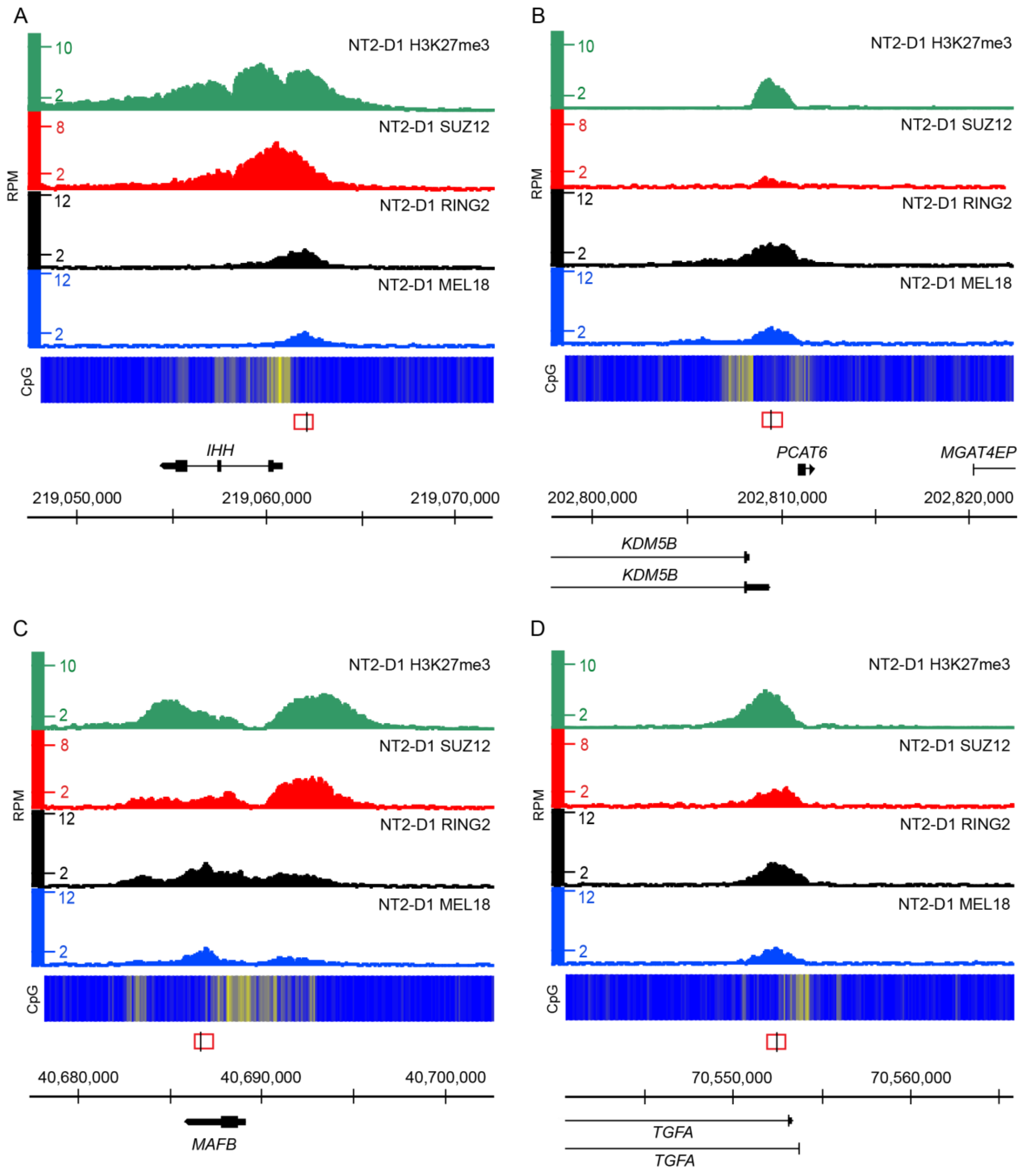

Figure S11. Binding of Polycomb group proteins around selected discrete Q3-Q2 MEL18 peaks. H3K27me3, SUZ12, RING2, and MEL18 ChIP-seq profiles over *IHH* (**A**), *KDM5B* (**B**), *MAFB* (**C**), and *TGFA* (**D**) loci. The heat-map underneath ChIP-seq profiles shows the number of CpG nucleotides within the 100bp sliding window (ranging from dark blue=0 to bright yellow=15). The regions used for transgenic assays are marked by red rectangles, with a black line indicating the position of the transgene-specific amplicon. Transcripts present in these loci are shown along the scale in GRCh38/hg38 genomic coordinates.

Figure S12

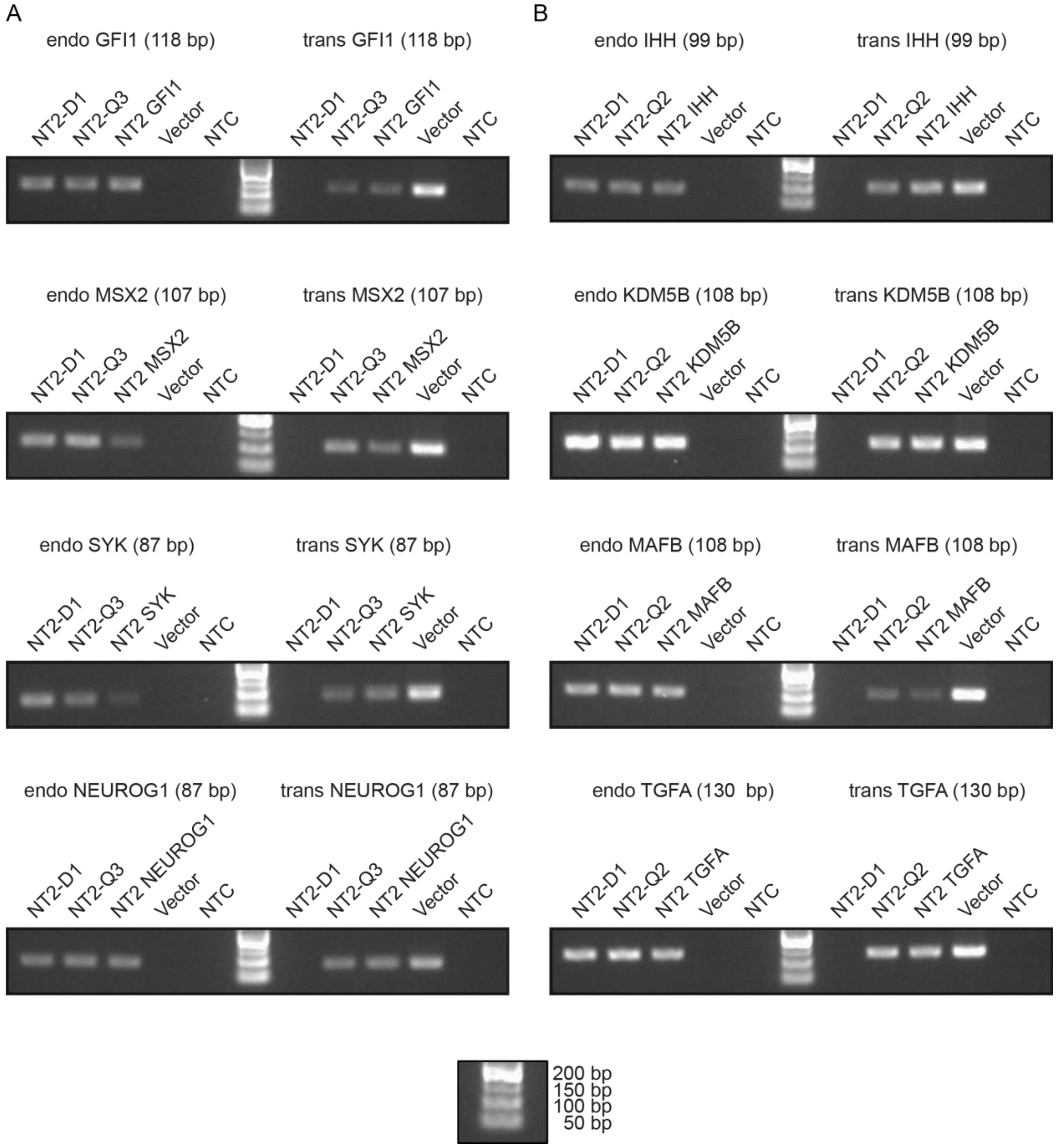

Figure S12. PCR genotyping of NT2-D1 cell lines transduced with Q3-Q2 PTE constructs. A. PCR genotyping of NT2-D1 cells transduced with *GFI1*, *MSX2*, *SYK* or *NEUROG1* PTE constructs individually and as a pool of all four constructs (NT2-Q3). **B.** PCR genotyping of NT2-D1 cells transduced with *IHH*, *KDM5B*, *MAFB* or TGFA PTE constructs individually and as a pool of all four constructs (NT2-Q2). For all tested amplicons, NT2-D1 genomic DNA from uninfected cells was used as a positive control for the endogenous amplicon (endo) and as a negative control for the transgenic amplicon (trans). The correspondent lentiviral vectors were used as positive controls for the transgenic amplicons and as negative controls for the endogenous amplicons. The size of each amplicon is indicated in brackets. In both (**A**) and (**B**) the genotyping confirms the specificity of the endogenous and transgenic amplicons and that cells transduced with the lentiviral constructs

contain the expected transgenes. Sequences of corresponding amplicons and primers are indicated in Table S5.

Figure S13

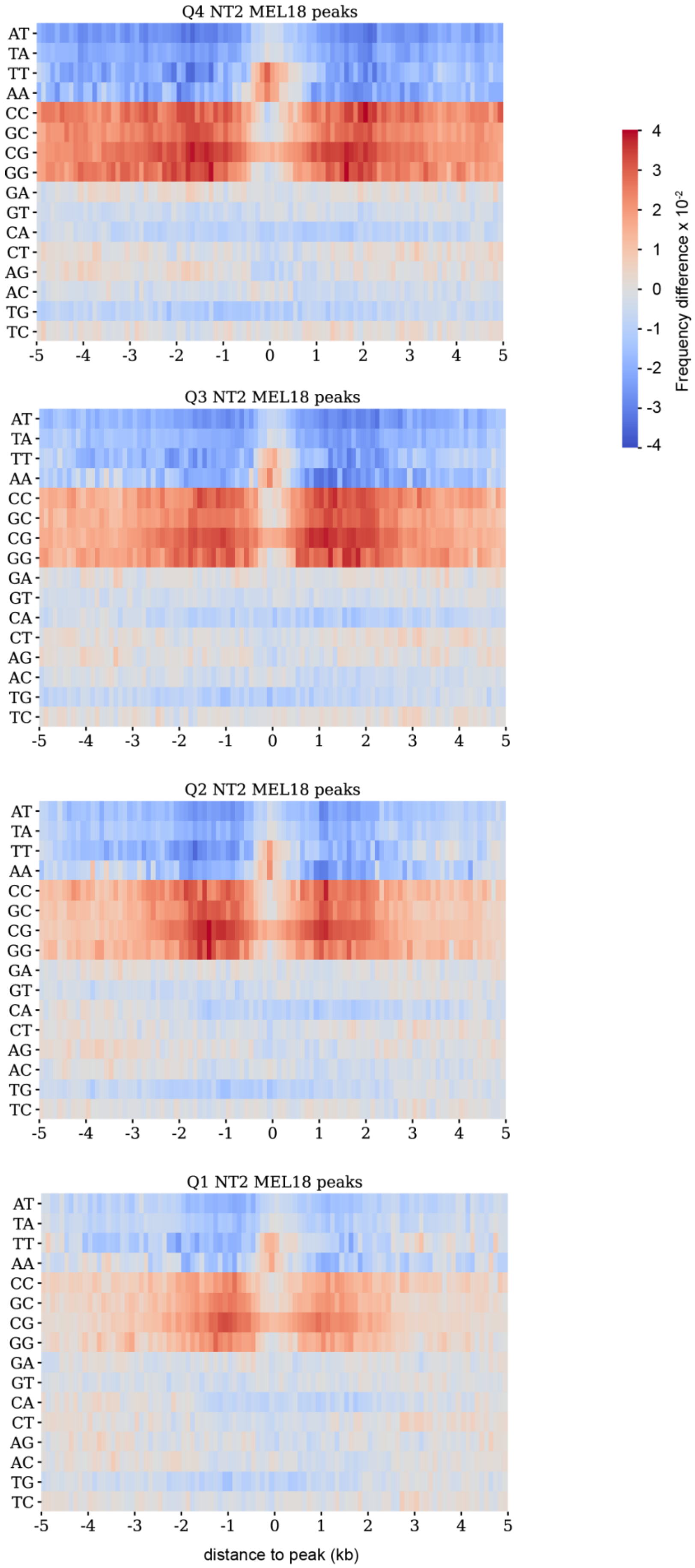
Q4 NT2 MEL18 peaks
Q3 NT2 MEL18 peaks
Q2 NT2 MEL18 peaks
Q1 NT2 MEL18 peaks
AT
TA
TT
AA
CC
GC
CG
GG
GA
GT
CA
CT
AG
AC
TG
TC
-5
-4
-3
-2
-1
0
1
2
3
4
5
distance to peak (kb)
Frequency difference x 10^{-2}

Figure S13. Di-nucleotide frequencies around discrete MEL18 peaks in NT2-D1 cells. Note that AA and TT enrichment under the peaks and the overall CG richness of the flanks become less prominent around less occupied (lower quartile) peaks.

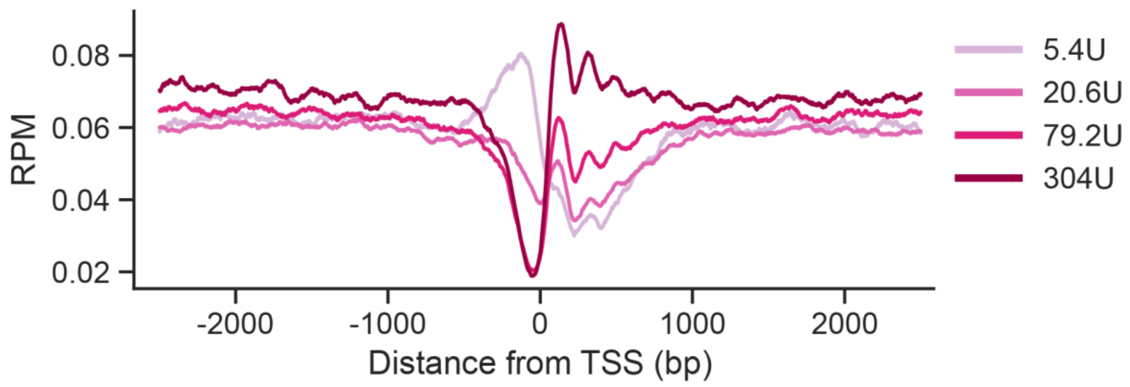

Figure S14. Mean MNase-seq profiles around Transcription Start Sites (TSS) of highly transcriptionally active genes. The MNase-seq signals are from titration assay by (Mieczkowski et al. 2016) and normalized for the sequencing depth. Note an increase of the signal in light-digested samples (treated with 5.4U of MNase) and substantial reduction of the signal upon deep digestion (samples treated with 79.2U and 304U of MNase) over the region immediately upstream of TSS.

Figure S15

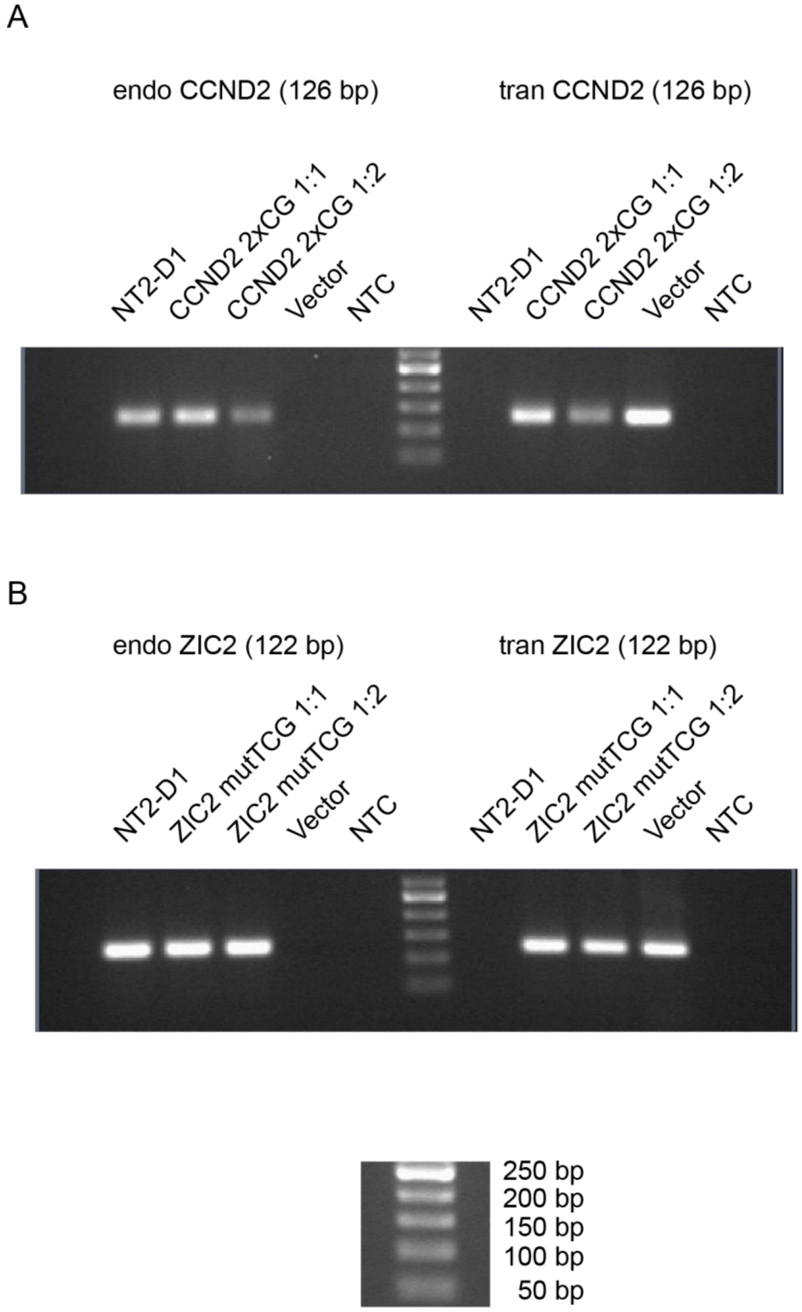

Figure S15. PCR genotyping of NT2-D1 cell lines transduced with mutated *CCND2* and *ZIC2* PTEs. A. PCR genotyping of NT2-D1 cells transduced with the CCND2_PTE_2xCGmut lentiviral construct (two best instances of "AAACGAAA" motif within *CCND2* PTE mutated) using undiluted (1:1) or two-fold diluted (1:2) lentiviral supernatants. **B.** PCR genotyping of NT2-D1 cells transduced with the pLenti-ZIC2_mutTCG lentiviral construct (the best instance of "AAACGAAA" motif within *ZIC2* PTE mutated) using undiluted (1:1) or two-fold diluted (1:2) lentiviral supernatants. For all tested amplicons, NT2-D1 genomic DNA from uninfected cells was used as a positive control for the endogenous amplicon (endo) and as a negative control for the transgenic amplicon (trans).

The correspondent lentiviral vectors were used as positive controls for the transgenic amplicons and as negative controls for the endogenous amplicons. The size of each amplicon is indicated in brackets. In both (**A**) and (**B**) the genotyping confirms the specificity of the endogenous and transgenic amplicons and that cells transduced with the lentiviral constructs contain the expected transgenes. Sequences of corresponding amplicons and primers are indicated in Table S5.

Figure S16

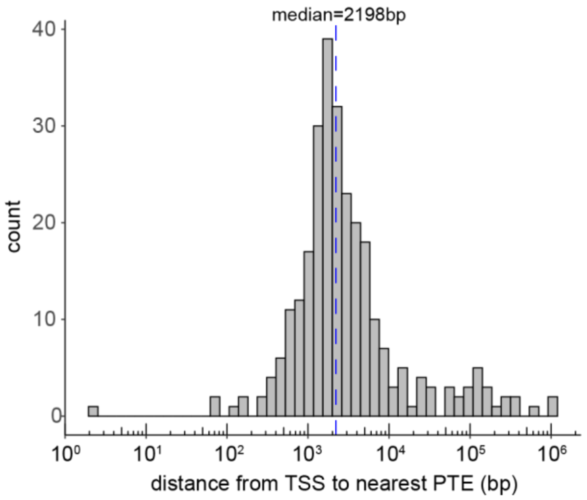

Figure S16. The histogram of distances between putative PTEs and TSSs of their likely target genes.

Figure S17

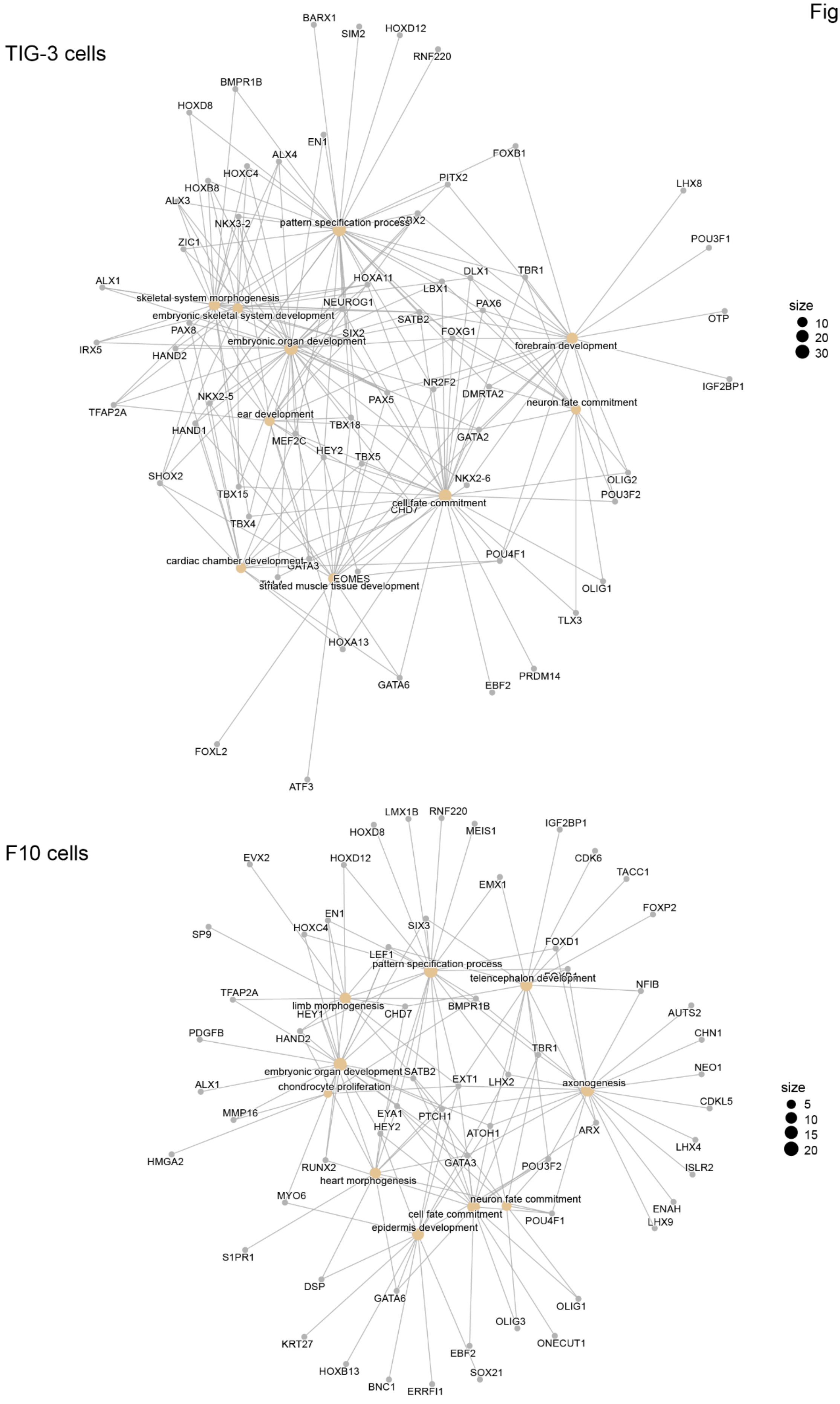

TIG-3 cells
BARX1
SIM2
HOXD12
RNF220
BMPR1B
HOXD8
EN1
FOXB1
ALX4
HOXC4
PITX2
LHX8
HOXB8
ALX3
pattern specification process
NKX3-2
ZIC1
POU3F1
DLX1
TBR1
HOXA11
ALX1
LBX1
skeletal system morphogenesis
NEUROG1
PAX6
embryonic skeletal system development
SATB2
OTP
PAX8
SIX2
FOXG1
IRX5
embryonic organ development
forebrain development
HAND2
NR2F2
IGF2BP1
NKX2-5
DMRTA2
PAX5
neuron fate commitment
TFAP2A
ear development
TBX18
HAND1
GATA2
MEF2C
HEY2
TBX5
SHOX2
NKX2-6
OLIG2
TBX15
POU3F2
cell fate commitment
TBX4
POU4F1
cardiac chamber development
EOMES
striated muscle tissue development
OLIG1
TLX3
HOXA13
PRDM14
GATA6
EBF2
FOXL2
ATF3
size
10
20
30
F10 cells
LMX1B
RNF220
HOXD8
MEIS1
IGF2BP1
EVX2
HOXD12
CDK6
EMX1
TACC1
EN1
FOXP2
SP9
HOXC4
SIX3
FOXD1
LEF1
pattern specification process
telencephalon development
NFIB
TFAP2A
limb morphogenesis
BMPR1B
AUTS2
HEY1
CHD7
PDGFB
CHN1
HAND2
TBR1
NEO1
embryonic organ development
SATB2
ALX1
EXT1
LHX2
axonogenesis
chondrocyte proliferation
CDKL5
MMP16
EYA1
PTCH1
HEY2
ARX
ATOH1
LHX4
HMGA2
ISLR2
RUNX2
GATA3
POU3F2
heart morphogenesis
MYO6
neuron fate commitment
ENAH
cell fate commitment
POU4F1
LHX9
epidermis development
S1PR1
DSP
GATA6
OLIG1
OLIG3
KRT27
ONECUT1
EBF2
HOXB13
SOX21
BNC1
ERRFI1
size
5
10
15
20

Figure S17. PTE-equipped genes of TIG-3 and F10 cells grouped in networks by overrepresented GO terms.

Table S1. Sequence and name of PCR primers for cloning of the corresponding transgenic constructs.

Construct	Primer name	Sequence
ZIC2 1.8kb PTE	ZIC2_1.8_PTE_F	AGCTGGGTCTAGCACACCTTGAAAATGCACTCTTCC
	ZIC2_1.8_PTE_R	GATCACGAGACTAGCCCAAATAATGCCCCCAGAAG
	ZIC2_trans_F	CCGGGCGTCATGCGCTGTTTTAATTCTGCT
	ZIC2_trans_R	CAGCGCATGACGCCCGGCTCTTTAGGGAAAT
ZIC2 mutTCG PTE	ZIC2_1.8_PTE_F	AGCTGGGTCTAGCACACCTTGAAAATGCACTCTTCC
	ZIC2_1.8_PTE_R	GATCACGAGACTAGCCCAAATAATGCCCCCAGAAG
	ZIC2_mutTCG_2.1	AAACAAAAAAAAAGCAAGCGTTCCAGCC
	ZIC2_mutTCG_1.2	TTGCTTTTTTTTTGTTTGTTTCGCGCATTTC
CCND2 PTE 2xCGmut	CCND2_mutTCG_1.1	CATGAAAAAAAAAGAATATTTAATCACATTGGGAAAGTG
	CCND2_mutTCG_1.2	ATTCTTTTTTTTTCATGGTTTTATTCAGCTGTG
	CCND2_mutTCG_2.1	GATCACGAGACTAGCGCTGCTTCAATTGGCTGCAACT
	CCND2_mutTCG_2.2	AGCTGGGTCTAGCACGTGAGAATCTTCTAAACTGCAAGG G
Lenti-Gateway	Gateway_lenti_F	GATCACGAGACTAGCGCTGCCGTTACTAGTGGATCCATC
	Gateway_lenti_R	AGCTGGGTCTAGCACGTGCGGATCCATCAACCACTTTG
ALX4 PTE	ALX4_1.1	ATCCGGTACCGAATTCGACGTCAACAACCTCTCATC
	ALX4_1.2	TGTTATTCGAGTGTACACTGTTAGTTAGGACG
	ALX4_2.1	GTACACTCGAATAACAATTCTTCCAACTTTCCTCC
	ALX4_2.2	GTGCGGCCGCGAATTCCGGAAAGAGTTGGTGAGAG
DMBX1 PTE	DMBX1 1.1	ATCCGGTACCGAATTCATTCCGAGGCAGAGAGAGCGG
	DMBX1 1.2	TCCTAGCATGAGGCCGCGTCTTCGAGGG
	DMBX1 2.1	CGGCCTCATGCTAGGAACCTTCAGGTGGGTG
	DMBX1 2.2	GTGCGGCCGCGAATTCACAGCCAGGTAGGAACTTGC
ALX3 PTE	ALX3_1.1	ATCCGGTACCGAATTCCCAGCCTCGTGAAAGATCG
	ALX3_1.2	CGGCGACCTTGAGAGGGTAAATGGCAGTCG
	ALX3_2.1	CCTCTCAAGGTCGCCGCGCTCTCCGTTGTT
	ALX3_2.2	GTGCGGCCGCGAATTCTGCCTGTGCGGCAGACTGGC
PAX8 PTE	PAX8_1.1	ATCCGGTACCGAATTCGGGCATACCAAGTTTGACAG
	PAX8_1.2	CCCTGACAGTAGATCTAAAGCCCCATTCCTAAC
	PAX8_2.1	AGATCTACTGTCAGGGAAGTTAGAGACATCG
	PAX8_2.2	GTGCGGCCGCGAATTCGGCTGGACCCTTACTCTCTG
NKX2-3 PTE	NKX2-3_1.1	ATCCGGTACCGAATTCTCTCAGACGGTTTGAAGGTG
	NKX2-3_1.2	GTTTGCCATGATGCCAAATTTTCCTAGATTTTATTTGG
	NKX2-3_2.1	TGGCATCATGGCAAACACATGCAAAACAAAC
	NKX2-3_2.2	GTGCGGCCGCGAATTCAAATGGTGGCAAAGTCCTTG
FOXL2 PTE	FOXL2NB_1.1	ATCCGGTACCGAATTCTGAGGTTCCTACCGCCTG
	FOXL2NB_1.2	ACTATACGTTCCAAGAGTTTGTTCCAAACATTAATAG
	FOXL2NB_2.1	TCTTGGAACGTATAGTTGACTCAAAATCGAAAAGTTTG
	FOXL2NB_2.2	GTGCGGCCGCGAATTCTGCGTTTCCTCCGCTATC
ARID3C PTE	ARID3C-c1.1	ATCCGGTACCGAATTCGAGGGACCGACAGAGACAG
	ARID3C-c1.2	TGTGAGCTGAGGAAGCTATGCGCCTGAAC
	ARID3C-c2.1	GCTTCCTCAGCTCACACGTTCCGTGACATG
	ARID3C-c2.2	GTGCGGCCGCGAATTCCGGTCACTGTTGAAACAAACC
Spacer	Spacer Gateway F	ATCCGGTACCGAATTCCCCTGGAGATGTGTCCATG
	Spacer Gateway R	GTGCGGCCGCGAATTCCTGGTGAAACTGTGAGATAATG
	GFI1_1.1	ATCCGGTACCGAATTCGCCAAATACTCCCCATTCCTG

GFI1	GFI1_1.2	GATTCTACTGAGGGCCGGTTATATCCAG
	GFI1_2.1	GGCCCTCAGTAGAATCCGGCTGGTCTGC
	GFI1_2.2	GTGCGGCCGCGAATTCGGAGAAGGGAGCTGATTCG
SYK	SYK_1.1	ATCCGGTACCGAATTCGTCCAGACGTCTTATGAGCTTAG
	SYK_1.2	ACTTTCGTCAGGGAGAAGGAGGACATCAATG
	SYK_2.1	TCTCCCTGACGAAAGTCCGAGCAGTGCATAG
	SYK_2.2	GTGCGGCCGCGAATTCATCTTCCCACAATCCCTCAG
MSX2	MSX2_1.1	ATCCGGTACCGAATTCACCTGGACCTCCTATGCC
	MSX2_1.2	TCTCTGAGTCGGTGGAGACAAATCGGGAAAG
	MSX2_2.1	TCCACCGACTCAGAGAGCTGGGGAGGGAA
	MSX2_2.2	GTGCGGCCGCGAATTCACAGTCTGCACAGGCCAG
NEUROG1	NEUROG1_1.1	ATCCGGTACCGAATTCCAGACACGGCTTCAAACG
	NEUROG1_1.2	CTTTTTGACTCCATACTCCATTCTTCAGCC
	NEUROG1_2.1	GTATGGAGTCAAAAAGCCCACTGGAGAGG
	NEUROG1_2.2	GTGCGGCCGCGAATTCGCAGCGGTAAGAAGACTGG
IHH	IHH_1.1	ATCCGGTACCGAATTCTGAAGTTGCAAAGGGATAGG
	IHH_1.2	TGCTTTGACTCCACTAGCAAGTTGTTCATCTTC
	IHH_2.1	TAGTGGAGTCAAAGCAGGGATTGAAGCC
	IHH_2.2	GTGCGGCCGCGAATTCTCTGAATGCTAATGCAAGAGAG
KDM5B	KDM5B_1.1	ATCCGGTACCGAATTCGAGACTATTGGGAGGTGGAG
	KDM5B_1.2	GAAGACGACTTGAGCTAATACATGCAGAGCG
	KDM5B_2.1	AGCTCAAGTCGTCTTCACAACCACCATATTGAG
	KDM5B_2.2	GTGCGGCCGCGAATTCCTGGGCTTGACTCTCCATTG
MAFB	MAFB_1.1	ATCCGGTACCGAATTCGCAGGGAAAGAAACGCAATG
	MAFB_1.2	TCTGGAAGGTAGCCTTGTCTTATGGTCAAATTG
	MAFB_2.1	AAGGCTACCTTCCAGAACACTCCTCTGGGG
	MAFB_2.2	GTGCGGCCGCGAATTCAGGAAAGGAGAGGGACTCTGG
TGFA	TGFA_1.1	ATCCGGTACCGAATTCGAGATCCCAGGAATTCAATAGG
	TGFA_1.2	AACCGGGCTATCCCAGCTACACCCTTACC
	TGFA_2.1	CTGGGATAGCCCGGTTCCAAGACTGCCTT
	TGFA_2.2	GTGCGGCCGCGAATTCTTGGAAATGAAGGAGGGATGGG

The nucleotides in blue are the 16nt homology sequence required for In-Fusion cloning.
The nucleotides in red indicate introduced mutations.

Table S2. sgRNA and primers used to generate and charachterize the F10 cell lines with deletion of CCND2-PTE, ZIC2-PTE and ALX3-PTE.

sgRNA used to delete PTEs			
**Target**	**sgRNA name**	**sgRNA sequence 5'->3'**	**target DNA sequence 5'->3'(PAM)**
CCND2-PTE	sgRNA-dPTE-CCND2-L	UCCUUGUGGCAAAUUUCAGG	TCCTTGTGGCAAATTTCAGG(AGG)
CCND2-PTE	sgRNA-dPTE-CCND2-R	GCAUGAGCACGGAUGCCACG	GCATGAGCACGGATGCCACG(AGG)
ZIC2-PTE	sgRNA_ZIC2_L	GAUAAUCUGCCAGAUAUGAG	GATAATCTGCCAGATATGAG(AGG)
ZIC2-PTE	sgRNA_ZIC2_R	ACCCAAAUAAUGCCCCCAGA	ACCCAAATAATGCCCCCAGA(AGG)
ALX3-PTE	sgRNA_ALX3_L	CUGCGGCGCGAUCUUUCACG	CTGCGGCGCGATCTTTCACG(AGG)
ALX3-PTE	sgRNA_ALX3_R	UGGCAUUGCACGACCAGUCG	TGGCATTGCACGACCAGTCG(CGG)

Primers flanking the deletion			
**Amplicon name**	**Primer name**	**Primer sequence 5'->3'**	**Fragment size in wild type**
Outside CCND2-PTE	Genomic_PRE_qPCR_1.1_F'	AAACTAACCCAAACCATTTGCTT	2432 bp
Outside CCND2-PTE	Genomic PRE qPCR 1.2 R'	TTGACTTTTACACCCGGAGTTT	
Outside ZIC2-PTE	LINC00554-2.1	CACTTCTTCCTTTGCTGGGT	2571 bp
Outside ZIC2-PTE	LINK00554-exp1.2	GGTGCGACCTAGGTACCCTTTAT	
Outside ALX3-PTE	ALX3_dPTE_1.1	GAGTGGACATAAACGCCGTG	2084 bp
Outside ALX3-PTE	ALX3_dPTE_1.2	AGAGTGGTCAAATCGGGGTC	

Primers located inside the deletion			
**Amplicon name**	**Primer name**	**Primer sequence 5'->3'**	**Fragment size**
Inside CCND2-PTE	Seq. Homolg. BmiI PRE 1 F	AATTATAGCTCATTTTTTCCCC	126 bp, no amplification after PTE deletion
Inside CCND2-PTE	Seq. Homolg. BmiI PRE 1R	TTCCTTCTTTTTAACACATTCC	
Inside ZIC2-PTE	LINC00554_1.1	CTTCCTTTCCTAGTGCCAGACAAC	86 bp, no amplification after PTE deletion
Inside ZIC2-PTE	LINC00554_1.2	GGGAAATGCGCGAAACAAACAA	
Inside ALX3-PTE	ALX3_1F	TGCCATTTACCCTCTCGTTC	120 bp, no amplification after PTE deletion
Inside ALX3-PTE	ALX3_1R	GGCCTTGACAACGAAATACC	

Table S3. Primers for RT-qPCR ananlysis of F10 clones with deleted PTEs.

Amplicon	Primer name	Primer sequence 5'-3'	
CCND2	huCCND2_ex2_F	TCTGGCCATGAATTACCTGG	Fig.5D
	huCCND2_ex2_R	GTAAATGCACAGCTTCTCCG	
ZIC2	ZIC2-3'UTR_1.1	CCACCAACAACGCTTGTGAAT	Fig.5E
	ZIC2-3'UTR_1.2	ACGGAAAAAGCTAACGGCAC	
ALX3	ALX3-ex1.1	CCCGGGCATACACATCAGGA	Fig.5F
	ALX3-ex1.2	AAGAAGCGTCGTAACCGCA	
GAPDH	GAPDH-2.1	AAAACCTGCCAAATATGATG	Fig.5(D-F)
	GAPDH-2.2	TGTTGAAGTCAGAGGAGACC	
CCND2 #1	PRE 1.1 internal F	CATGCATAGGTGCAGACTTTGT	Fig.5(G,J)
	PRE 1.1 internal R	GTGTGCTAGACAATCCAAGCC	
CCND2 #2	Seq. Homolg. BmiI PRE 1 F	AATTATAGCTCATTTTTTCCCC	
	Seq. Homolg. BmiI PRE 1 R	TTCCTTCTTTTTAACACATTCC	
CCND2 #2*	dPTE-spec_F1	ACAGTCAACCTGTTTCACATTTC	
	dPTE-spec_R2	CGATGTGAAAACATGTAGGGC	
CCND2 #3	PRE qPCR 1.2 F'	ATGTAATAGCCATCGTATTAACC	
	Genomic PRE qPCR 1.2 R'	TTGACTTTTACACCCGGAGTTT	
ZIC2 #1	LINC00554-2.1	CACTTCTTCCTTTGCTGGGT	Fig.5(H,K)
	LINC00554-2.2	TGTGTGTCTGAAGTTGAACTACC	
ZIC2 #2	LINC00554-1.1	CTTCCTTTCCTAGTGCCAGACAAC	
	LINC00554-1.2	GGGAAATGCGCGAAACAAACAA	
ZIC2 #2*	LINC00554-dPTE_1.1	AGCTTCAGCAACAACATTTCAT	
	LINC00554-dPTE_1.2	AAACACCTTACCCTCAAAGCA	
ZIC2 #3	LINC00554-exp1.1	TCTGGAAGTGAAGCAGGACGA	
	LINC00554-exp1.2	GGTGCGACCTAGGTACCCTTTAT	
ALX3 #1	ALX3_dPTE_1.1	GAGTGGACATAAACGCCGTG	Fig.5(I,L)
	ALX3_dPTE_1.4	AAGTCGAGGAGAGTCCCAAGT	
ALX3 #2	ALX3_1F	TGCCATTTACCCTCTCGTTC	
	ALX3_1R	GGCCTTGACAACGAAATACC	
ALX3 #2*	ALX3-dPTE_1.3	GGGAGAAGAGCGAGGTGC	
	ALX3_dPTE_1.2	AGAGTGGTCAAATCGGGGTC	
ALX3 #3	ALX3-dPTE_2.3	TTCCGTGGAAGCCGTGG	
	ALX3_dPTE_2.2	CTGCTTATCTCTCCCGCTCG	
KLF4	KLF14_1F	GTAAGGAAATGCAGCACATG	Fig.5(G-L)
	KLF14_1R	TGAGAGCAATGAAGACACGT	
Spacer	Spacer qPCR F	AGCTAACAGTGACAATCACCTT	Fig.5(G-L)
	Spacer qPCR R	AGGAGGAGATATAGTTGCAAAG	

Table S4. List of antibodies used in ChIP

name	company, cat#	host	concentration	µl per ChIP
RING1B	MBL, #D139-3, clone 3-3	mouse monoclonal	1 mg/ml	2
BMI1	SHDS, 8/6/15, clone 1H1	mouse monoclonal	64 µg/ml	5
SUZ12	Cell signaling, #3737P	rabbit monoclonal	n/a	5
H3K27me3	Abcam, #ab6002	mouse monoclonal	1 mg/ml	1
H3K27me3	Cell signaling, #9733S	rabbit monoclonal	n/a	2
MEL18	Abnova, #H00007703-M03, clone 4D6	mouse moloclonal	0.42 mg/ml	3
CBX2	Abcam, #ab80044	rabbit polyclonal	1 mg/ml	2

Table S5. PCR primers used for ChIP-qPCR and genotyping and their corresponding amplicons.

Amplicon	Primer name	Sequence
endogenous CCND2 PTE	Seq. Homolg. BmiI PRE 1 F	AATTATAGCTCATTTTTTCCCC
	Seq. Homolg. BmiI PRE 1 R	TTCCTTCTTTTTAACACATTCC
transgenic CCND2 PTE	Mut. PRE qPCR 1 F'	AATTATAGCTCATTTCCGCGG
	Seq. Homolg. BmiI PRE 1 R	TTCCTTCTTTTTAACACATTCC
ZIC2 (1)	ZIC2-exp1.1	ATGTGCTCAACGGGCAGATG
	ZIC2-exp1.2	GCCGTACTGGTTGTGGAGTTG
ZIC2 (2)	LINC00554-4.1	CGCTGCTCCGATATTGATCC
	LINC00554-4.2	CCCCTACCCTGGCTCATTAG
ZIC2 (3)	LINC00554-2.1	CACTTCTTCCTTTGCTGGGT
	LINC00554-2.2	TGTGTGTCTGAAGTTGAACTACC
ZIC2 (4)	LINC00554-5.1	ATCTCACATAGGGTGCCATTG
	LINC00554-5.2	ACAATGTTGGAGGCACAAGAG
ZIC2 (5)	LINC00554-1.1	CTTCCTTTCCTAGTGCCAGACAAC
	LINC00554-1.2	GGGAAATGCGCGAAACAAACAA
ZIC2 (6)	LINC00554-6.1	AGAGCGCGTTGAGCTATTTCT
	LINC00554-6.2	CTGGCGTTTGTTGGCTGTTG
ZIC2 (7)	LINC00554-exp1.1	TCTGGAAGTGAAGCAGGACGA
	LINC00554-exp1.2	GGTGCGACCTAGGTACCCTTTAT
endogenous ZIC2 PTE	LINC00554-1.1	CTTCCTTTCCTAGTGCCAGACAAC
	ZIC2_qPCR_endo_R	GCAGAATTAAAACAGCGCAAAGA
transgenic ZIC2 PTE	LINC00554-1.1	CTTCCTTTCCTAGTGCCAGACAAC
	ZIC2_qPCR_trans_R	GCAGAATTAAAACAGCGCATGAC
desert	Spacer qPCR F	AGCTAACAGTGACAATCACCTT
	Spacer qPCR R	AGGAGGAGATATAGTTGCAAAG
transgenic desert	Spacer qPCR 1.2 F'	CCCGTGCTTTCAGAAGTGTGT
	plenti - CMV CpG qPCR R2	AAGAAAGCTGGGTCTAGCAC
*ALX4	ALX4 H3K27me3 2.1 F	CAACCCCGAGACAGGAACAA
	ALX4 H3K27me3 2.2 R	AGGCGAGGTGATGCTCAAAA
endogenous ALX4 PTE	ALX4-PRE1.1	CTAACTAACAGTGTACACATAC
	ALX4-PRE1.2	CAATCGACTTGCATGATCTTG
transgenic ALX4 PTE	ALX4_F_trans	CTAACTAACAGTGTACACTCGA
	ALX4-PRE1.2	CAATCGACTTGCATGATCTTG
endogenous DMBX1 PTE	DMBX1_1F	CGATTCTGCACGAATTTTCC
	DMBX1_1R	CCTGAAGGTTCCTAGACCA
transgenic DMBX1 PTE	DMBX1_1F	CGATTCTGCACGAATTTTCC
	DMBX1_R_trans	CCTGAAGGTTCCTAGCATG
endogenous ALX3 PTE	ALX3_1F	TGCCATTTACCCTCTCGTTC
	ALX3_1R	GGCCTTGACAACGAAATACC
transgenic ALX3 PTE	ALX3_F_trans	TGCCATTTACCCTCTCAAGG
	ALX3_1R	GGCCTTGACAACGAAATACC
endogenous PAX8 PTE	PAX8_1F	ATGGGGCTTTAGATCTCACA
	PAX8_1R	CACCAGTCCCGAAAATAGTTC
transgenic PAX8 PTE	PAX8_F_trans	ATGGGGCTTTAGATCTACTG
	PAX8_1R	CACCAGTCCCGAAAATAGTTC
endogenous NKX2-3 PTE	NKX2-3_1F	TCCCAGGAAAGAGAAATAGTGG
	NKX2-3_1R	GTTTTGCATGTGTTTGCAGGA

transgenic NKX2-3 PTE	NKX2-3_1F	TCCCAGGAAAGAGAAATAGTGG
	NKX2-3_R_trans	GTTTTGCATGTGTTTGCCATG
endogenous FOXL2 PTE	FOXL2NB_1F	TGGAACAAACTCTTGGCTGT
	FOXL2NB_1R	GTTGTACTAGGTGTATGGGCT
transgenic FOXL2 PTE	FOXL2NB_F_trans	TGGAACAAACTCTTGGAACG
	FOXL2NB_1R	GTTGTACTAGGTGTATGGGCT
endogenous ARID3C POC	ARID3C-1.1	GCACTGGGCTTCCACATACA
	ARID3C-1.2	TCACGGAACGTGTGAGACTG
transgenicARID3C POC	ARID3C-1.1	GCACTGGGCTTCCACATACA
	ARID3C_R_trans	TCACGGAACGTGTGAGCTGA
empty	ccdB_F	AGAGAGCCGTTATCGTCTG
	ccdB_R	GACTTTATCTGACAGCAGACG
endogenous KCNA2	KCNA2_1F	GAACCTCCAGGAGAGCTCA
	KCNA2_1R	GCCTACTTCATTACGTTGGC
transgenic KCNA2	KCNA2_F_trans	GAACCTCCAGGAGAGAGTG
	KCNA2_1R	GCCTACTTCATTACGTTGGC
endogenous GFI1	GFI1_F	GGATATAACCGGCCCTTGAC
	GFI1_R	CGAGCAATCCAGCTTCAGG
transgenic GFI1	GFI1_trans_F	GGATATAACCGGCCCTCAGT
	GFI1_R	CGAGCAATCCAGCTTCAGG
endogenous SYK	SYK_F	TCATTGCCTTCACACAGGC
	SYK_R	CACTGCTCGGACTTTCACTG
transgenic SYK	SYK_F	TCATTGCCTTCACACAGGC
	SYK_trans_R	CACTGCTCGGACTTTCGTCA
endogenous MSX2	MSX2_F	CCGATTTGTCTCCACCTCAG
	MSX2_R	CCAAGCTCTGACCATGAATAGG
transgenic MSX2	MSX2_trans_F	CCGATTTGTCTCCACCGACT
	MSX2_R	CCAAGCTCTGACCATGAATAGG
endogenous NEUROG1	NEUROG1_F	GGGCATTGGCACTAAAATAGG
	NEUROG1_R	CTCCAGTGGGCTTTTTTCAG
transgenic NEUROG1	NEUROG1_F	GGGCATTGGCACTAAAATAGG
	NEUROG1_trans_R	CTCCAGTGGGCTTTTTGACT
endogenous IHH	IHH_F	GGCCAAAGGAGCTATTACTG
	IHH_R	GCTTCAATCCCTGCTTTTCAG
transgenic IHH	IHH_F	GGCCAAAGGAGCTATTACTG
	IHH_trans_R	GCTTCAATCCCTGCTTTGACT
endogenous KDM5B	KDM5B_F	TGGGAGTTATGTGCTTATGGG
	KDM5B_R	TGGTGGTTGTGAAGACTCAG
transgenic KDM5B	KDM5B_F	TGGGAGTTATGTGCTTATGGG
	KDM5B_trans_R	TGGTGGTTGTGAAGACGACT
endogenous MAFB	MAFB_F	GACCATAAGACAAGGCTGTAG
	MAFB_R	TGAGAGAGAGAACCGAGAGG
transgenic MAFB	MAFB_trans_F	GACCATAAGACAAGGCTACCT
	MAFB_R	TGAGAGAGAGAACCGAGAGG
endogenous TGFA	TGFA_F	GCAACAGTCAATACATGCACAC
	TGFA_R	CAGTCTTGGAACCGGATGC
transgenic TGFA	TGFA_F	GCAACAGTCAATACATGCACAC
	TGFA_trans_R	CAGTCTTGGAACCGGGCTA
endogenous OVOL1	OVOL1_1F	TTAGCGACTCCTTCCCTGTT
	OVOL1_1R	CCGCTTCCGTAACTGAAG

transgenic OVOL1	OVOL1_1F	TTAGCGACTCCTTCCCTGTT
	OVOL1_R_trans	CCGCTTCCGTAACTTCCA

Table S6. High-confidence PTEs in NT2-D1 cells

chr	Position Accuracy	Center coordinate	NT2 MEL18 score	MEL18 Score Accuracy	NT2 MEL18 Score Quartile	Target Gene	Distance to target gene (bp)	Target Gene transcription in NT2-D1 (RPKM)	Target Gene transcription in TIG-3 (RPKM)
chr1	21	3650557	131.0631348	3.62671455	Q3	TP73	1959	0.347265224	0.053459277
chr1	103.5	18633384.5	254.3365209	6.32235627	Q4	PAX7	-2538.5	0	0
chr1	8	24928224	119.8672942	45.83623623	Q3	RUNX3	2051	0.572157632	0.253036946
chr1	22.5	46490794.5	415.8132194	78.4101093	Q4	DMBX1	-259.5	0.117192013	0
chr1	23.5	47231648.5	589.0146055	151.4177156	Q4	TAL1	65.5	0.104939009	0
chr1	67.5	47443596.5	277.1903841	15.95008164	Q4	FOXD2	-5552.5	0.186410985	0.111504046
chr1	36	50417801	164.1098129	34.14591123	Q3	DMRTA2	4863	0	0
chr1	10.5	50424735.5	344.1207346	31.33010776	Q4	DMRTA2	-1293.5	0	0
chr1	73	50979419	381.0932095	29.73662202	Q4	CDKN2C	-9174	0.01192147	5.890185457
chr1	241	61084247	119.0058007	0.955366109	Q3	NFIA	-1686	0.449713922	0.215234737
chr1	129.5	63325480.5	296.3204197	50.97160739	Q4	FOXD3	-2913.5	5.04975125	0
chr1	205.5	65003692.5	138.9127153	25.34688336	Q3	JAK1	-36757.5	5.321104366	26.0264311
chr1	39.5	65150396.5	134.3951793	63.07733921	Q3	AK4	-1402.5	9.922084486	0.089315809
chr1	107.5	75133087.5	423.8952816	50.83333731	Q4	LHX8	1300.5	0.045483298	0
chr1	72.5	90711416.5	152.4842643	6.360332744	Q3	BARHL2	5884.5	0	0
chr1	178	90716143	181.016926	2.316464555	Q3	BARHL2	1158	0	0
chr1	89.5	90720497.5	195.1796991	12.04013132	Q4	BARHL2	-3196.5	0	0
chr1	25	92482342	169.407726	3.516235819	Q3	GFI1	1471	0.019467151	0.024536663
chr1	23.5	110068767.5	540.2232399	112.1225482	Q4	ALX3	1903.5	3.038087344	0.470258379
chr1	50.5	156436030.5	161.6176769	4.447179835	Q3	MEF2D	54655.5	3.082561549	5.916149187
chr1	68	165353756	254.3382163	14.26636306	Q4	LMX1A	2465	0.022453561	0
chr1	29	180228217	235.9638481	18.90959322	Q4	LHX4	155	0.793908498	1.009061969
chr1	203.5	190476960.5	174.2659819	1.703003036	Q3	BRINP3	455.5	0.010908937	0.192496829
chr1	165	197912985	118.3334472	12.71340328	Q3	LHX9	-521	0	0
chr1	60	197919431	257.2042287	28.71589151	Q4	LHX9	-2059	0	0
chr1	12	200043514	338.6825042	10.77567806	Q4	NR5A2	-923	1.452330089	0
chr1	107.5	213986746.5	232.1504787	18.39082402	Q4	PROX1	-3507.5	0.411385571	0.035454085
chr1	51	214186885	166.7533786	0.688521814	Q3	PROX1	-197547	0.411385571	0.035454085
chr1	47	218166980	118.4469432	22.05703016	Q3	RRP15	118313	10.60802616	4.247131584
chr1	158.5	220878356.5	190.8636859	45.37665941	Q4	HLX	1086.5	0.129624405	1.165446649
chr1	15	220880785	188.8008417	16.23786281	Q4	HLX	-1342	0.129624405	1.165446649
chr10	17	8039493	162.0812025	0.709467287	Q3	GATA3	5927	0.985305173	0.074588114
chr10	203.5	21498808.5	243.0486105	0.994398444	Q4	MLLT10	25807.5	1.901200845	6.222162875
chr10	28.5	22319517.5	335.4302111	41.82563179	Q4	BMI1	1581.5	1.856026856	4.139566406
chr10	38	22322712	171.8893464	12.5870996	Q3	SPAG6	22749	0.0745995	0
chr10	145.5	22338696.5	202.0333661	24.32019114	Q4	SPAG6	6764.5	0.0745995	0
chr10	32.5	22475429.5	159.1762521	12.66476088	Q3	SPAG6	-129933.5	0.0745995	0
chr10	104	26219149	229.5202228	15.44102978	Q4	GAD2	-2375	0.18037491	0
chr10	44	75397846	298.8219296	9.480935482	Q4	ZNF503	3917	0.055964767	2.492369408
chr10	112.5	92693270.5	220.643993	18.70606603	Q4	HHEX	-3315.5	0	2.765942688
chr10	168	99528701	392.199049	48.59451256	Q4	NKX2-3	4241	0	0
chr10	48	100747387	139.2822095	32.42310445	Q3	PAX2	-1805	0.006977185	0
chr10	7.5	101140395.5	128.6085718	4.965159286	Q3	TLX1	-9095.5	0.010981934	0
chr10	84.5	101226475.5	132.5758775	6.413112738	Q3	LBX1	2986.5	0	0
chr10	96.5	101237807.5	232.7741254	45.56483802	Q4	LBX1	-8345.5	0	0

chr10	220	117132203	171.6878451	7.734410555	Q3	SHTN1	-5618	0.836273597	3.724437173
chr10	30.5	117138623.5	112.456242	13.90034546	Q3	VAX1	-354.5	0.01020781	0
chr10	74.5	117161256.5	212.4959139	15.02339542	Q4	VAX1	-22987.5	0.01020781	0
chr10	58.5	117533895.5	141.1194823	36.78751537	Q3	EMX2	8850.5	0.051003802	0
chr10	67	117544601	169.6638314	19.4834258	Q3	EMX2	-1855	0.051003802	0
chr10	136	123144360	108.9068798	13.96124183	Q3	HMX2	-1939	0	0
chr10	58.5	123152044.5	134.836725	1.984959798	Q3	HMX2	-3908.5	0	0
chr10	134	129957905	146.4442597	18.53730488	Q3	EBF3	6368	0.0794749	1.270464636
chr10	19	129962280	249.64197	15.33959454	Q4	EBF3	1993	0.0794749	1.270464636
chr10	148.5	129966173.5	155.6834254	23.19255725	Q3	EBF3	-1900.5	0.0794749	1.270464636
chr11	142.5	2141860.5	132.8263517	11.16591601	Q3	IGF2	-918.5	0.126172156	1.60328253
chr11	210.5	13279673.5	148.4702477	37.81137052	Q3	ARNTL	-1875.5	0.458269378	3.380082212
chr11	231	14975619	113.7695522	1.754001798	Q3	CALCA	-3259	0	0
chr11	20.5	16611911.5	189.1813193	15.15039007	Q4	SOX6	126564.5	0.015775607	1.953913842
chr11	120.5	20157732.5	110.2293407	19.61566973	Q3	DBX1	2741.5	0	0.096212885
chr11	81	31802004	182.535991	42.4639661	Q3	PAX6	-1344	0.1673198	0.073353796
chr11	237	31809336	185.6734874	12.3885427	Q3	PAX6	622	0.1673198	0.073353796
chr11	44	31821413	153.5918498	3.772427108	Q3	PAX6	-3453	0.1673198	0.073353796
chr11	23.5	31827859.5	127.4248187	1.455876661	Q3	PAX6	-9899.5	0.1673198	0.073353796
chr11	178.5	32440304.5	156.1608359	8.09884864	Q3	WT1	-4766.5	0.062380034	0.014976123
chr11	97	44305458	386.6584261	33.55773441	Q4	ALX4	4680	0.106276779	0.029767268
chr11	14.5	122976084.5	182.5809851	32.58107091	Q3	BSX	5748.5	0.042081769	0
chr11	51.5	122978191.5	138.5662268	20.1547234	Q3	BSX	3641.5	0.042081769	0
chr12	7	4268228	533.5008469	104.0435248	Q4	CCND2	5534	94.30760963	0.346915685
chr12	31	15788129	115.1071128	6.192425732	Q3	EPS8	1027	0.902561435	8.787371218
chr12	52.5	27968848.5	175.9324892	9.265836817	Q3	PTHLH	1126.5	0	0.212161747
chr12	234.5	27971663.5	143.9414707	13.61658604	Q3	PTHLH	357.5	0	0.212161747
chr12	27	53966425	126.2364473	41.39919004	Q3	HOXC11	6701	0	0
chr12	112	54030001	186.1932696	53.34150438	Q3	HOXC6	-1561	0.026329235	0
chr12	184.5	54125446.5	110.111441	24.19089157	Q3	HOXC4	-71611.5	0	0
chr12	9.5	89706731.5	119.5074316	0.709158203	Q3	ATP2B1	2103.5	8.753290628	17.57034692
chr12	254	98893348	125.747315	19.73962562	Q3	ANKS1B	1425	0.370766367	0.190904781
chr12	57	102961438	172.8048259	5.295951589	Q3	PAH	-2998	0.230964051	0
chr12	157.5	113473396.5	143.8420742	42.39814244	Q3	LHX5	-1526.5	0.085052021	0
chr12	31	114440458	188.3691292	35.66175717	Q4	TBX5	-31751	0.406346657	20.95892496
chr12	17.5	114480704.5	124.6854404	23.32889586	Q3	TBX5	-71997.5	0.406346657	20.95892496
chr12	131	114670705	283.8760153	9.147138781	Q4	TBX5	-261998	0.406346657	20.95892496
chr12	91.5	114681503.5	169.8880379	17.90263179	Q3	TBX5	-272796.5	0.406346657	20.95892496
chr12	182	114697228	203.3157689	19.78868435	Q4	TBX3	-13054	0.157496498	29.92452065
chr12	144.5	123971210.5	172.3112256	16.49727314	Q3	CCDC92	847.5	0.46175619	12.87798908
chr12	67.5	130159567.5	271.0379092	0.279930888	Q4	FZD10	2896.5	0.693079814	0
chr13	10.5	20159748.5	184.4200392	20.27273904	Q3	GJA3	1302.5	0.302291852	0.410142199
chr13	67.5	27795349.5	146.0503435	12.47661239	Q3	GSX1	-2866.5	0	0
chr13	3.5	27822486.5	132.9666047	28.27365482	Q3	GSX1	-30003.5	0	0
chr13	25.5	27979945.5	144.1548525	17.21688265	Q3	URAD	8746.5	0	0
chr13	19.5	35474693.5	294.3990345	18.23614099	Q4	MAB21L1	1994.5	0.020324322	0.239092495
chr13	78	50129187	195.6757202	19.12535288	Q4	KCNRG	-113743	1.096758633	2.29134687
chr13	63	78597868	353.8106268	21.64012972	Q4	POU4F1	5683	0.116091441	0
chr13	21	94709890	117.6580979	39.31861938	Q3	SOX21	2654	3.895683918	0
chr13	31.5	99995856.5	384.3863591	52.79728726	Q4	ZIC2	-14072.5	26.25085069	0.016482686

chr13	42.5	111534322.5	117.8963396	1.289665037	Q3	LOC1079	88926.5	NA	NA	
chr13	48	112054674	173.0437286	30.44465146	Q3	SOX1	12475	0.050338319	0	
chr13	77.5	112060684.5	129.4539099	13.56937289	Q3	SOX1	6464.5	0.050338319	0	
chr13	52.5	112069808.5	307.169909	25.58192751	Q4	SOX1	-2659.5	0.050338319	0	
chr14	224	28759426	139.1425316	21.50698013	Q3	FOXG1	7361	0.055286467	3.406764425	
chr14	160.5	28764218.5	177.0328639	30.90884656	Q3	FOXG1	2568.5	0.055286467	3.406764425	
chr14	92.5	28778408.5	281.9377149	74.37957237	Q4	FOXG1	-11621.5	0.055286467	3.406764425	
chr14	249	35532700	160.2348432	24.96356862	Q3	INSM2	1464	0	0	
chr14	82.5	36516156.5	152.0602519	8.017157109	Q3	NKX2-1	3398.5	0	0	
chr14	13	36667617	160.268416	7.561043995	Q3	PAX9	-5757	0.01901603	0.023968063	
chr14	20	37586998	276.3819366	32.33892774	Q4	TTC6	6161	0.004192649	0.049321735	
chr14	64.5	37596792.5	112.8022627	5.220934241	Q3	TTC6	-1163.5	0.004192649	0.049321735	
chr14	1.5	41609313.5	154.5458081	3.576823126	Q3	LRFN5	-2437.5	0.941968604	1.9288982	
chr14	8	56815754	252.4076589	25.69311867	Q4	OTX2	-5276	31.49919889	0	
chr14	49.5	60513044.5	162.5450585	12.54505848	Q3	SIX6	-3898.5	0.180252622	0.033658203	
chr14	111	60643895	253.1779366	15.77486945	Q4	SIX1	5581	0.032982585	35.30823117	
chr14	109.5	60652211.5	212.7731579	33.35090233	Q4	SIX1	-2723.5	0.032982585	35.30823117	
chr14	163	74240823	208.0448841	20.83572542	Q4	VSX2	-1374	0.038717434	0	
chr14	37.5	94774601.5	281.2810491	16.55245237	Q4	GSC	-4489.5	0.198764689	0	
chr14	58.5	103846258.5	165.6270023	15.23944262	Q3	PPP1R13	856.5	0.633450939	0.486885818	
chr15	65.5	36886749.5	231.9279676	8.866109041	Q4	MEIS2	209514.5	0.225740902	8.645871414	
chr15	36.5	37105104.5	171.7423848	20.11804741	Q3	MEIS2	-3794.5	0.225740902	8.645871414	
chr15	43.5	37108794.5	277.6255088	19.48594096	Q4	MEIS2	-7484.5	0.225740902	8.645871414	
chr15	33.5	55740807.5	183.4310788	28.19674037	Q3	PRTG	1725.5	2.875604639	0.331114741	
chr15	119.5	58064624.5	400.0606174	103.5490403	Q4	ALDH1A2	1085.5	0.062208796	0	
chr15	84.5	59996164.5	234.5733144	34.37957751	Q4	FOXB1	8146.5	0.749165094	0	
chr15	81.5	60001753.5	312.8377326	27.27850953	Q4	FOXB1	2557.5	0.749165094	0	
chr15	114	68823258	141.984431	5.883733505	Q3	ANP32A	-2364	42.48597246	7.707482818	
chr15	44	68843620	260.5103361	32.99102673	Q4	ANP32A	-22726	42.48597246	7.707482818	
chr15	31.5	70762314.5	109.3539065	15.55554732	Q3	UACA	1174.5	4.038231956	48.51256356	
chr15	34.5	76337341.5	228.4979558	12.97483097	Q4	ISL2	-568.5	0.028269284	0.35630992	
chr15	90.5	76344528.5	272.9169228	4.699903081	Q4	ISL2	-7755.5	0.028269284	0.35630992	
chr15	7.5	76348063.5	241.0385891	10.22459368	Q4	ISL2	-11290.5	0.028269284	0.35630992	
chr15	133	83282586	153.5245754	61.27649772	Q3	BNC1	602	0.061978909	1.439284575	
chr15	178.5	96328795.5	214.0349243	24.14330506	Q4	NR2F2	-2749.5	0.033844487	51.01496924	
chr15	204	96338096	204.30715	2.863389958	Q4	NR2F2	-4331	0.033844487	51.01496924	
chr15	64.5	96350354.5	284.3504513	56.44353944	Q4	NR2F2	-16589.5	0.033844487	51.01496924	
chr15	209	96362738	200.29085	18.12031001	Q4	NR2F2	-28973	0.033844487	51.01496924	
chr15	61.5	96410921.5	297.8088562	9.361683766	Q4	NR2F2	-77156.5	0.033844487	51.01496924	
chr15	236.5	98652669.5	125.6483778	9.369370886	Q3	IGF1R	-1665.5	7.023642028	10.85509799	
chr16	140	54926850	133.8583661	1.881605976	Q3	IRX5	4015	0.101829941	0.018013733	
chr16	55	54938510	148.9756427	41.99901124	Q3	IRX5	-6992	0.101829941	0.018013733	
chr16	16.5	73061877.5	247.7739829	0.481784164	Q4	ZFHX3	-2857.5	0.47591436	6.453653327	
chr16	291.5	85898333.5	108.762927	0.623402049	Q3	IRF8	828.5	0.044389785	0	
chr16	75	86499888	176.2647837	0.091803776	Q3	FOXF1	10639	0.023061204	26.02434622	
chr16	293	86503059	142.7143115	0.115234335	Q3	FOXF1	7468	0.023061204	26.02434622	
chr16	204	86565323	212.190239	6.414426137	Q4	FOXC2	1506	0.20185747	1.327429114	
chr17	60.5	20010570.5	112.4983561	13.8549436	Q3	SPECC1	-1211.5	4.041243192	14.00840609	
chr17	112	35374785	138.967607	15.90566266	Q3	SLFN11	-1165	0.108748028	7.86310564	
chr17	60	48554131	145.7839155	41.8129315	Q3	HOXB3	1674	0.006641144	4.817854078	

chr17	46.5	48580193.5	267.5665817	5.357380119	Q4	HOXB4	-1844.5	0	9.010977977
chr17	252	48718140	238.3733097	35.27261798	Q4	PRAC2	2662	0.223122371	0
chr17	84.5	49964938.5	160.6961926	9.339562262	Q3	DLX4	4031.5	0.127498498	0.02142678
chr17	38.5	61403468.5	284.5914228	41.76196277	Q4	TBX2	-3625.5	0.00479724	15.75316477
chr17	79.5	61453902.5	211.728911	9.209558775	Q4	TBX4	1212.5	0.007000332	0.023528838
chr17	16.5	79792699.5	119.5834403	4.059972166	Q3	CBX8	4376.5	0.945859894	1.252385579
chr18	24.5	22167594.5	336.6528378	88.9784102	Q4	GATA6	1994.5	0.140822361	0.407579797
chr18	211	22176283	196.7430887	8.295658702	Q4	GATA6	-6694	0.140822361	0.407579797
chr18	30.5	22347829.5	134.588032	7.650062176	Q3	GATA6	-178240.5	0.140822361	0.407579797
chr18	196	31040804	160.3109036	38.60554671	Q3	DSC3	1937	0.316556132	0.599696577
chr18	159.5	57801218.5	118.2840151	16.73364776	Q3	ATP8B1	2095.5	0.241429511	10.04102097
chr18	3	75206005	331.745918	35.04047537	Q4	ZADH2	-493	0.791588307	3.748306067
chr19	6	6108503	112.7699045	0.948219727	Q3	RFX2	1996	0.152597052	0.792655018
chr19	22	6588888	233.1066345	63.79258795	Q4	CD70	2261	0.226494867	0
chr19	97	13013851	183.3570014	31.37159527	Q3	NFIX	10121	0.087207091	11.73547261
chr19	66.5	13097256.5	305.737068	62.13236001	Q4	NFIX	-72477.5	0.087207091	11.73547261
chr19	75.5	31355639.5	151.5320658	8.932988634	Q3	TSHZ3	-6204.5	0.348748486	9.417856937
chr2	29	19362178	188.3261609	0.239713901	Q4	OSR1	-3556	0.039127685	6.098877459
chr2	4	24493164	108.2996246	24.19115767	Q3	NCOA1	-1114	4.064599367	6.635015218
chr2	18	35178474	133.332515	29.84413489	Q3	CRIM1	1177304	3.113558109	260.5051802
chr2	49.5	44807486.5	296.5688036	22.92539107	Q4	SIX3	134215.5	0.126912831	0
chr2	119.5	44927358.5	275.386727	12.21007713	Q4	SIX3	14343.5	0.126912831	0
chr2	95	44930865	375.7407138	19.92680419	Q4	SIX3	10837	0.126912831	0
chr2	261	45168364	109.6425932	4.797668586	Q3	SIX2	-158720	0.099717812	0
chr2	20.5	63046743.5	312.1621902	20.68948921	Q4	OTX1	2991.5	0	0
chr2	81	63049111	270.3496535	15.11540091	Q4	OTX1	624	0	0
chr2	91	63060547	336.1728349	28.80855798	Q4	OTX1	-9717	0	0
chr2	148	66427583	170.1508305	8.910613866	Q3	MEIS1	7542	0.041212893	25.39085964
chr2	56.5	66431698.5	144.8572378	13.08842144	Q3	MEIS1	3426.5	0.041212893	25.39085964
chr2	148.5	66437376.5	285.6699035	3.499406468	Q4	MEIS1	-2251.5	0.041212893	25.39085964
chr2	26	66578965	339.0056264	22.72666243	Q4	MEIS1	-143840	0.041212893	25.39085964
chr2	5.5	70791176.5	130.9265086	2.406796159	Q3	FIGLA	-534.5	0	0
chr2	126.5	72915490.5	109.2623817	8.179432879	Q3	EMX1	1975.5	0.083486021	0
chr2	184	72919568	260.0286807	19.23477578	Q4	EMX1	-2049	0.083486021	0
chr2	31	72925946	182.1372333	31.23477594	Q3	EMX1	-8427	0.083486021	0
chr2	5	74514071	214.8110994	30.31494414	Q4	TLX2	379	0.109819338	0
chr2	5.5	91442637.5	140.6207315	8.837708262	Q3	LOC1005(	3144589.5	0	0
chr2	71	94273436	147.4136609	35.97957868	Q3	LOC1005(	313791	0	0
chr2	15.5	98729726.5	126.7241717	23.81721688	Q3	MGAT4A	1404.5	0.364280353	0.034982395
chr2	263.5	104846367.5	231.28747	2.045877594	Q4	POU3F3	7747.5	0	0
chr2	89	104858468	513.2366277	79.90340878	Q4	POU3F3	-4353	0	0
chr2	85.5	113275714.5	279.5256395	29.1380369	Q4	PAX8	3205.5	0.385043617	1.49189191
chr2	32	113500860	136.0415309	6.196477505	Q3	FOXD4L1	-1776	0	0
chr2	0	113504419	151.6272388	1.085936127	Q3	FOXD4L1	-5335	0	0
chr2	6.5	118842393.5	273.3930328	62.34659596	Q4	EN1	5253.5	0.009973465	0
chr2	96.5	118852193.5	160.1316181	6.341297295	Q3	EN1	-4546.5	0.009973465	0
chr2	10	172083704	222.1502718	2.655819639	Q4	DLX1	1803	0.049586911	0.208333398
chr2	98	172089156	278.2300556	34.0439652	Q4	DLX1	-3649	0.049586911	0.208333398
chr2	11	172093568	300.6077015	1.330268331	Q4	DLX1	-8061	0.049586911	0.208333398
chr2	64	174331921	283.8609672	0.828882165	Q4	SP9	3033	0	0

chr2	55	176128173	196.76991	9.366885753	Q4	HOXD8	1532	0.022473489	0
chr2	163.5	176132125.5	329.813206	13.14668237	Q4	HOXD8	-2420.5	0.022473489	0
chr2	179	176637075	150.3185684	22.60539341	Q3	MTX2	-367633	11.45869172	1.908343637
chr2	16.5	199461414.5	305.9053914	17.92094187	Q4	SATB2	2.5	2.281157995	1.698518324
chr2	111.5	222289189.5	369.3774152	20.15270608	Q4	PAX3	9807.5	0.076992427	0
chr2	63.5	222313971.5	210.1609676	9.800242157	Q4	PAX3	-14974.5	0.076992427	0
chr20	65.5	21107042.5	151.2684749	9.020311452	Q3	KIZ	18932.5	2.755156657	6.152141028
chr20	73	21392260	194.423787	25.43156225	Q4	NKX2-4	5265	0.474414114	0
chr20	36	21398462	167.9700221	33.47390968	Q3	NKX2-4	-937	0.474414114	0
chr20	84.5	21511462.5	166.0970218	40.10453329	Q3	NKX2-2	1536.5	0.141308859	0
chr20	133	38726367	114.5425813	9.488390902	Q3	SLC32A1	-1881	0.057384231	0
chr20	63.5	46896082.5	130.7382956	7.633842326	Q3	EYA2	-1151.5	0.243609676	0.21723192
chr21	48.5	33024267.5	116.8323619	10.24333296	Q3	OLIG2	1667.5	1.337906344	0
chr21	26	33072339	236.5053869	19.4510891	Q4	OLIG1	-2198	0.129624405	0
chr21	164	34885677	150.1538075	50.92896966	Q3	RUNX1	1704	0.448003905	8.505441709
chr21	58	34891811	214.4307591	17.33779973	Q4	RUNX1	-1586	0.448003905	8.505441709
chr21	157.5	36694145.5	290.6771898	41.45243782	Q4	SIM2	4969.5	0.167969681	0.757515471
chr21	73	36711408	308.546472	22.50003517	Q4	SIM2	-1372	0.167969681	0.757515471
chr21	11.5	41767811.5	271.4445258	11.94845629	Q4	RIPK4	-760.5	1.121535662	0.025643517
chr3	28.5	12553905.5	128.3637289	17.12346933	Q3	MKRN2	3181.5	9.079520841	7.90533443
chr3	46.5	27713635.5	149.9627574	1.587609881	Q3	EOMES	8686.5	0.419713907	0
chr3	160.5	27724766.5	247.6106083	1.486590949	Q4	EOMES	-2054.5	0.419713907	0
chr3	97.5	33217396.5	190.6690305	17.99473166	Q4	SUSD5	1200.5	1.275691073	14.77752692
chr3	1.5	62376077.5	199.2493457	10.44058941	Q4	FEZF2	-2528.5	0.125708093	0
chr3	279.5	71783809.5	140.9554468	28.35851396	Q3	PROK2	613.5	0.769211954	0
chr3	0.5	75656382.5	198.8010271	4.808802299	Q4	FRG2C	7945.5	0.041701584	0.056065329
chr3	5.5	99875542.5	114.5639001	13.59482937	Q3	FILIP1L	629.5	0.146306206	31.9637657
chr3	49.5	119033824.5	122.0270928	16.60082866	Q3	IGSF11	847.5	0.915883375	0.011194103
chr3	27	128492093	358.9122964	30.61782485	Q4	GATA2	1107	0.380647714	1.371723773
chr3	36.5	137766173.5	187.421858	6.801728229	Q4	SOX14	-1858.5	0	0
chr3	45.5	137772416.5	265.9097788	9.902046493	Q4	SOX14	-8101.5	0	0
chr3	3.5	138944167.5	325.0826187	38.45484241	Q4	FOXL2	2968.5	0.4354749	0.102116933
chr3	38	147354487	224.5680514	55.57582666	Q4	ZIC4	49796	0.026388032	0
chr3	8	147370140	297.4804117	2.519159077	Q4	ZIC4	34143	0.026388032	0
chr3	11	147388728	181.9381742	7.209125971	Q3	ZIC4	15555	0.026388032	0
chr3	54	147408029	252.6472225	14.01938693	Q4	ZIC4	-554	0.026388032	0
chr3	197	147422533	274.4072049	36.42279828	Q4	ZIC1	-13168	0.141184295	0
chr3	48.5	158096129.5	426.435751	52.98616122	Q4	SHOX2	9163.5	0	0.222945229
chr3	91.5	158102084.5	237.5349162	3.620143322	Q4	SHOX2	3208.5	0	0.222945229
chr3	50.5	158108221.5	272.5122892	2.938724898	Q4	SHOX2	-1802.5	0	0.222945229
chr3	13.5	169145442.5	183.6636678	15.25278248	Q3	MECOM	79.5	0.046361271	4.070149316
chr3	19	169660355	459.7638837	43.48479102	Q4	MECOM	3356	0.046361271	4.070149316
chr3	146	169667175	327.1592105	27.35299035	Q4	MECOM	-3395	0.046361271	4.070149316
chr3	3.5	185364167.5	135.618158	28.64152653	Q3	MAP3K13	-985.5	1.287399752	1.20976661
chr4	64	4855844	142.3454354	5.522686194	Q3	MSX1	3821	0.106592687	1.816935706
chr4	39	4861509	299.3365827	7.640134566	Q4	MSX1	-1844	0.106592687	1.816935706
chr4	198	20255914	211.2524276	33.15165008	Q4	SLIT2	-1652	0.548739872	3.335694602
chr4	139	41750047	243.5254673	4.536562874	Q4	PHOX2B	-1323	0	0
chr4	83	41873126	120.2277449	9.37500215	Q3	PHOX2B	-124402	0	0
chr4	53.5	54102784.5	146.0791392	2.758010283	Q3	GSX2	-2621.5	0	0

chr4	93.5	84492010.5	231.5733627	39.90666739	Q4	NKX6-1	7280.5	0.346554176	4.596254872
chr4	27.5	84494289.5	230.5311845	7.065748304	Q4	NKX6-1	5001.5	0.346554176	4.596254872
chr4	234.5	88458512.5	121.8312442	6.327399461	Q3	HERC5	-1393.5	2.112926418	0.39897548
chr4	4.5	93830978.5	307.9153913	8.690596436	Q4	ATOH1	-2225.5	0.277384056	0
chr4	83	108171156	283.6622171	41.60804797	Q4	LEF1	-2225	0.404333626	0.282625351
chr4	234	110616791	232.4107432	16.325559	Q4	PITX2	5376	0.141057172	1.930295624
chr4	162.5	110623839.5	191.2488034	25.3573561	Q4	PITX2	-763.5	0.141057172	1.930295624
chr4	202.5	110635290.5	268.677515	20.66343624	Q4	PITX2	6831.5	0.141057172	1.930295624
chr4	38.5	110638815.5	263.0176704	13.92038527	Q4	PITX2	3306.5	0.141057172	1.930295624
chr4	17.5	112513290.5	167.4599659	10.05938639	Q3	NEUROG	2888.5	0.025834195	0
chr4	145.5	146641023.5	118.9772925	7.73699011	Q3	POU4F2	-2130.5	0	0
chr4	59.5	150580759.5	192.7551251	3.995513355	Q4	LRBA	-716.5	3.01056269	6.170230523
chr4	52	150584767	201.5411951	19.75825765	Q4	LRBA	-1799	3.01056269	6.170230523
chr4	8	173519834	133.6186522	9.79223308	Q3	SCRG1	-691	0	0.729370943
chr4	42.5	173526620.5	240.2927119	42.45921045	Q4	HAND2	3607.5	0.062376271	0.18868777
chr4	35	173539305	167.3557064	3.402271913	Q3	HAND2	-9077	0.062376271	0.18868777
chr5	25.5	3597692.5	203.374575	12.87068738	Q4	IRX1	-1837.5	0.699581101	0
chr5	29.5	16935011.5	117.8165451	0.956112909	Q3	MYO10	1263.5	14.5937325	12.82615775
chr5	37.5	50968337.5	232.0380385	8.39471179	Q4	ISL1	415110.5	0.017758582	0
chr5	56	51382129	136.3853969	4.408593861	Q3	ISL1	1319	0.017758582	0
chr5	163.5	51385138.5	213.7465251	5.025617824	Q4	ISL1	-395.5	0.017758582	0
chr5	49	55174916	156.6582433	11.17121676	Q3	CDC20B	-1740	0	0
chr5	116	73445436	148.2525351	13.95675228	Q3	FOXD1	3340	1.774824544	28.64687845
chr5	130.5	73450472.5	173.3049202	22.72349489	Q3	FOXD1	-1696.5	1.774824544	28.64687845
chr5	212	78016418	203.4549446	47.44716937	Q4	TBCA	-240080	1.557352207	1.754951339
chr5	27.5	88143301.5	278.8701368	26.54460728	Q4	TMEM16	103079.5	0.927419483	1.454671168
chr5	100	88663550	195.9829102	15.83868876	Q4	MEF2C	121960	0.057569547	1.071145124
chr5	114.5	93573724.5	125.7357135	6.241089581	Q3	NR2F1	9497.5	0.031696783	32.41362557
chr5	210.5	93586955.5	223.6113205	0.936978695	Q4	NR2F1	145.5	0.031696783	32.41362557
chr5	78	93594224	152.5426112	18.96888007	Q3	NR2F1	-7123	0.031696783	32.41362557
chr5	130	123091937	363.0315553	22.52758192	Q4	PRDM6	6556	0.093593151	0.047663065
chr5	134.5	135032436.5	206.3133858	14.61676597	Q4	PITX1	1790.5	0.148760732	6.135418585
chr5	35	140110173	408.8013927	44.26644716	Q4	NRG2	-66499	0.024597337	0.023621196
chr5	26.5	146345338.5	115.810202	12.70942452	Q3	POU4F3	-6499.5	0.049985452	0.084003128
chr5	26	154474882	242.0509157	7.755218705	Q4	HAND1	3326	0.093485449	0
chr5	65	154483799	170.8474773	15.97412175	Q3	HAND1	-5573	0.093485449	0
chr5	7	173243597	113.1596188	11.0277833	Q3	NKX2-5	-8277	1.551639163	0
chr6	98.5	1379216.5	127.6831002	11.07651154	Q3	FOXF2	10359.5	0.16209274	13.62026032
chr6	8.5	1612667.5	210.6453742	10.28482047	Q4	FOXC1	-2752.5	0.38937827	18.69629302
chr6	15	5998711	168.0660579	3.445476259	Q3	NRN1	5313	0	0
chr6	44	10384540	141.9631122	28.96695378	Q3	TFAP2A	27850	0.058000135	2.57759861
chr6	0	10410677	163.9671177	45.91659725	Q3	TFAP2A	1713	0.058000135	2.57759861
chr6	101	27287814	111.7384996	6.312192548	Q3	LOC1053	24355	0	0
chr6	223.5	27558400.5	111.0012066	13.90816138	Q3	ZNF184	-85296.5	2.458208439	3.100161924
chr6	42	28208542	165.9116888	1.958168492	Q3	ZSCAN9	16160	2.143249383	2.587936994
chr6	92	50819359	123.3606231	0.104813121	Q3	TFAP2B	-488	0	0
chr6	87	50847102	242.6474105	16.65492205	Q4	TFAP2B	-28231	0	0
chr6	267	84771981	222.7238898	8.283799639	Q4	NT5E	678102	0.021785695	270.312372
chr6	73.5	98826339.5	208.6502207	27.7838616	Q4	POU3F2	8234.5	0.299967747	0.012147259
chr6	29.5	98837095.5	258.6875576	7.785271994	Q4	POU3F2	-2521.5	0.299967747	0.012147259

chr6	37.5	98842210.5	186.6593232	29.61972665	Q4	POU3F2	-7636.5	0.299967747	0.012147259
chr6	183	100456626	110.0263887	1.886820882	Q3	SIM1	7829	0.091047469	0
chr6	210.5	100462079.5	246.339777	13.58785468	Q4	SIM1	2375.5	0.091047469	0
chr6	25	105995698	183.3625897	36.79244271	Q3	PRDM1	51031	0.113426885	1.506803555
chr6	43	111480402	161.917785	1.258864903	Q3	REV3L	-880	0.976210789	14.28239599
chr6	267	117269603	122.6369833	7.326918412	Q3	VGLL2	-3862	0	0
chr6	79	133243856	224.8470767	29.30439879	Q4	EYA4	-2203	0.020028069	4.047398864
chr6	62	133892190	236.964689	6.639976089	Q4	TCF21	-3077	0.105072018	16.47617364
chr6	68.5	137487159.5	128.9903414	8.412768693	Q3	OLIG3	7233.5	0.659166072	0
chr6	81	137492773	142.5922742	25.6247884	Q3	OLIG3	1620	0.659166072	0
chr6	37.5	137495276.5	162.9500655	32.71736666	Q3	OLIG3	-883.5	0.659166072	0
chr6	102	137498913	177.3688282	5.663514287	Q3	OLIG3	-4520	0.659166072	0
chr6	78.5	165665693.5	134.4033601	13.46480444	Q3	PDE10A	-2453.5	0.495943713	0.166010351
chr7	164.5	1228611.5	448.9956696	9.84841238	Q4	UNCX	4260.5	0	0
chr7	39	1237372	238.3775546	43.79298541	Q4	UNCX	-4500	0	0
chr7	115.5	8440680.5	155.6378862	16.64881012	Q3	NXPH1	-7071.5	0.029143113	0
chr7	179	17300138	113.714403	11.19496496	Q3	AHR	-1486	1.073458979	18.989716
chr7	241	19113161	234.0157575	13.07728771	Q4	TWIST1	4474	1.332387525	5.41241407
chr7	29.5	20792936.5	340.4743022	12.95494991	Q4	SP8	-6051.5	14.28805695	0
chr7	18	25853592	327.1452612	9.094998312	Q4	NFE2L3	298606	39.12185929	4.200699688
chr7	63.5	25855992.5	375.6489744	3.808390626	Q4	NFE2L3	296205.5	39.12185929	4.200699688
chr7	27.5	25860431.5	508.8229992	85.76105487	Q4	NFE2L3	291766.5	39.12185929	4.200699688
chr7	82.5	25863312.5	623.0703783	137.2176355	Q4	NFE2L3	288885.5	39.12185929	4.200699688
chr7	139.5	26857181.5	343.5067709	19.66953561	Q4	SKAP2	540.5	0.442671441	5.184277356
chr7	44	26863070	198.9360437	11.53297655	Q4	SKAP2	-5348	0.442671441	5.184277356
chr7	149.5	27100511.5	395.8291543	1.643192555	Q4	HOXA2	2170.5	0.008307481	2.038327878
chr7	24.5	27112821.5	313.3418486	3.264310894	Q4	HOXA3	1019.5	0.008724572	2.62451475
chr7	148	27187422	150.7302625	7.409219488	Q3	HOXA11	-2191	0	0
chr7	43	27194012	238.1612498	21.91624497	Q4	HOXA13	6078	0.012038061	0
chr7	45	27197763	271.3154279	18.21469332	Q4	HOXA13	2327	0.012038061	0
chr7	110.5	27206016.5	205.8174291	21.70188023	Q4	HOXA13	-5926.5	0.012038061	0
chr7	25.5	27225083.5	257.6292541	1.673035593	Q4	EVX1	17718.5	0.138692028	0
chr7	59.5	27242269.5	237.8332431	8.375920984	Q4	EVX1	532.5	0.138692028	0
chr7	173.5	27251176.5	262.1000147	6.504607499	Q4	EVX1	-8374.5	0.138692028	0
chr7	109	35252878	287.7901032	6.20212162	Q4	TBX20	1221	0	0
chr7	167	65510532	242.644861	54.46658872	Q4	ZNF92	-136677	16.44727718	3.669217435
chr7	182.5	92837881.5	205.9243969	12.1259519	Q4	CDK6	-1309.5	3.016389789	26.47287132
chr7	25	96992135	149.6909858	7.091908684	Q3	DLX6	13418	0.126043315	0
chr7	113.5	96997728.5	160.8535411	5.619288519	Q3	DLX6	7824.5	0.126043315	0
chr7	211.5	97004252.5	208.0084742	6.070547195	Q4	DLX6	1300.5	0.126043315	0
chr7	90	114082719	137.6027658	24.60643574	Q3	FOXP2	3608	0.104429157	0.506245909
chr7	21.5	122302759.5	351.3101512	18.36444905	Q4	FEZF1	1750.5	0.373050823	0
chr7	44	123533327	145.7318609	14.9179084	Q3	IQUB	-135	0.014744897	0.03716934
chr7	116	129783591	204.6307745	27.49902482	Q4	NRF1	-153523	5.019558547	1.922381908
chr7	37.5	130735234.5	286.2675186	49.83335057	Q4	KLF14	-1028.5	0.039920813	0.033544492
chr7	66.5	136871714.5	117.0963476	10.70114142	Q3	CHRM2	-2468.5	0	27.90503018
chr7	189	155377229	157.4982591	5.873835899	Q3	EN2	80900	0.026104332	0.014623225
chr7	70	155456639	154.9668332	29.33549937	Q3	EN2	1490	0.026104332	0.014623225
chr7	80	155465316	207.5656065	2.511845369	Q4	EN2	-7187	0.026104332	0.014623225
chr7	143	155511707	339.3022794	35.42642543	Q4	CNPY1	-1546	0	0

chr7	73.5	157004775.5	119.8475548	3.602034942	Q3	MNX1	4658.5	0	0.018992291
chr7	172.5	157020336.5	256.3958456	19.57318223	Q4	MNX1	-9674.5	0	0.018992291
chr7	109.5	157687843.5	290.2048869	13.67092408	Q4	DNAJB6	-350295.5	16.05025301	8.647273618
chr8	89	26048913	317.8604619	20.76737375	Q4	EBF2	-3501	0.090952592	1.48774506
chr8	26	53876549	142.9896026	10.49882035	Q3	RGS20	4323	0.111133865	2.76753535
chr8	153	54456544	320.1359412	14.36012832	Q4	SOX17	1391	0	0
chr8	232.5	54465289.5	277.9172988	25.57090969	Q4	LOC1001	5135.5	0	0
chr8	212.5	60654492.5	156.243755	28.44618014	Q3	CHD7	24247.5	10.12885995	0.193348123
chr8	170.5	64367753.5	204.0045998	28.55349986	Q4	BHLHE22	212611.5	0.217408021	0
chr8	68.5	64579144.5	167.122083	4.718929999	Q3	BHLHE22	1220.5	0.217408021	0
chr8	33.5	66960645.5	182.9914216	28.92170209	Q3	TCF24	1944.5	0.590828039	0.101318059
chr8	6	70072730	154.9738723	38.88864518	Q3	PRDM14	-1479	37.95871657	0
chr8	42.5	71555286.5	301.9878913	30.86387392	Q4	EYA1	-7183.5	0.453476181	0.015713198
chr8	106	71559125	141.8896099	4.680365429	Q3	EYA1	-11022	0.453476181	0.015713198
chr8	0.5	76675148.5	260.0047908	28.8032787	Q4	ZFHX4	6070.5	0.088079343	9.070642888
chr8	113	76684247	114.9016542	1.183529963	Q3	ZFHX4	-466	0.088079343	9.070642888
chr8	49	96155799	119.6556721	7.058782214	Q3	GDF6	5006	0.094486781	0.044313462
chr8	140	98943207	236.4976011	14.40468457	Q4	STK3	-598	0.646709048	1.432253814
chr8	4	98946242	238.4198404	16.03743883	Q4	OSR2	-1797	0.003435446	0.060621167
chr9	6.5	112805.5	197.6710884	36.62469445	Q4	FOXD4	5610.5	0	0
chr9	5	114896	124.2136913	5.027815345	Q3	FOXD4	3520	0	0
chr9	6.5	116091.5	113.15755	0.560574274	Q3	FOXD4	2324.5	0	0
chr9	17	843539	277.4582627	18.93113527	Q4	DMRT1	-243	0.080030889	0
chr9	157.5	969009.5	124.2119014	5.439243411	Q3	DMRT3	7645.5	0.232426451	0
chr9	148.5	2013884.5	117.6797472	2.175859608	Q3	DMRT2	-960898.5	0.119322114	0
chr9	204	2019893	158.0841525	32.89041558	Q3	SMARCA2	2052	1.595673228	5.774114264
chr9	62.5	14349618.5	203.5990906	32.86276156	Q4	NFIB	-27232.5	0.590890238	0.287453594
chr9	128.5	17580408.5	128.5823685	24.12500351	Q3	SH3GL2	-1342.5	0.844762692	0.051006009
chr9	258.5	21973193.5	261.9918409	4.433741258	Q4	CDKN2A	1662.5	0.044215868	1.215138755
chr9	120	21989129	195.9027853	32.00416953	Q4	CDKN2A	5262	0.044215868	1.215138755
chr9	21	23823870	117.250125	6.780847174	Q3	ELAVL2	-739	1.526871275	0.027591246
chr9	7	23830796	127.6222253	14.62589523	Q3	ELAVL2	-4557	1.526871275	0.027591246
chr9	6	64775626	122.4434481	25.93187111	Q3	LOC1079	-55745	NA	NA
chr9	27	74501574	118.2175519	18.60511151	Q3	RORB	-4239	0.270495634	0
chr9	11	77013820	128.9845385	2.992221688	Q3	FOXB2	5835	0.795957712	0
chr9	13	77021540	283.6881542	4.036966494	Q4	FOXB2	-1885	0.795957712	0
chr9	141	84140958	166.3168675	22.13082001	Q3	RMI1	-145908	19.76728112	4.195032556
chr9	135	90802635	134.6660024	13.34824808	Q3	SYK	-703	0.114713284	0
chr9	14	91422610	148.9479243	12.48615799	Q3	NFIL3	-482	3.483257919	15.02946546
chr9	86.5	97851389.5	276.7267512	21.10666437	Q4	FOXE1	1836.5	0.153639189	0.186476751
chr9	18.5	97982130.5	135.2525782	11.06509029	Q3	HEMGN	-37279.5	0	0
chr9	76.5	105693400.5	151.4106465	2.379674341	Q3	TMEM38	1140.5	5.393795297	2.220117735
chr9	99.5	122220449.5	193.0503917	43.05039167	Q4	LHX6	1289.5	0.783996846	0
chr9	47.5	124016558.5	152.9670399	13.8197827	Q3	LHX2	-4790.5	2.920668576	0
chr9	58	124768783	214.6337326	4.164712338	Q4	NR6A1	2475	27.31886722	0.041591049
chr9	156	126618482	212.7304343	20.4823138	Q4	LMX1B	-4554	0.020268543	0.025546749
chr9	162	126625016	228.7418072	19.6333403	Q4	LMX1B	-11088	0.020268543	0.025546749
chr9	167	128392216	154.944789	10.75874146	Q3	CERCAM	13778	0.256348706	4.270610654
chrX	51.5	25003876.5	190.2621049	12.64484986	Q4	ARX	12087.5	0	0
chrX	53	25014819	144.839417	52.05984838	Q3	ARX	1145	0	0

chrX	51.5	104253456.5	127.4535071	11.17450019	Q3	ESX1	1475.5	0	0

Table S7. List of genes with high-confidence PTE

Gene	Phenotype (OMIM number)	Gene transcription in NT2-D1 (RPKM)	Transcription quartile in NT2-D1	Gene transcription in TIG-3 (RPKM)	Transcription quartile in TIG-3	Number of PTEs
NR2F2	46,XX sex reversal 5 (618901); Congenital heart defects, multiple types,	0.033844487	Q1	51.01496924	Q4	5
MEIS1	no assosiated desease in OMIM	0.041212893	Q1	25.39085964	Q4	5
TBX5	Holt-Oram syndrome (142900)	0.406346657	Q2	20.95892496	Q4	4
NFE2L3	no assosiated desease in OMIM	39.12185929	Q4	4.200699688	Q3	4
PAX6	Aniridia (106210); Anterior segment dysgenesis 5, multiple subtypes (604229); Cataract with late-onset corneal dystrophy (106210); Foveal hypoplasia 1 (136520); Keratitis (148190); Optic nerve hypoplasia	0.1673198	Q2	0.073353796	Q2	4
OLIG3	no assosiated desease in OMIM	0.659166072	Q2	0	Q1	4
IGF1R	Insulin-like growth factor I, resistance to (270450)	7.023642028	Q4	10.85509799	Q4	4
PITX2	Anterior segment dysgenesis 4 (137600); Axenfeld-Rieger syndrome, type 1 (180500); Ring dermoid of cornea (180550)	0.141057172	Q2	1.930295624	Q3	4
HOXA13	Hand-foot-uterus syndrome (140000)	0.012038061	Q1	0	Q1	4
HAND2	no assosiated desease in OMIM	0.062376271	Q1	0.18868777	Q2	4
ZIC4	no assosiated desease in OMIM	0.026388032	Q1	0	Q1	4
IGF2BP1	no assosiated desease in OMIM	54.48932942	Q4	9.092515856	Q4	3
SPAG6	no assosiated desease in OMIM	0.0745995	Q2	0	Q1	3
MECOM	Radioulnar synostosis with amegakaryocytic thrombocytopenia 2 (616738)	0.046361271	Q1	4.070149316	Q3	3
SHOX2	no assosiated desease in OMIM	0	Q1	0.222945229	Q2	3
ZFHX4	no assosiated desease in OMIM	0.088079343	Q2	9.070642888	Q4	3
EN2	no assosiated desease in OMIM	0.026104332	Q1	0.014623225	Q1	3
FOXD4	no assosiated desease in OMIM	0	Q1	0	Q1	3
CDKN2A	{Melanoma and neural system tumor syndrome} (155755); {Melanoma, cutaneous malignant, 2} (155601); {Melanoma-pancreatic cancer syndrome} (606719)	0.044215868	Q1	1.215138755	Q2	3
EBF3	Hypotonia, ataxia, and delayed development syndrome (617330)	0.0794749	Q2	1.270464636	Q2	3
DLX6	no assosiated desease in OMIM	0.126043315	Q2	0	Q1	3
ISL1	no assosiated desease in OMIM	0.017758582	Q1	0	Q1	3
NR2F1	Bosch-Boonstra-Schaaf optic atrophy syndrome (615722)	0.031696783	Q1	32.41362557	Q4	3
ISL2	no assosiated desease in OMIM	0.028269284	Q1	0.35630992	Q2	3
TFAP2A	Branchiooculofacial syndrome (113620)	0.058000135	Q1	2.57759861	Q3	3
MEIS2	Cleft palate, cardiac defects, and mental retardation (600987)	0.225740902	Q2	8.645871414	Q4	3
GATA6	Atrial septal defect 9 (614475); Atrioventricular septal defect 5 (614474); Pancreatic agenesis and congenital heart defects (600001); Persistent truncus arteriosus (217095); Tetralogy of Fallot (187500)	0.140822361	Q2	0.407579797	Q2	3
POU3F2	no assosiated desease in OMIM	0.299967747	Q2	0.012147259	Q1	3
EMX1	no assosiated desease in OMIM	0.083486021	Q2	0	Q1	3

BARHL2	no assosiated desease in OMIM	0	Q1	0	Q1	3
EVX1	no assosiated desease in OMIM	0.138692028	Q2	0	Q1	3
DLX1	no assosiated desease in OMIM	0.049586911	Q1	0.208333398	Q2	3
FOXB1	no entry in OMIM	0.749165094	Q2	0	Q1	3
SIM1	no assosiated desease in OMIM	0.091047469	Q2	0	Q1	3
SIX3	Holoprosencephaly 2 (157170); Schizencephaly (269160)	0.126912831	Q2	0	Q1	3
SP9	no assosiated desease in OMIM	0	Q1	0	Q1	3
LMX1B	Focal segmental glomerulosclerosis 10 (256020); Nail-patella syndrome	0.020268543	Q1	0.025546749	Q2	3
LOC10050962(	no entry in OMIM	0	Q1	0	Q1	3
EN1	no assosiated desease in OMIM	0.009973465	Q1	0	Q1	3
LHX9	no assosiated desease in OMIM	0	Q1	0	Q1	3
SOX1	no assosiated desease in OMIM	0.050338319	Q1	0	Q1	3
FOXG1	Rett syndrome, congenital variant (613454)	0.055286467	Q1	3.406764425	Q3	3
OTX1	no assosiated desease in OMIM	0	Q1	0	Q1	3
SIX1	Branchiootic syndrome 3 (608389); Deafness, autosomal dominant 23 (605192)	0.032982585	Q1	35.30823117	Q4	2
PHOX2B	Central hypoventilation syndrome, congenital, 1, with or without Hirschsprung disease (209880); Neuroblastoma with Hirschsprung disease (613013); {Neuroblastoma, susceptibility to, 2} (613013)	0	Q1	0	Q1	2
SOX14	no assosiated desease in OMIM	0	Q1	0	Q1	2
MSX1	Ectodermal dysplasia 3, Witkop type (189500); Orofacial cleft 5 (608874); Tooth agenesis, selective, 1, with or without orofacial cleft (106600)	0.106592687	Q2	1.816935706	Q3	2
TTC6	no entry in OMIM	0.004192649	Q1	0.049321735	Q2	2
GSX1	no assosiated desease in OMIM	0	Q1	0	Q1	2
SKAP1	no assosiated desease in OMIM	0.01044604	Q1	0.017555109	Q1	2
ANP32A	no assosiated desease in OMIM	42.48597246	Q4	7.707482818	Q4	2
FOXB2	no entry in OMIM	0.795957712	Q2	0	Q1	2
HOXD8	no assosiated desease in OMIM	0.022473489	Q1	0	Q1	2
ARX	Developmental and epileptic encephalopathy 1 (308350); Hydranencephaly with abnormal genitalia (300215); Intellectual developmental disorder, X-linked 29 (300419); Lissencephaly, X-linked 2 (300215); Partington syndrome (309510); Proud syndrome (300004)	0	Q1	0	Q1	2
SATB2	Glass syndrome (612313)	2.281157995	Q3	1.698518324	Q3	2
NFIX	Malan syndrome (614753); Marshall-Smith syndrome (602535)	0.087207091	Q2	11.73547261	Q4	2
PAX3	Craniofacial-deafness-hand syndrome (122880); Rhabdomyosarcoma 2, alveolar (268220); Waardenburg syndrome, type 1 (193500); Waardenburg syndrome, type 3	0.076992427	Q2	0	Q1	2
NKX2-4	no assosiated desease in OMIM	0.474414114	Q2	0	Q1	2
OLIG2	no assosiated desease in OMIM	1.337906344	Q2	0	Q1	2
IRX5	Hamamy syndrome (611174)	0.101829941	Q2	0.018013733	Q1	2

RUNX1	Leukemia, acute myeloid (601626); Platelet disorder, familial, with associated myeloid malignancy (601399)	0.448003905	Q2	8.505441709	Q4	2
SIM2	no assosiated desease in OMIM	0.167969681	Q2	0.757515471	Q2	2
ELAVL2	no assosiated desease in OMIM	1.526871275	Q3	0.027591246	Q2	2
EOMES	no assosiated desease in OMIM	0.419713907	Q2	0	Q1	2
FOXF1	Alveolar capillary dysplasia with misalignment of pulmonary veins (265380)	0.023061204	Q1	26.02434622	Q4	2
KCTD1	Scalp-ear-nipple syndrome (181270)	1.2399146	Q2	1.854822445	Q3	2
SHCBP1L	no assosiated desease in OMIM	0	Q1	0.020991902	Q1	2
POU3F3	Snijders Blok-Fisher syndrome (618604)	0	Q1	0	Q1	2
NKX6-1	no assosiated desease in OMIM	0.346554176	Q2	4.596254872	Q3	2
HLX	no assosiated desease in OMIM	0.129624405	Q2	1.165446649	Q2	2
MEF2C	Chromosome 5q14.3 deletion syndrome (613443); Neurodevelopmental disorder with hypotonia, stereotypic hand movements, and impaired language (613443)	0.057569547	Q1	1.071145124	Q2	2
HMX2	no assosiated desease in OMIM	0	Q1	0	Q1	2
EMX2	Schizencephaly (269160)	0.051003802	Q1	0	Q1	2
EYA1	Anterior segment anomalies with or without cataract (602588); Branchiootic syndrome 1 (602588); Branchiootorenal syndrome 1, with or without cataracts (113650)	0.453476181	Q2	0.015713198	Q1	2
LBX1	no assosiated desease in OMIM	0	Q1	0	Q1	2
HAND1	no assosiated desease in OMIM	0.093485449	Q2	0	Q1	2
NRF1	no assosiated desease in OMIM	5.019558547	Q3	1.922381908	Q3	2
GATA3	Hypoparathyroidism, sensorineural deafness, and renal dysplasia (146255)	0.985305173	Q2	0.074588114	Q2	2
PROX1	no assosiated desease in OMIM	0.411385571	Q2	0.035454085	Q2	2
FOXD1	no assosiated desease in OMIM	1.774824544	Q3	28.64687845	Q4	2
CRYBG1	no assosiated desease in OMIM	0.736596636	Q2	2.718616634	Q3	2
TFAP2B	Char syndrome (169100); Patent ductus arteriosus 2 (617035)	0	Q1	0	Q1	2
LHX8	no assosiated desease in OMIM	0.045483298	Q1	0	Q1	2
HOXA11	Radioulnar synostosis with amegakaryocytic thrombocytopenia 1 (605432)	0	Q1	0	Q1	2
UBE2W	no assosiated desease in OMIM	2.325800295	Q3	2.684962006	Q3	2
DMRTA2	no assosiated desease in OMIM	0	Q1	0	Q1	2
SKAP2	no assosiated desease in OMIM	0.442671441	Q2	5.184277356	Q4	2
UNCX	no entry in OMIM	0	Q1	0	Q1	2
MNX1	Currarino syndrome (176450)	0	Q1	0.018992291	Q1	2
VAX1	no assosiated desease in OMIM	0.01020781	Q1	0	Q1	2
BSX	no assosiated desease in OMIM	0.042081769	Q1	0	Q1	2
PTHLH	Brachydactyly, type E2 (613382)	0	Q1	0.212161747	Q2	2
FOXD4L1	no assosiated desease in OMIM	0	Q1	0	Q1	2
BHLHE22	no assosiated desease in OMIM	0.217408021	Q2	0	Q1	2
LRBA	Immunodeficiency, common variable, 8, with autoimmunity (614700)	3.01056269	Q3	6.170230523	Q4	2
HOXC4	no assosiated desease in OMIM	0	Q1	0	Q1	2
CNPY1	no assosiated desease in OMIM	0	Q1	0	Q1	1
STK3	no assosiated desease in OMIM	0.646709048	Q2	1.432253814	Q3	1

LOC10012909[illegible]	no entry in OMIM	0	Q1	0	Q1	1
SMARCA2	Blepharophimosis-impaired intellectual development syndrome (619293); Nicolaides-Baraitser syndrome (601358)	1.595673228	Q3	5.774114264	Q4	1
ZNF92	no assosiated desease in OMIM	16.44727718	Q4	3.669217435	Q3	1
FOXE1	Bamforth-Lazarus syndrome (241850); {Thyroid cancer, nonmedullary, 4} (616534)	0.153639189	Q2	0.186476751	Q2	1
CHD7	CHARGE syndrome (214800); Hypogonadotropic hypogonadism 5 with or without anosmia (612370)	10.12885995	Q4	0.193348123	Q2	1
TBX20	Atrial septal defect 4 (611363)	0	Q1	0	Q1	1
SH3GL2	no assosiated desease in OMIM	0.844762692	Q2	0.051006009	Q2	1
NFIL3	no assosiated desease in OMIM	3.483257919	Q3	15.02946546	Q4	1
TCF24	no entry in OMIM	0.590828039	Q2	0.101318059	Q2	1
HOXA3	no assosiated desease in OMIM	0.008724572	Q1	2.62451475	Q3	1
HOXA2	Microtia with or without hearing impairment (AD) (612290)	0.008307481	Q1	2.038327878	Q3	1
PRDM14	no assosiated desease in OMIM	37.95871657	Q4	0	Q1	1
GDF6	Klippel-Feil syndrome 1, autosomal dominant (118100); Leber congenital amaurosis 17 (615360); Microphthalmia with coloboma 6, digenic (613703); Microphthalmia, isolated 4 (613094); Multiple synostoses syndrome 4	0.094486781	Q2	0.044313462	Q2	1
DNAJB6	Muscular dystrophy, limb-girdle, autosomal dominant 1 (603511)	16.05025301	Q4	8.647273618	Q4	1
CDK6	no assosiated desease in OMIM	3.016389789	Q3	26.47287132	Q4	1
OSR2	no assosiated desease in OMIM	0.003435446	Q1	0.060621167	Q2	1
EBF2	no assosiated desease in OMIM	0.090952592	Q2	1.48774506	Q3	1
RGS20	no assosiated desease in OMIM	0.111133865	Q2	2.76753535	Q3	1
DMRT3	no assosiated desease in OMIM	0.232426451	Q2	0	Q1	1
DMRT1	no assosiated desease in OMIM	0.080030889	Q2	0	Q1	1
CHRM2	no assosiated desease in OMIM	0	Q1	27.90503018	Q4	1
DMRT2	no assosiated desease in OMIM	0.119322114	Q2	0	Q1	1
KLF14	no assosiated desease in OMIM	0.039920813	Q1	0.033544492	Q2	1
LOC10798706[illegible]	no entry in OMIM		Q0		Q0	1
SOX17	Vesicoureteral reflux 3 (613674)	0	Q1	0	Q1	1
SYK	Immunodeficiency 82 with systemic inflammation (619381)	0.114713284	Q2	0	Q1	1
IQUB	no entry in OMIM	0.014744897	Q1	0.03716934	Q2	1
RORB	{Epilepsy, idiopathic generalized, susceptibility to, 15} (618357)	0.270495634	Q2	0	Q1	1
FEZF1	Hypogonadotropic hypogonadism 22, with or without anosmia (616030)	0.373050823	Q2	0	Q1	1
FOXP2	Speech-language disorder-1 (602081)	0.104429157	Q2	0.506245909	Q2	1
NFIB	Macrocephaly, acquired, with impaired intellectual development (618286)	0.590890238	Q2	0.287453594	Q2	1
RMI1	no assosiated desease in OMIM	19.76728112	Q4	4.195032556	Q3	1
TP73	Ciliary dyskinesia, primary, 47, and lissencephaly (619466)	0.347265224	Q2	0.053459277	Q2	1
ALX1	Frontonasal dysplasia 3 (613456)	2.464717682	Q3	0.147931612	Q2	1
HEMGN	no assosiated desease in OMIM	0	Q1	0	Q1	1
KRT27	no assosiated desease in OMIM	0	Q1	0	Q1	1
IGF2BP2	{Diabetes mellitus, noninsulin-dependent, susceptibility to} (125853)	10.3076335	Q4	22.14604332	Q4	1
PFN2	no assosiated desease in OMIM	68.20290515	Q4	48.70290776	Q4	1

RAD21L1	no assosiated desease in OMIM	0.02024308	Q1	0	Q1	1
CHN1	Duane retraction syndrome 2 (604356)	10.10146373	Q4	10.86659139	Q4	1
SEPTIN10	no assosiated desease in OMIM	12.30032317	Q4	7.791173985	Q4	1
HNRNPLL	no assosiated desease in OMIM	11.42394193	Q4	5.257245302	Q4	1
ANKRD29	no entry in OMIM	0.2188252	Q2	0.066863096	Q2	1
EMILIN2	no assosiated desease in OMIM	2.098302105	Q3	0.164492355	Q2	1
NRG4	no assosiated desease in OMIM	0.47777666	Q2	0.452353976	Q2	1
TMEM38B	Osteogenesis imperfecta, type XIV (615066)	5.393795297	Q3	2.220117735	Q3	1
NEO1	no assosiated desease in OMIM	8.858428499	Q4	9.633978237	Q4	1
ONECUT1	no assosiated desease in OMIM	1.028933824	Q2	0	Q1	1
PTPN21	no assosiated desease in OMIM	1.269949962	Q2	7.572904868	Q4	1
ESR2	no assosiated desease in OMIM	0.147799384	Q2	0.105563406	Q2	1
SYNE2	Emery-Dreifuss muscular dystrophy 5, autosomal dominant (612999)	2.54131422	Q3	1.292667962	Q2	1
FERMT2	no assosiated desease in OMIM	17.16914526	Q4	50.32709549	Q4	1
NDFIP2	no assosiated desease in OMIM	4.474730845	Q3	4.962621435	Q4	1
RXFP2	no assosiated desease in OMIM	0	Q1	0	Q1	1
SMIM14	no entry in OMIM	3.629008427	Q3	6.531369171	Q4	1
ADGRV1	Usher syndrome, type 2C (605472); Usher syndrome, type 2C, GPR98/PDZD7 digenic (605472)	1.505110935	Q3	0.005094494	Q1	1
RGMB	no assosiated desease in OMIM	2.705338797	Q3	37.76404132	Q4	1
ZNF608	no entry in OMIM	8.397013639	Q4	4.885737253	Q4	1
NKAP	Intellectual developmental disorder, X-linked, syndromic, Hackman-Di Donato type (301039)	7.734815018	Q4	7.971525889	Q4	1
MAGED2	Bartter syndrome, type 5, antenatal, transient (300971)	39.82137697	Q4	32.89517376	Q4	1
PTCH1	Basal cell carcinoma, somatic (605462); Basal cell nevus syndrome (109400); Holoprosencephaly 7 (610828)	7.915354278	Q4	1.815858953	Q3	1
IFNA2	no assosiated desease in OMIM	0	Q1	0	Q1	1
EXT1	Chondrosarcoma (215300); Exostoses, multiple, type 1 (133700)	5.121212408	Q3	35.66588487	Q4	1
MMP16	no assosiated desease in OMIM	0.955122049	Q2	5.125357631	Q4	1
MOS	no assosiated desease in OMIM	0	Q1	0	Q1	1
TEX15	Spermatogenic failure 25 (617960)	2.172405911	Q3	0.131454145	Q2	1
EXTL3	Immunoskeletal dysplasia with neurodevelopmental abnormalities (617425)	17.79706383	Q4	5.778964741	Q4	1
MTUS1	no assosiated desease in OMIM	2.132564123	Q3	0.154400965	Q2	1
CACNA2D1	no assosiated desease in OMIM	2.779630153	Q3	4.401664853	Q3	1
FERD3L	no assosiated desease in OMIM	0	Q1	0	Q1	1
PHACTR2	no assosiated desease in OMIM	5.593405788	Q3	13.96035582	Q4	1
IL20RA	no assosiated desease in OMIM	0.280456347	Q2	0	Q1	1
MYO6	Deafness, autosomal dominant 22 (606346); Deafness, autosomal dominant 22, with hypertrophic cardiomyopathy (606346); Deafness, autosomal recessive 37 (607821)	2.562177882	Q3	3.940870943	Q3	1

RUNX2	Cleidocranial dysplasia (119600); Cleidocranial dysplasia, forme fruste, dental anomalies only (119600); Cleidocranial dysplasia, forme fruste, with brachydactyly (119600); Metaphyseal dysplasia with maxillary hypoplasia with or without	0.066995746	Q1	3.95940813	Q3	1
DSP	Arrhythmogenic right ventricular dysplasia 8 (607450); Cardiomyopathy, dilated, with woolly hair and keratoderma (605676); Dilated cardiomyopathy with woolly hair, keratoderma, and tooth agenesis (615821); Epidermolysis bullosa, lethal acantholytic (609638); Keratosis palmoplantaris striata II (612908); Skin	6.252507711	Q3	8.395989145	Q4	1
FLT1	no assosiated desease in OMIM	3.7826062	Q3	1.17411281	Q2	1
ARHGEF12	no assosiated desease in OMIM	4.808432565	Q3	18.39242067	Q4	1
SESN3	no assosiated desease in OMIM	4.98348262	Q3	0.473249142	Q2	1
TNFRSF11A	Osteolysis, familial expansile (174810); Osteopetrosis, autosomal recessive 7 (612301); {Paget disease of bone 2, early-onset} (602080)	0.902268363	Q2	0	Q1	1
HOXB13	{Prostate cancer, hereditary, 9} (610997)	0.087515674	Q2	0	Q1	1
HOXB8	no assosiated desease in OMIM	0	Q1	0.273599367	Q2	1
ISLR2	no assosiated desease in OMIM	0.205297391	Q2	0	Q1	1
ARHGEF7	no assosiated desease in OMIM	5.235939848	Q3	3.833140473	Q3	1
ZIC5	no assosiated desease in OMIM	9.269843875	Q4	0	Q1	1
TWIST1	Craniosynostosis 1 (123100); Robinow-Sorauf syndrome (180750); Saethre-Chotzen syndrome with or without eyelid anomalies (101400); Sweeney-Cox syndrome (617746)	1.332387525	Q2	5.41241407	Q4	1
BAMBI	no assosiated desease in OMIM	42.88986603	Q4	2.159767207	Q3	1
FMN2	Intellectual developmental disorder, autosomal recessive 47 (616193)	0.383916988	Q2	5.6184209	Q4	1
ATF3	no assosiated desease in OMIM	3.463010321	Q3	0.598142757	Q2	1
TBX15	Cousin syndrome (260660)	1.26781282	Q2	0.011454972	Q1	1
FNBP1L	no assosiated desease in OMIM	16.72605291	Q4	1.886754475	Q3	1
POU3F1	no assosiated desease in OMIM	9.549500318	Q4	0	Q1	1
ESX1	no entry in OMIM	0	Q1	0	Q1	1
CERCAM	no assosiated desease in OMIM	0.256348706	Q2	4.270610654	Q3	1
NR6A1	no assosiated desease in OMIM	27.31886722	Q4	0.041591049	Q2	1
LHX2	no assosiated desease in OMIM	2.920668576	Q3	0	Q1	1
LHX6	no assosiated desease in OMIM	0.783996846	Q2	0	Q1	1
CBX2	no assosiated desease in OMIM	13.99463242	Q4	0.275072529	Q2	1
TBR1	Intellectual developmental disorder with autism and speech delay (606053)	0.027816763	Q1	0.031165002	Q2	1
BTRC	no assosiated desease in OMIM	3.215059761	Q3	6.659083918	Q4	1
EVX2	no assosiated desease in OMIM	0.005793568	Q1	0	Q1	1
SFTPD	no assosiated desease in OMIM	0.151051726	Q2	0	Q1	1
ENAH	no assosiated desease in OMIM	28.55389171	Q4	26.68428128	Q4	1
GPR25	no assosiated desease in OMIM	0	Q1	0	Q1	1
S1PR1	no assosiated desease in OMIM	1.055741905	Q2	2.604372399	Q3	1
HFM1	Premature ovarian failure 9 (615724)	0.313496022	Q2	0.018815937	Q1	1
ERRFI1	no assosiated desease in OMIM	23.27573944	Q4	13.16909864	Q4	1

NR5A1	46, XX sex reversal 4 (617480); 46XY sex reversal 3 (612965); Adrenocortical insufficiency (612964); Premature ovarian failure 7 (612964); Spermatogenic failure 8 (613957)	0.074356505	Q2	0	Q1	1
NKX2-6	Conotruncal heart malformations (217095); Persistent truncus arteriosus (217095)	0	Q1	0	Q1	1
SNX10	Osteopetrosis, autosomal recessive 8 (615085)	7.190649812	Q4	0.029745865	Q2	1
LIN28B	no assosiated desease in OMIM	23.12700333	Q4	0	Q1	1
MAK	Retinitis pigmentosa 62 (614181)	0.183268063	Q2	1.646569552	Q3	1
BMPR1B	Acromesomelic dysplasia 3 (609441); Brachydactyly, type A1, D (616849); Brachydactyly, type A2 (112600)	0.450673525	Q2	1.21513729	Q2	1
NKX3-2	Spondylo-megaepiphyseal-metaphyseal dysplasia (613330)	4.23149934	Q3	0	Q1	1
SLC6A20	Hyperglycinuria (138500); Iminoglycinuria, digenic (242600)	0.368101007	Q2	0.01742571	Q1	1
KCNJ4	no assosiated desease in OMIM	0.347873315	Q2	0	Q1	1
TMEFF2	no assosiated desease in OMIM	0.180570916	Q2	2.375648373	Q3	1
HOXD12	no assosiated desease in OMIM	0.326860227	Q2	0	Q1	1
SP8	no assosiated desease in OMIM	14.28805695	Q4	0	Q1	1
IRX1	no assosiated desease in OMIM	0.699581101	Q2	0	Q1	1
AHR	no assosiated desease in OMIM	1.073458979	Q2	18.989716	Q4	1
FZD10	no assosiated desease in OMIM	0.693079814	Q2	0	Q1	1
SOX21	no assosiated desease in OMIM	3.895683918	Q3	0	Q1	1
POU4F1	Ataxia, intention tremor, and hypotonia syndrome, childhood-onset (619352)	0.116091441	Q2	0	Q1	1
KCNRG	no assosiated desease in OMIM	1.096758633	Q2	2.29134687	Q3	1
MAB21L1	Cerebellar, ocular, craniofacial, and genital syndrome (618479)	0.020324322	Q1	0.239092495	Q2	1
URAD	no assosiated desease in OMIM	0	Q1	0	Q1	1
GJA3	Cataract 14, multiple types (601885)	0.302291852	Q2	0.410142199	Q2	1
CCDC92	no assosiated desease in OMIM	0.46175619	Q2	12.87798908	Q4	1
LOC10798395[illegible]	no entry in OMIM		Q0		Q0	1
TBX3	Ulnar-mammary syndrome (181450)	0.157496498	Q2	29.92452065	Q4	1
LHX5	no assosiated desease in OMIM	0.085052021	Q2	0	Q1	1
PAH	Phenylketonuria (261600); [Hyperphenylalaninemia, non-PKU mild] (261600)	0.230964051	Q2	0	Q1	1
ANKS1B	no assosiated desease in OMIM	0.370766367	Q2	0.190904781	Q2	1
ATP2B1	no assosiated desease in OMIM	8.753290628	Q4	17.57034692	Q4	1
HOXC6	no assosiated desease in OMIM	0.026329235	Q1	0	Q1	1
ZIC2	Holoprosencephaly 5 (609637)	26.25085069	Q4	0.016482686	Q1	1
INSM2	no assosiated desease in OMIM	0	Q1	0	Q1	1
EPS8	no assosiated desease in OMIM	0.902561435	Q2	8.787371218	Q4	1
PRTG	no assosiated desease in OMIM	2.875604639	Q3	0.331114741	Q2	1
FOXC2	Lymphedema-distichiasis syndrome (153400); Lymphedema-distichiasis syndrome with renal disease and diabetes mellitus (153400)	0.20185747	Q2	1.327429114	Q2	1
IRF8	Immunodeficiency 32A, mycobacteriosis, autosomal dominant (614893); Immunodeficiency 32B, monocyte and dendritic cell deficiency, autosomal recessive (226990)	0.044389785	Q1	0	Q1	1

ZFHX3	Prostate cancer, somatic (176807)	0.47591436	Q2	6.453653327	Q4	1
BNC1	no assosiated desease in OMIM	0.061978909	Q1	1.439284575	Q3	1
UACA	no assosiated desease in OMIM	4.038231956	Q3	48.51256356	Q4	1
ALDH1A2	no assosiated desease in OMIM	0.062208796	Q1	0	Q1	1
PPP1R13B	no assosiated desease in OMIM	0.633450939	Q2	0.486885818	Q2	1
NKX2-1	Chorea, hereditary benign (118700); Choreoathetosis, hypothyroidism, and neonatal respiratory distress (610978); {Thyroid cancer, nonmedullary, 1} (188550)	0	Q1	0	Q1	1
GSC	Short stature, auditory canal atresia, mandibular hypoplasia, skeletal abnormalities (602471)	0.198764689	Q2	0	Q1	1
VSX2	Microphthalmia with coloboma 3 (610092); Microphthalmia, isolated 2 (610093)	0.038717434	Q1	0	Q1	1
SIX6	Optic disc anomalies with retinal and/or macular dystrophy (212550)	0.180252622	Q2	0.033658203	Q2	1
OTX2	Microphthalmia, syndromic 5 (610125); Pituitary hormone deficiency, combined, 6 (613986); Retinal dystrophy, early-onset, with or without pituitary dysfunction (610125)	31.49919889	Q4	0	Q1	1
LRFN5	no assosiated desease in OMIM	0.941968604	Q2	1.9288982	Q3	1
PAX9	Tooth agenesis, selective, 3 (604625)	0.01901603	Q1	0.023968063	Q2	1
HOXC11	no assosiated desease in OMIM	0	Q1	0	Q1	1
CCND2	Megalencephaly-polymicrogyria-polydactyly-hydrocephalus syndrome 3 (615938)	94.30760963	Q4	0.346915685	Q2	1
SLFN11	no assosiated desease in OMIM	0.108748028	Q2	7.86310564	Q4	1
JAK1	Autoinflammation, immune dysregulation, and eosinophilia (618999)	5.321104366	Q3	26.0264311	Q4	1
LHX4	Pituitary hormone deficiency, combined, 4 (262700)	0.793908498	Q2	1.009061969	Q2	1
LMX1A	Deafness, autosomal dominant 7 (601412)	0.022453561	Q1	0	Q1	1
MEF2D	no assosiated desease in OMIM	3.082561549	Q3	5.916149187	Q4	1
ALX3	Frontonasal dysplasia 1 (136760)	3.038087344	Q3	0.470258379	Q2	1
GFI1	Neutropenia, severe congenital 2, autosomal dominant (613107)	0.019467151	Q1	0.024536663	Q2	1
AK4	no assosiated desease in OMIM	9.922084486	Q4	0.089315809	Q2	1
FOXD3	{Autoimmune disease, susceptibility to, 1} (607836)	5.04975125	Q3	0	Q1	1
NR5A2	no assosiated desease in OMIM	1.452330089	Q2	0	Q1	1
NFIA	Brain malformations with or without urinary tract defects (613735)	0.449713922	Q2	0.215234737	Q2	1
CDKN2C	no assosiated desease in OMIM	0.01192147	Q1	5.890185457	Q4	1
FOXD2	no assosiated desease in OMIM	0.186410985	Q2	0.111504046	Q2	1
TAL1	Leukemia, T-cell acute lymphocytic, somatic (613065)	0.104939009	Q2	0	Q1	1
DMBX1	no assosiated desease in OMIM	0.117192013	Q2	0	Q1	1
RUNX3	no assosiated desease in OMIM	0.572157632	Q2	0.253036946	Q2	1
BRINP3	no assosiated desease in OMIM	0.010908937	Q1	0.192496829	Q2	1
RRP15	no assosiated desease in OMIM	10.60802616	Q4	4.247131584	Q3	1
ALX4	Frontonasal dysplasia 2 (613451); Parietal foramina 2 (609597); {Craniosynostosis 5, susceptibility to}	0.106276779	Q2	0.029767268	Q2	1
SHTN1	no assosiated desease in OMIM	0.836273597	Q2	3.724437173	Q3	1

WT1	Denys-Drash syndrome (194080); Frasier syndrome (136680); Meacham syndrome (608978); Mesothelioma, somatic (156240); Nephrotic syndrome, type 4 (256370); Wilms tumor, type 1	0.062380034	Q1	0.014976123	Q1	1
DBX1	no assosiated desease in OMIM	0	Q1	0.096212885	Q2	1
SOX6	Tolchin-Le Caignec syndrome (618971)	0.015775607	Q1	1.953913842	Q3	1
CALCA	no assosiated desease in OMIM	0	Q1	0	Q1	1
ARNTL	no assosiated desease in OMIM	0.458269378	Q2	3.380082212	Q3	1
IGF2	Silver-Russell syndrome 3 (616489)	0.126172156	Q2	1.60328253	Q3	1
TLX1	no assosiated desease in OMIM	0.010981934	Q1	0	Q1	1
MLLT10	Leukemia, acute myeloid 601626	1.901200845	Q3	6.222162875	Q4	1
PAX2	Glomerulosclerosis, focal segmental, 7 (616002); Papillorenal syndrome	0.006977185	Q1	0	Q1	1
NKX2-3	no assosiated desease in OMIM	0	Q1	0	Q1	1
HHEX	no assosiated desease in OMIM	0	Q1	2.765942688	Q3	1
ZNF503	no assosiated desease in OMIM	0.055964767	Q1	2.492369408	Q3	1
GAD2	no assosiated desease in OMIM	0.18037491	Q2	0	Q1	1
BMI1	no assosiated desease in OMIM	1.856026856	Q3	4.139566406	Q3	1
SPECC1	no assosiated desease in OMIM	4.041243192	Q3	14.00840609	Q4	1
HOXB3	no assosiated desease in OMIM	0.006641144	Q1	4.817854078	Q3	1
NXPH1	no assosiated desease in OMIM	0.029143113	Q1	0	Q1	1
POU4F2	no assosiated desease in OMIM	0	Q1	0	Q1	1
TMEM161B	no assosiated desease in OMIM	0.927419483	Q2	1.454671168	Q3	1
TBCA	no assosiated desease in OMIM	1.557352207	Q3	1.754951339	Q3	1
CDC20B	no assosiated desease in OMIM	0	Q1	0	Q1	1
MYO10	no assosiated desease in OMIM	14.5937325	Q4	12.82615775	Q4	1
PAX7	Myopathy, congenital, progressive, with scoliosis (618578); Rhabdomyosarcoma 2, alveolar (268220)	0	Q1	0	Q1	1
SCRG1	no assosiated desease in OMIM	0	Q1	0.729370943	Q2	1
NEUROG2	no assosiated desease in OMIM	0.025834195	Q1	0	Q1	1
PITX1	Clubfoot, congenital, with or without deficiency of long bones and/or mirror-image polydactyly (119800); Liebenberg syndrome (186550)	0.148760732	Q2	6.135418585	Q4	1
LEF1	Sebaceous tumors, somatic	0.404333626	Q2	0.282625351	Q2	1
ATOH1	no assosiated desease in OMIM	0.277384056	Q2	0	Q1	1
HERC5	no assosiated desease in OMIM	2.112926418	Q3	0.39897548	Q2	1
GSX2	Diencephalic-mesencephalic junction dysplasia syndrome 2 (618646)	0	Q1	0	Q1	1
SLIT2	no assosiated desease in OMIM	0.548739872	Q2	3.335694602	Q3	1
MAP3K13	no assosiated desease in OMIM	1.287399752	Q2	1.20976661	Q2	1
PRDM6	Patent ductus arteriosus 3 (617039)	0.093593151	Q2	0.047663065	Q2	1
NRG2	no assosiated desease in OMIM	0.024597337	Q1	0.023621196	Q2	1
FOXL2	Blepharophimosis, epicanthus inversus, and ptosis, type 1 (110100); Blepharophimosis, epicanthus inversus, and ptosis, type 2 (110100); Premature ovarian failure 3 (608996)	0.4354749	Q2	0.102116933	Q2	1
NT5E	Calcification of joints and arteries (211800)	0.021785695	Q1	270.312372	Q4	1
PDE10A	Dyskinesia, limb and orofacial, infantile-onset (616921); Striatal degeneration, autosomal dominant (616922)	0.495943713	Q2	0.166010351	Q2	1
TCF21	no assosiated desease in OMIM	0.105072018	Q2	16.47617364	Q4	1

EYA4	Deafness, autosomal dominant 10 (601316)	0.020028069	Q1	4.047398864	Q3	1
VGLL2	no assosiated desease in OMIM	0	Q1	0	Q1	1
REV3L	no assosiated desease in OMIM	0.976210789	Q2	14.28239599	Q4	1
PRDM1	no assosiated desease in OMIM	0.113426885	Q2	1.506803555	Q3	1
ZSCAN9	no assosiated desease in OMIM	2.143249383	Q3	2.587936994	Q3	1
POU4F3	Deafness, autosomal dominant 15 (602459)	0.049985452	Q1	0.084003128	Q2	1
ZNF184	no assosiated desease in OMIM	2.458208439	Q3	3.100161924	Q3	1
LOC10537510[illegible]	no entry in OMIM	0	Q1	0	Q1	1
NRN1	no assosiated desease in OMIM	0	Q1	0	Q1	1
FOXC1	Anterior segment dysgenesis 3, multiple subtypes (601631); Axenfeld-Rieger syndrome, type 3 (602482)	0.38937827	Q2	18.69629302	Q4	1
FOXF2	no assosiated desease in OMIM	0.16209274	Q2	13.62026032	Q4	1
NKX2-5	Atrial septal defect 7, with or without AV conduction defects (108900); Conotruncal heart malformations, variable (217095); Hypoplastic left heart syndrome 2 (614435); Hypothyroidism, congenital nongoitrous, 5 (25250); Tetralogy of Fallot (187500); Ventricular septal defect 3 (614432)	1.551639163	Q3	0	Q1	1
ZIC1	Structural brain anomalies with impaired intellectual development and craniosynostosis (618736)	0.141184295	Q2	0	Q1	1
GATA2	Emberger syndrome (614038); Immunodeficiency 21 (614172); {Leukemia, acute myeloid, susceptibility to} (601626); {Myelodysplastic syndrome, susceptibility to} (614286)	0.380647714	Q2	1.371723773	Q3	1
HOXB4	no assosiated desease in OMIM	0	Q1	9.010977977	Q4	1
ZADH2	no entry in OMIM	0.791588307	Q2	3.748306067	Q3	1
CRIM1	no assosiated desease in OMIM	3.113558109	Q3	260.5051802	Q4	1
NCOA1	no assosiated desease in OMIM	4.064599367	Q3	6.635015218	Q4	1
OSR1	no assosiated desease in OMIM	0.039127685	Q1	6.098877459	Q4	1
TSHZ3	no assosiated desease in OMIM	0.348748486	Q2	9.417856937	Q4	1
CD70	(618261)	0.226494867	Q2	0	Q1	1
RFX2	no assosiated desease in OMIM	0.152597052	Q2	0.792655018	Q2	1
ATP8B1	Cholestasis, benign recurrent intrahepatic (243300); Cholestasis, intrahepatic, of pregnancy, 1 (147480); Cholestasis, progressive familial intrahepatic 1 (211600)	0.241429511	Q2	10.04102097	Q4	1
FIGLA	Premature ovarian failure 6 (612310)	0	Q1	0	Q1	1
DSC3	Hypotrichosis and recurrent skin vesicles (613102)	0.316556132	Q2	0.599696577	Q2	1
CBX8	no assosiated desease in OMIM	0.945859894	Q2	1.252385579	Q2	1
TBX4	Amelia, posterior, with pelvic and pulmonary hypoplasia syndrome (601360); Ischiocoxopodopatellar syndrome with or without pulmonary arterial hypertension (147891)	0.007000332	Q1	0.023528838	Q2	1
TBX2	Vertebral anomalies and variable endocrine and T-cell dysfunction	0.00479724	Q1	15.75316477	Q4	1
DLX4	no assosiated desease in OMIM	0.127498498	Q2	0.02142678	Q1	1
PRAC2	no assosiated desease in OMIM	0.223122371	Q2	0	Q1	1
SIX2	no assosiated desease in OMIM	0.099717812	Q2	0	Q1	1

TLX2	no assosiated desease in OMIM	0.109819338	Q2	0	Q1	1
IGSF11	no assosiated desease in OMIM	0.915883375	Q2	0.011194103	Q1	1
RIPK4	CHAND syndrome (214350); Popliteal pterygium syndrome, Bartsocas-Papas type 1 (263650)	1.121535662	Q2	0.025643517	Q2	1
FILIP1L	no assosiated desease in OMIM	0.146306206	Q2	31.9637657	Q4	1
FRG2C	no entry in OMIM	0.041701584	Q1	0.056065329	Q2	1
PROK2	Hypogonadotropic hypogonadism 4 with or without anosmia (610628)	0.769211954	Q2	0	Q1	1
FEZF2	no assosiated desease in OMIM	0.125708093	Q2	0	Q1	1
SUSD5	no entry in OMIM	1.275691073	Q2	14.77752692	Q4	1
MKRN2	no assosiated desease in OMIM	9.079520841	Q4	7.90533443	Q4	1
OLIG1	no assosiated desease in OMIM	0.129624405	Q2	0	Q1	1
MGAT4A	no assosiated desease in OMIM	0.364280353	Q2	0.034982395	Q2	1
EYA2	no assosiated desease in OMIM	0.243609676	Q2	0.21723192	Q2	1
SLC32A1	no assosiated desease in OMIM	0.057384231	Q1	0	Q1	1
NKX2-2	no assosiated desease in OMIM	0.141308859	Q2	0	Q1	1
KIZ	Retinitis pigmentosa 69 (615780)	2.755156657	Q3	6.152141028	Q4	1
MTX2	Mandibuloacral dysplasia progeroid syndrome (619127)	11.45869172	Q4	1.908343637	Q3	1
PAX8	Hypothyroidism, congenital, due to thyroid dysgenesis or hypoplasia	0.385043617	Q2	1.49189191	Q3	1
LOC389895	no entry in OMIM	1.020305448	Q2	0	Q1	1
